# Design, Synthesis and Biological Evaluation of Novel Ketamine Derivatives as NMDAR Antagonists

**DOI:** 10.3390/molecules29112459

**Published:** 2024-05-23

**Authors:** Shiyun Li, Bin Wen, Wei Zhao, Lulu Wang, Xingquan Chen

**Affiliations:** 1 Qingyuan Innovation Laboratory, Quanzhou 362801, China; webi@tom.com; 2 Interdisciplinary Research Center on Biology and Chemistry, Shanghai Institute of Organic Chemistry, Chinese Academy of Sciences, Shanghai 201203, China; 3 College of Chemical Engineering, Fuzhou University, Fuzhou 350108, China; 15225801731@163.com (W.Z.); 15980279040@163.com (L.W.)

**Keywords:** ketamine, depression, NMDAR, antidepressants

## Abstract

Depression is a chronic, severe, and often life-threatening neurological disorder. It not only causes depression in patients and affects daily life but, in severe cases, may lead to suicidal behavior and have adverse effects on families and society. In recent years, it has been found that sub-anesthetic doses of ketamine have a rapid antidepressant effect on patients with treatment-resistant depression and can significantly reduce the suicidal tendencies of patients with major depressive disorder. Current studies suggest that ketamine may exert antidepressant effects by blocking NMDAR ion channels, but its anesthetic and psychotomimetic side effects limit its application. Here, we report efforts to design and synthesize a novel series of ketamine derivatives of NMDAR antagonists, among which compounds **23** and **24** have improved activity compared with ketamine, introducing a new direction for the development of rapid-acting antidepressant drugs.

## 1. Introduction

Depression is a mental illness characterized by low mood. There are three main symptoms of depression: low mood or depression, anhedonia, and lack of energy/fatigue [1]. Patients with major depression exhibit suicidal behaviors, negatively impacting families and society [2,3]. According to the statistics of the World Health Organization, there are more than 350 million people with depression worldwide, and the incidence rate is about 4.4%, with female patients being twice as representative as men [4]. Depression is the most common psychiatric disorder among suicidal individuals [5,6], and treatment of depression significantly reduces the risk of suicide [7,8]. Depression is currently mainly treated with antidepressant drugs, supplemented by psychotherapy or physical therapy. Due to the development of antidepressants in recent years, there are now many interactions between different drugs; therefore, standardized treatment procedures are particularly important for depression treatment. The monoamine hypothesis of depression posits that depressed patients have decreased concentrations of serotonin (5-HT), norepinephrine (NE), and dopamine (DA). Traditional antidepressants are developed based on this hypothesis and mainly include monoamine oxidase inhibitors (MAOIs), selective serotonin reuptake inhibitors (SSRIs), serotonin norepinephrine reuptake inhibitors (SNRIs), and so on [9,10].

**Figure 1:**
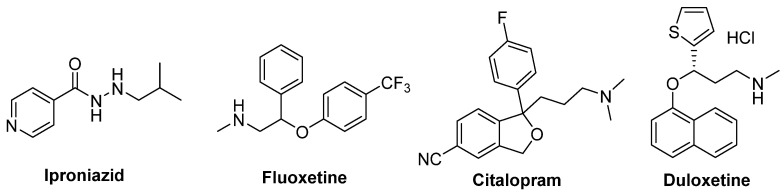
Common traditional antidepressants.

Common antidepressants are shown in Figure 1. These traditional antidepressants are first-line drugs for the treatment of depression and can relieve the depressive symptoms of most patients with depression, but their effectiveness is still not high. The currently available antidepressants have certain limitations, such as long time lags, low response rates, and ineffectiveness for approximately one-third of patients with treatment-resistant depression [11,12]. In addition, patients with major depressive disorder (MDD) have persistent depression and often have suicidal thoughts and behaviors [13]. The time between the development of suicidal ideation to the enactment of suicide is very short, so traditional antidepressants are also ineffective for MDD patients. The suicide rate among patients with MDD is approximately 21 times higher than that for patients with general depression [14]. Thus, there exists an unmet medical need for rapidly acting novel antidepressants that are also effective for treatment-resistant patients and patients with suicidal ideation. To sum up, it is very important and necessary to develop rapidly acting antidepressants for MDD or treatment-resistant depression (TRD) patients.

Low, sub-anesthetic doses of (*R*, *S*)-ketamine rapidly improve the core symptoms of depression, including low mood, anhedonia, and suicidal ideation. It works within 2 h of the administration of a single dose, and the antidepressant effect lasts for a week. Accumulating evidence has revealed the robust antidepressant effects and anti-suicidal effects of ketamine, an N-methyl-D-aspartate receptor (NMDAR) antagonist, on treatment-resistant patients with MDD or TRD. The discovery of the robust antidepressant actions of ketamine is regarded as the greatest advancement in mood disorder research in the past 60 years [15,16]. In 2019, S-ketamine was approved for marketing by the FDA. However, ketamine poses a risk of abuse due to its ability to induce anesthesia and psychotomimetic side effects. Secondly, ketamine has poor oral bioavailability, only between 20 and 25%, so it is usually administered intravenously and nasally [17,18]. Current studies suggest that ketamine exerts antidepressant effects by blocking the ion channels of NMDA receptors [19,20,21,22]. Therefore, we hope to modify the structure of ketamine and expect to find candidate compounds with good antidepressant effect and less-toxic side effects.

**Figure 2:**
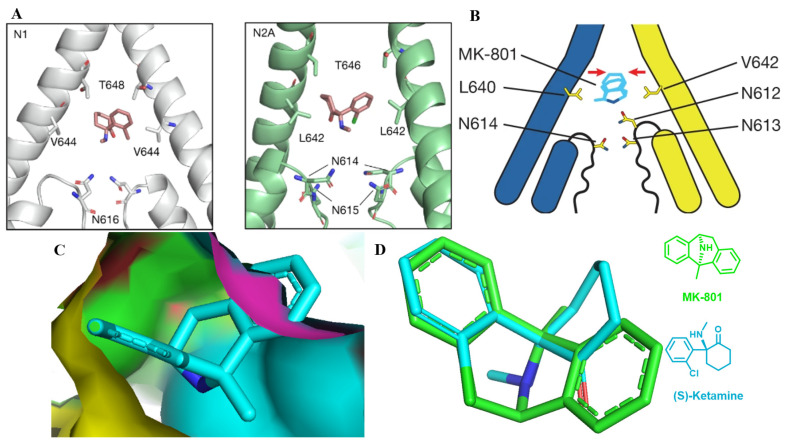
Molecular design process of ketamine derivatives. (**A**) Interaction of ketamine with NMDAR (GluN1/GluN2A subunits). (**B**) Interaction of MK-801 and NMDAR (GluN1/GluN2B subunits). (**C**) The docking results regarding the docking of MK-801 with NMDAR. (**D**) Molecular superposition of ketamine and MK-801, where the green solid line indicates MK-801, and the blue solid line indicates ketamine.

In July 2021, Zhu Shujia and her collaborators analyzed the cryo-EM structures of ketamine and NMDAR with a resolution of 3.5 Å [23]. Ketamine binds in the cavity between the voltage-gated and selectivity filter of an ion channel. NMDAR has seven subunits, which are encoded by seven genes: one GRIN1 gene encodes GluN1, four GRIN2 genes encode GluN2A-D, and two GRIN3 genes encode GluN3A-B [24]. Two amino acids, leucine 642 on GluN2A and asparagine 616 on GluN1, were identified as key residues for forming hydrophobic and hydrogen-bonding interactions with ketamine. We also analyzed the crystal structures of MK-801 and NMDAR. It can be seen that MK-801 binds to NMDAR mainly through hydrogen bonds and hydrophobic interactions [25]. However, there exists a clash between MK-801 and NMDAR. Therefore, the design of compounds cannot be carried out by means of molecular docking. It was discovered that NMDAR channel blockers, such as ketamine, MK-801, and PCP (Phencyclidine) molecules, are relatively small. It is speculated that they may bind to NMDAR ion channels in a similar conformation. To find a molecular skeleton similar to that of MK-801, an approximate conformation search method was used. Open3DALIGN, an open-source software, was used to overlap the conformations of ketamine and MK-801 in the crystal structure based on the pharmacophore. It was found that ketamine and MK-801 fit together well (Figure 2). The next step involved using the strategy based on pharmacophore and molecular skeleton superposition to design ketamine derivatives. The aim was to find ketamine derivatives with an antagonistic effect on NMDAR.

## 2. Results

### 2.1. Molecule Design

**Figure 3:**
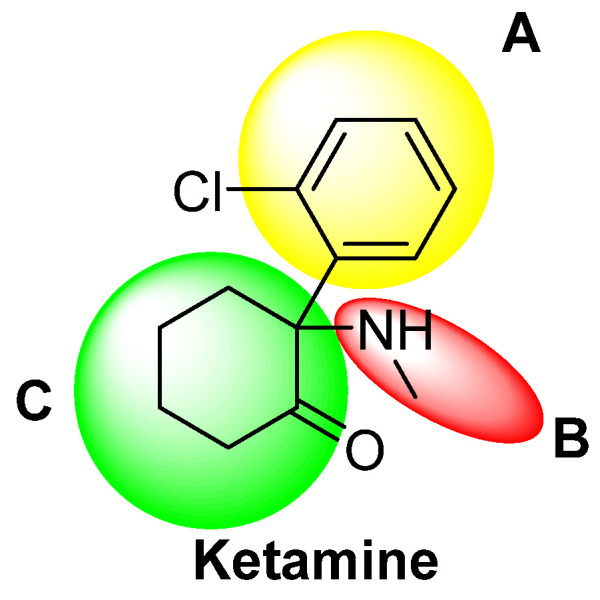
Schematic diagram of structural modification of ketamine.

There are three main aspects of the structural transformation of ketamine (Figure 3): The aromatic ring at A can be replaced by other aromatic rings or some simple substitutes on the benzene ring; however, the simple substituent group of phenyl was adopted by GILGAMESH PHARMACEUTICALS, INC, which filed patents on it, so we had to try to complicate the aromatic ring to breakthrough the patent space. The methylamino group at B can be replaced with other aliphatic chains; it has been reported that aliphatic chains such as esters and amides serve as short-acting anesthetics, but the antagonism to NMDAR is weak [26,27]. The aliphatic ring at C can be made into a benzo aliphatic ring, or the six-membered ring can be enlarged or reduced.

### 2.2. Synthesis of and Electrophysiological Results Obtained for Ketamine Derivatives

**Figure 4:**
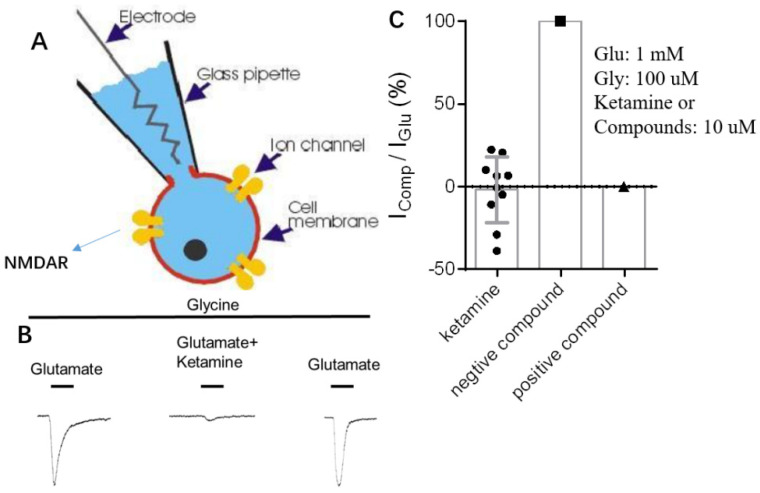
Schematic diagram of electrophysiological experiments. Note: (**A**) Schematic diagram illustrating the electrophysiological experimental detection device. (**B**) Alterations in detected current following the addition of agonists and compounds. (**C**) Statistical analysis of current changes before and after compound administration. The specific experimental procedure involved assessing the impact of drugs on NMDA-receptor-mediated current through whole-cell electrophysiological recording. NMDA receptor currents were induced by glutamate and glycine in HEK293 cells expressing the NMDA receptor. I_Comp_ represents the current induced by glutamate and glycine in the presence of ketamine and its derivatives, while I_Glu_ denotes the current induced solely by glutamate and glycine.

The activity of all ketamine derivatives was tested using HEK293 cells expressing human NMDAR (GluN1 and GluN2A subunits; GluN1 and GluN2B subunits), and the current changes before and after administration (10 µM) were recorded using the cell patch clamp technique. Then, we calculated the ratio of the current after administration and before administration; the smaller the ratio, the stronger the antagonistic effect of the ketamine derivatives on NMDAR (Figure 4).

### 2.3. Structural Modification of Aromatic Region of Segment A

**Scheme 1:**
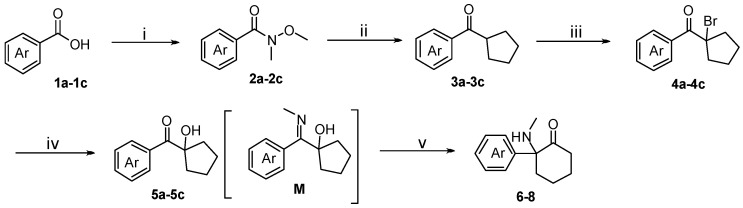
Synthesis scheme of structural modification of aromatic region in segment A. Reagents and conditions: (**i**) CH_3_NHOCH_3_·HCl, HATU, Et_3_N, and CH_2_Cl_2_ (rt); (**ii**) cyclopentylmagnesium bromide and THF (−78 °C); (**iii**) CuBr_2_ and EtOAc (80 °C); (**iv**) KOH and MeOH; (**v**) CH_3_NH_2_·H_2_O and decalin (170 °C).

Firstly, we carried out the structural modification of the aromatic region of segment A. We made some simple substituents on the benzene ring or replaced the benzene ring with a more complex aromatic ring. The synthesis of compounds **6**–**8** started with the condensation of carboxylic acid and hydroxylamine hydrochloride to obtain Weinreb amides (**2**). Then, a reaction with Grignard reagent was induced to obtain a ketone (**3**), followed by the α-bromination of the carbonyl group with copper bromide (**4**) and hydrolysis with potassium hydroxide solution to obtain a key intermediate α-hydroxy ketone (**5**). In most of the literature that has been reported, α-hydroxy ketones are usually converted to α-hydroxy imines first (**M**), followed by a similar semi-pinacol rearrangement [28,29]. The innovative facet of our designed route is the direct rearrangement of α-hydroxy ketone and methylamine at 170 °C to obtain the target products, **6**–**8** (Scheme 1, Table 1).

**Figure 5:**
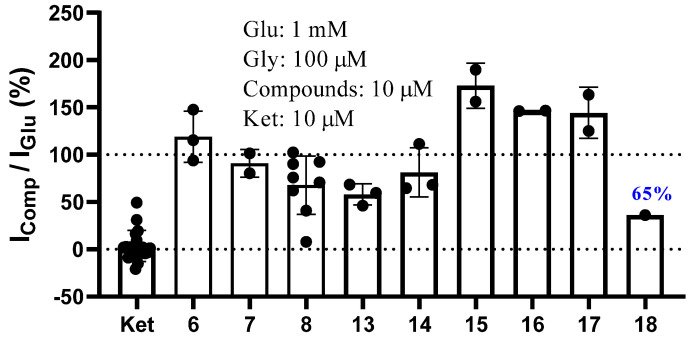
Inhibitory effect of ketamine and ketamine derivatives **6**–**8** and **11**–**18** on NMDAR-mediated current.

The inhibitory effect of compounds **6**–**8** on NMDAR-mediated current was tested. The results showed that, compared with ketamine (Ket), these compounds had no significant inhibitory effect on NMDAR-mediated current (Figure 5). It may be that the steric hindrance of these compounds is too great; these results indicate that the volume of ketamine derivatives should not be too large.

### 2.4. Alkylation Modification of Segment B

**Scheme 2:**
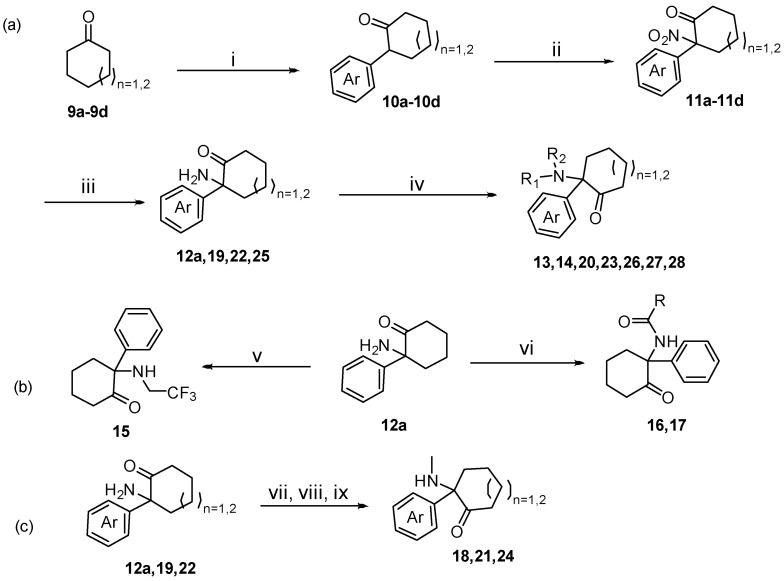
Synthesis scheme of alkylation modification of segment B. Reagents and conditions: (**i**) Pd_2_(dba)_3_, Xantphos, 1,4-dioxane, 100 °C; (**ii**) Ceric ammonium nitrate (CAN), Cu(OAc)_2_, DCE, 80 °C; (**iii**) Zn, HOAc, 80 °C; (**iv**) RCHO, NaBH_3_CN, HOAc, MeOH; (**v**) CF_3_CH_2_OTf, Et_3_N, DMF, 90 °C; (**vi**) anhydride, Et_3_N, CH_2_Cl_2_; (**vii**) (Boc)_2_O, Et_3_N, THF, 70 °C; (**viii**) LiAlH_4_, THF, 70 °C; (**ix**) Jones reagent, acetone.

The next step was the alkylation modification of segment B (Scheme 2). The synthetic route first involved a palladium-catalyzed coupling reaction of aryl halide and cyclic ketone to obtain 2-arylcycloketone, which was then nitrated with ceric ammonium nitrate (CAN) and reduced with zinc powder to obtain the key intermediate amino compound [30], followed by an alkylation reaction to obtain the target product (Scheme 2a). In the process of methylation with formaldehyde solution or paraformaldehyde, the product monomethylated and demethylated, making it difficult to separate and purify. However, when the formaldehyde was more than two equivalents, the product was entirely dimethylated.

The monomethylated products (**18**, **21**, **24**) were synthesized by protecting the amino compound with Boc anhydride, followed by reduction with lithium aluminum hydride to attain the N-methylated product and oxidation with Jones reagent (Scheme 2c). The ketamine derivatives of modified segment B are shown in Table 1.

Subsequently, we performed patch clamp electrophysiological experiments on compounds **13**–**18**. Interestingly, compounds **16**–**18** exhibited obvious activity (Figure 5). Among the compounds, the inhibition rate of compound **18** reached 65%, the difference with ketamine is that there is no chlorine atom. The ethylated compound **13** and the dimethylated compound **14** had poor activities. Unfortunately, compounds **15**–**17** exhibited no biological activity whatsoever, indicating that trifluoroethyl and amides had no inhibitory effect on NMDAR, and it was also revealed that the exposed nitrogen atom plays a very important role in maintaining the activity of these compounds. These results are similar to those reported in William A. Denny’s work showing that ketamine esters and amides had poor NMDAR antagonism [26].

### 2.5. Structural Modification of Aliphatic Ring to a Seven-Membered Ring

In the next step, the aliphatic ring of ketamine was modified by enlarging it, transforming it from a six-membered ring into a seven-membered ring. Compounds **19**–**28** were synthesized using Scheme 2, and their structures are shown in Table 2.

**Figure 6:**
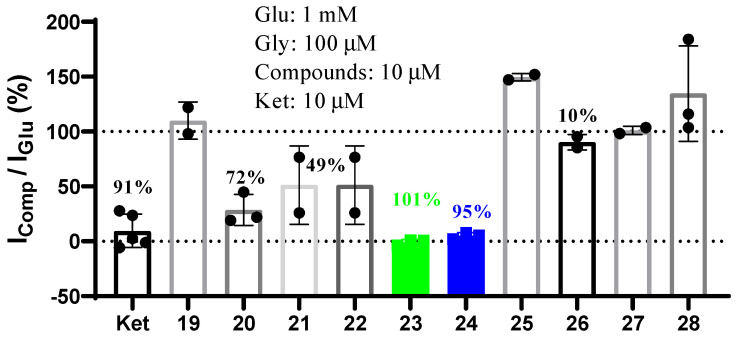
Inhibitory effect of ketamine and ketamine derivatives **19**–**28** on NMDA-receptor-mediated current.

When the aromatic ring is a benzene ring, except for compound **19**, almost all the other seven-membered ring compounds have inhibitory effects on NMDAR (Figure 6). Among them, 2-(2-chlorophenyl)-2-(ethylamino)cycloheptan-1-one (**23**) and 2-(2-chlorophenyl)-2-(methylamino)cycloheptan-1-one (**24**) have excellent activity, with inhibition rates of 101% and 95%, respectively, which are even better than the activity of ketamine (with an inhibition rate of 91%). In addition, the inhibition rates of compounds **20** and **21** for NMDAR were 72% and 49%, respectively. The difference of compounds **20** and **23** were no chloride atoms. Meanwhile, compound **19** had almost no activity, maybe due to no chloride atom, while compound **22** inhibited NMDAR by 49%. These results show that for seven-membered ring compounds, *N*-ethyl-substituted compounds have good activity, and the chloride atom in this skeleton is important for the inhibitory effect on NMDAR.

However, when the benzene ring was changed into a naphthalene ring, there was almost no inhibitory effect on NMDAR (**25**–**28**); in this regard, compound **26** had an inhibition rate of only 8%. These results suggest that compounds containing a naphthalene ring may not be able to enter the ion channel located in the transmembrane region of NMDAR. Therefore, the size of the aromatic ring should not exceed a certain volume.

### 2.6. Structural Modification of Aliphatic Ring to Benzo Series

**Scheme 3:**
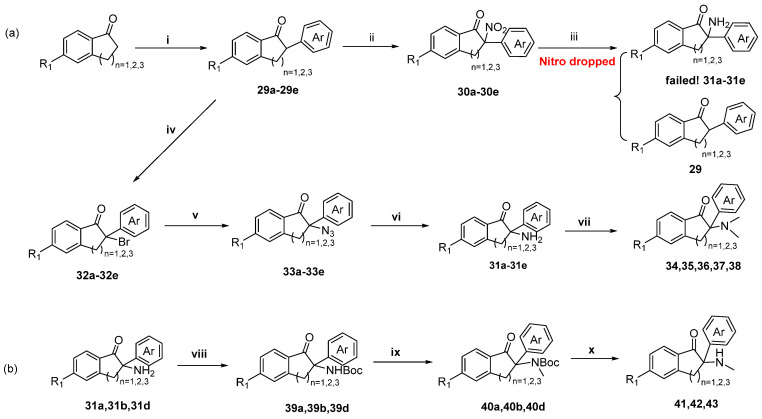
Synthesis scheme for the structural modification of six-membered rings into benzo series. Reagents and conditions: (**i**) Pd_2_(dba)_3_, DtBPF, THF, 70 °C; (**ii**) CAN, Cu(OAc)_2_, DCE, 80 °C; (**iii**) Zn, HOAc, 80 °C; (**iv**) CuBr_2_, EtOAc, 80 °C; (**v**) NaN_3_, DMSO; (**vi**) Pd/C, H_2_, MeOH; (**vii**) HCHO, HOAc, NaBH_3_CN, MeOH; (**viii**) (Boc)_2_O, Et_3_N, THF, 70 °C; (**ix**) NaH, MeI; (**x**) HCl/dioxane.

In order to further break through the patent space, we modified the aliphatic ring of ketamine into a benzo series, such as a benzo five-membered ring (indane), a benzo six-membered ring (tetralin), and a benzo seven-membered ring (benzocycloheptane). The synthesis route is similar to Scheme 2, but in the process of the reduction of nitro products, we found that the intermediate is unstable, causing the nitro to fall off and become the product of the previous step, so the synthesis route was changed to obtain compounds **34**–**38** and **41**–**43** (Scheme 3, Table 3).

Scheme 3a begins with the palladium-catalyzed coupling reaction of aryl halide and benzocyclohexanone, followed bromination with cuprous bromide for the carbonyl α-site, nucleophilic substitution with sodium azide, palladium–carbon–hydrogen reduction to obtain the key intermediate amino compound, and then nitrogen alkylation to obtain the final products **34**–**38**. Scheme 3b is similar to Scheme 2b in that this route allows the synthesis of monomethyl compounds **41**–**43**.

**Figure 7:**
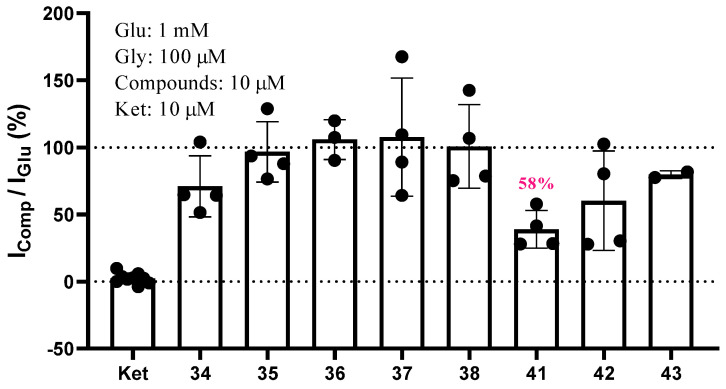
Inhibitory effect of ketamine and ketamine derivatives **34**–**38** and **41**–**43** on NMDA-receptor-mediated current.

Among the synthesized compounds, compounds **34** and **41**–**43** have certain inhibitory effects on NMDAR (Figure 7). By comparing compounds **34** and **41** as well as **35** and **42**, it can be seen that the inhibition effect of monomethyl compounds on NMDAR is better than that of dimethyl compounds, and the inhibition rate of compound **41** on NMDAR is the highest (58%). These results show that, on the one hand, nitrogen atoms are very important pharmacophores, and an exposed nitrogen atom is important for maintaining the inhibitory activity of NMDAR. On the other hand, the larger volume of this compound compared with that of ketamine results in a decrease in the inhibitory activity of NMDAR. Subsequent modifications should maintain the exposed nitrogen atoms while reducing the volume of the compound.

## 3. Materials and Methods

### 3.1. Chemistry

All coupling reactions were carried out under the protection of argon, strictly in accordance with anhydrous and oxygen-free operation. All raw materials were commercial reagents or prepared using methods reported in the literature. The reactions were monitored using UV light, phosphomolybdic acid, potassium permanganate, or iodine bath. All compounds were purified via column chromatography, and the final products were purified using HPLC, acidified using 10% dilute hydrochloric acid solution, and freeze-dried using a lyophilizer. All types of Nuclear magnetic resonance spectrometer (NMR) data were acquired with a Bruker AV-400 (Bruker, Billerica, MA, USA) (400 MHz for ^1^H NMR and 101 MHz for ^13^C NMR). Chemical shifts (δ) are given in ppm, and all spectra were corrected with solvent peaks as follows: Chloroform-d 7.26 ppm H, 77.16 ppm C; DMSO-d_6_ 2.50 ppm H, 39.52 ppm C. The unit used for the coupling constant (J) is hertz (Hz), and the divisions were abbreviated as follows: s, singlet; d, doublet; t, triplet; q, quartet; dd, doublet of doublet; dt, doublet of triplet; m, multiplet. Yield refers to the yield after purification. All compounds subjected to biological testing were more than 95% pure. Low-resolution ionization mass spectrometry (ESI-MS) was performed with Agilent 1260/6120 ES-LCMS system (Agilent, Santa Clara, CA, USA) or Shimadzu LCMS 2020 (Shimadzu, Kyoto, Japan). High-resolution mass spectra (HRMS) were collected with Thermo Fisher Scientific LTQ FTICR-MS or Thermo Scientific Q Exactive HF Orbitrap-FTMS (Thermo Fisher Scientific, Waltham, MA, USA).

#### 3.1.1. *N*,3,4-Trimethoxy-*N*-methylbenzamide [30] (**2a**)

To a solution of 3,4-dimethoxybenzoic acid (10 g, 54.9 mmol) in CH_2_Cl_2_ (100 mL) in an ice bath, we added *N*,*O*-dimethylhydroxylamine hydrochloride (6.42 g, 65.9 mmol), HATU (25 g, 65.9 mmol), and TEA (38 mL, 275 mmol). The mixture was stirred at room temperature for 3 h and then concentrated under reduced pressure. The residue mixture was diluted with ethyl acetate (100 mL) and washed with saturated ammonium chloride, saturated sodium bicarbonate, and saturated brine solutions successively. The organic layers were dried with anhydrous sodium sulfate, concentrated, and purified via silica gel column chromatography (20% EtOAc in Pet. Ether), yielding 11.3 g (yield 91%) of light-yellow solid (**2a**). ESI-MS: *m*/*z* = 226.1 (M + H)^+^. ^1^H NMR (400 MHz, CDCl_3_) δ 7.38 (dd, *J* = 8.4, 2.0 Hz, 1H), 7.31 (d, *J* = 1.9 Hz, 1H), 6.86 (d, *J* = 8.4 Hz, 1H), 3.90 (d, *J* = 5.0 Hz, 6H), 3.57 (s, 3H), 3.35 (s, 3H).

#### 3.1.2. Cyclopentyl(3,4-dimethoxyphenyl)methanone [31] (**3a**)

Cyclopentyl magnesium bromide (13.3 mL, 26.6 mmol, 2.0 M in THF) was added dropwise to a solution of **2a** (2 g, 8.9 mmol) in THF (30 mL) at −78 °C. After 30 min, the low-temperature equipment was removed, and the mixture was stirred at rt for 2 h. The mixture was then quenched by dropwise addition of a saturated ammonium chloride solution at 0 °C and extracted with EtOAc, dried over Na_2_SO_4_, and concentrated under reduced pressure. The residue mixture was purified via silica gel column chromatography (5% EtOAc in Pet. Ether), yielding 1.2 g (yield 57.7%) of **3a** in the form of a colorless oil. ESI-MS: *m*/*z* = 235.1 (M + H)^+^. ^1^H NMR (400 MHz, CDCl_3_) δ 7.63–7.55 (m, 2H), 6.90 (d, *J* = 8.4 Hz, 1H), 3.95 (d, *J* = 3.9 Hz, 6H), 3.70 (p, *J* = 7.9 Hz, 1H), 1.96–1.86 (m, 4H), 1.80–1.59 (m, 4H).

#### 3.1.3. (1-Bromocyclopentyl)(3,4-dimethoxyphenyl)methanone [32] (**4a**)

Cupric bromide (476 mg, 2.13 mmol) was added to a solution of **3a** (200 mg, 0.85 mmol) in EtOAc (5 mL). The mixture was refluxed at 80 °C for 2h. Then, the reaction mixture was directly concentrated and purified via silica gel column chromatography (5% EtOAc in Pet. Ether), yielding **4a** (0.4 g, yield 86%) as a yellow oil. ^1^H NMR (400 MHz, CDCl_3_) δ 7.93 (dd, *J* = 8.5, 2.1 Hz, 1H), 7.69 (d, *J* = 2.1 Hz, 1H), 6.89 (d, *J* = 8.5 Hz, 1H), 3.95 (d, *J* = 7.3 Hz, 6H), 2.56–2.40 (m, 4H), 2.13–2.00 (m, 2H), 1.87–1.73 (m, 2H).

#### 3.1.4. 2-(3,4-Dimethoxyphenyl)-2-(methylamino)cyclohexan-1-one (**6**)

KOH (90 mg, 1.6 mmol) was added to a solution of **4a** (0.25 g, 0.8 mmol) in MeOH (5 mL). The mixture was stirred at rt for 5h, diluted with EtOAc (20 mL), washed with saturated aqueous NaCl, and dried over Na_2_SO_4_. It was then concentrated and purified using silica gel column chromatography (5% EtOAc in Pet. Ether) to attain **5a** (0.13 g, yield 65%) in the form of a colorless oil. ESI-MS: *m*/*z* = 251.1 (M + H)^+^.

Methylamine aqueous solution (40%) was added to a solution of **5a** (50 mg, 0.2 mmol) in decahydronaphthalene (2 mL) in a 15 mL sealed tube. The mixture was stirred at 170 °C for 6 h. After cooling, it was extracted with EtOAc and concentrated. The mixture was purified using prep-HPLC and then acidified with 10% HCl and lyophilized to obtain **6** (21 mg, 40%) as white solid. ^1^H NMR (400 MHz, CDCl_3_) δ 10.15 (s, 1H), 9.91 (s, 1H), 7.14 (d, *J* = 1.9 Hz, 1H), 7.00 (d, *J* = 8.2 Hz, 1H), 6.92 (d, *J* = 8.3 Hz, 1H), 3.98 (s, 3H), 3.91 (s, 3H), 3.04 (d, *J* = 13.4 Hz, 1H), 2.69–2.32 (m, 6H), 2.08–1.76 (m, 4H). ^13^C NMR (101 MHz, CDCl_3_) δ 204.80, 150.32(2C), 122.25, 120.90, 71.94, 56.63, 55.98, 39.23, 32.61, 27.08, 27.03, 22.00. ESI-MS: *m*/*z* = 264.1 (M + H)^+^. HRMS *m*/*z*: calcd for C_15_H_20_NO_3_ 264.1594; found, 264.1599.

#### 3.1.5. 2-(2,3-Dihydrobenzo[b][1,4]dioxin-6-yl)-2-(methylamino)cyclohexan-1-one (**7**)

Compounds **7** and **8** were synthesized in a process similar to that described for the preparation of **1**. ^1^H NMR (400 MHz, CDCl_3_) δ 10.14 (s, 1H), 9.69 (s, 1H), 6.98 (d, *J* = 19.6 Hz, 3H), 4.28 (s, 4H), 3.05 (d, *J* = 13.1 Hz, 1H), 2.66–2.33 (m, 6H), 2.03–1.64 (m, 4H). ^13^C NMR (101 MHz, CDCl_3_) δ 204.64, 145.04, 144.31, 122.98, 121.78, 118.68, 117.64, 71.56, 64.40, 64.25, 39.20, 33.03, 27.44, 27.08, 21.76.

ESI-MS: *m*/*z* = 262.1 (M + H)^+^. HRMS *m*/*z*: calcd for C_15_H_20_NO_3_ 262.1438; found, 262.1445.

#### 3.1.6. 2-(Benzo[d][1,3]dioxol-5-yl)-2-(methylamino)cyclohexan-1-one (**8**)

The result was a white solid. ^1^H NMR (400 MHz, DMSO-*d*_6_) δ 10.09 (dd, *J* = 11.5, 5.4 Hz, 1H), 9.31 (dd, *J* = 11.4, 5.5 Hz, 1H), 7.07 (d, *J* = 8.2 Hz, 1H), 7.01 (d, *J* = 1.8 Hz, 1H), 6.83 (dd, *J* = 8.2, 1.9 Hz, 1H), 6.12 (dd, *J* = 3.5, 0.8 Hz, 2H), 5.98 (s, 1H), 3.15–3.01 (m, 1H), 2.42–2.29 (m, 2H), 2.12 (q, *J* = 5.6 Hz, 4H), 2.03–1.76 (m, 2H), 1.69–1.51 (m, 2H). ^13^C NMR (101 MHz, DMSO-*d_6_*) δ 206.34, 148.98, 148.82, 124.11, 123.33, 109.49, 108.70, 102.39, 71.15, 31.74, 27.32, 26.94, 21.66. ESI-MS: *m*/*z* = 248.1 (M + H)^+^. HRMS *m*/*z*: calcd for C_14_H_18_NO_3_ 248.1281; found, 248.1288.

#### 3.1.7. 2-Phenylcycloheptan-1-one [33] (**10b**)

Pd_2_(dba)_3_ (292 mg, 0.32 mmol), Xantphos (370 mg, 0.64 mmol), and cesium carbonate (22.8 g, 70 mmol) were dissolved in 1,4-dioxane (50 mL). After the air was replaced with N_2_, bromobenzene (5 g, 32 mmol) and cycloheptanone (7.17 g, 64 mmol) were added. The mixture was stirred at 100 °C for 16 h. After cooling, it was poured into water (50 mL) and extracted with EtOAc (50 mL × 3). The combined organic layers were dried with anhydrous Na_2_SO_4_, concentrated, and purified via silica gel column chromatography (3% EtOAc in Pet. Ether) to obtain **10b** (7.55 g, 100%) as yellow oil. ^1^H NMR (400 MHz, CDCl_3_) δ 7.35–7.29 (m, 2H), 7.26–7.20 (m, 3H), 3.72 (dd, *J* = 11.4, 4.2 Hz, 1H), 2.74–2.65 (m, 1H), 2.57–2.48 (m, 1H), 2.19–2.11 (m, 1H), 2.09–1.91 (m, 4H), 1.71–1.59 (m, 1H), 1.52–1.40 (m, 2H). ESI-MS: *m*/*z* = 189.1 (M + H)^+^.

#### 3.1.8. 2-Nitro-2-phenylcyclohexan-1-one [30] (**11a**)

Copper(II) acetate (211 mg, 1.16 mmol) and ceric ammonium nitrate (8 g, 14.5 mmol) were added to a solution of 2-phenylcyclohexan-1-one (2 g, 11.48 mmol) in DCE (20 mL) in a nitrogen atmosphere. The mixture was stirred at 80 °C for 16 h. The mixture was filtrated using celite, and the filtrate was concentrated and purified via silica gel column chromatography (5% EtOAc in Pet. Ether), yielding **11a** (0.49 g, yield 33%) as yellow oil. ^1^H NMR (400 MHz, CDCl_3_) δ 7.49–7.43 (m, 3H), 7.39–7.32 (m, 2H), 3.11–3.02 (m, 1H), 2.94–2.85 (m, 1H), 2.71–2.63 (m, 1H), 2.60–2.51 (m, 1H), 1.99–1.87 (m, 3H), 1.83–1.70 (m, 1H).

#### 3.1.9. 2-Amino-2-phenylcyclohexan-1-one [34] (**12a**)

Zinc dust (1.09 g, 16.83 mmol) was added to a solution of **11a** (1.23 g, 5.61 mmol) in HOAc (10 mL). The mixture was stirred at 80 °C for 10h; then, it was concentrated, and its pH was rendered basic with 10% sodium hydroxide solution. The mixture was filtrated, and the filtrate was extracted with EtOAc 3 times. The combined organic layers were concentrated under reduced pressure and purified using silica gel column chromatography (50% EtOAc in Pet. Ether), yielding **12a** (0.72g, 67.8%) as colorless oil. ^1^H NMR (400 MHz, CDCl_3_) δ 7.41–7.35 (m, 2H), 7.32–7.24 (m, 3H), 2.92–2.81 (m, 1H), 2.50–2.34 (m, 2H), 2.04–1.90 (m, 3H), 1.84–1.63 (m, 4H). ESI-MS: *m*/*z* = 190.1 (M + H)^+^.

#### 3.1.10. 2-(Ethylamino)-2-phenylcyclohexan-1-one hydrochloride (**13**)

To a solution of **12a** (82 mg, 0.43 mmol) in MeOH (3 mL), we added acetaldehyde (29 μL, 0.52 mmol), HOAc (25 μL, 0.43 mmol), and NaBH_3_CN (41 mg, 0.65 mmol). The mixture was stirred at rt for 1.5 h and then extracted with EtOAc and washed with saturated brine. The combined organic layers were concentrated and purified using prep-HPLC and then acidified using 10% HCl and lyophilized to obtain **13** (56 mg, 60%) as white solid. ^1^H NMR (400 MHz, CDCl_3_) δ 7.37 (t, *J* = 7.5 Hz, 2H), 7.31–7.21 (m, 3H), 2.94–2.85 (m, 1H), 2.44–2.37 (m, 1H), 2.36–2.24 (m, 2H), 2.14 (s, 1H), 2.08–2.00 (m, 1H), 1.98–1.92 (m, 1H), 1.89–1.69 (m, 4H), 0.99 (t, *J* = 7.1 Hz, 3H). ^13^C NMR (101 MHz, CDCl_3_) δ 211.46, 139.38, 128.82 (2C), 127.43, 127.01 (2C), 69.79, 39.74, 36.58, 36.01, 27.70, 22.34, 15.65. ESI-MS: *m*/*z* = 218.1 (M + H)^+^. HRMS *m/z*: calcd for C_14_H_20_NO 218.1548; found, 218.1548.

#### 3.1.11. 2-(Dimethylamino)-2-phenylcyclohexan-1-one (**14**)

Compound **14** was synthesized from **12a** and HCHO (37% in water, 2.5eq) in a process similar to that described for the preparation of **13**. The result was a white solid. ^1^H NMR (400 MHz, CDCl_3_) δ 12.05 (s, 1H), 7.58–7.46 (m, 5H), 3.34 (d, *J* = 12.6 Hz, 1H), 2.85 (d, *J* = 4.3 Hz, 3H), 2.64 (d, *J* = 4.3 Hz, 3H), 2.59–2.52 (m, 2H), 2.51–2.41 (m, 1H), 2.02–1.88 (m, 2H), 1.86–1.71 (m, 1H), 1.63–1.47 (m, 1H). ^13^C NMR (101 MHz, CDCl_3_) δ 205.36, 130.89(2C), 130.15, 130.02(2C), 129.54, 77.87, 40.77, 40.30, 40.27, 33.11, 26.94, 21.76. ESI-MS: *m*/*z* = 218.2 (M + H)^+^. HRMS *m*/*z*: calcd for C_14_H_20_NO 218.1539; found, 215.1546.

#### 3.1.12. 2-Phenyl-2-((2,2,2-trifluoroethyl)amino)cyclohexan-1-one (**15**)

TEA (80 μL) and 2,2,2-trifluoroethyl trifluoromethanesulfonate (33 μL, 0.23 mmol) were added to a solution of **12a** (36 mg, 0.19 mmol) in DMF (2 mL). The mixture was stirred at 90 °C for 12 h. After cooling, the mixture was extracted with EtOAc and washed with saturated brine. The combined organic layers were dried, concentrated, and purified using silica gel column chromatography (50% EtOAc in Pet. Ether), yielding **15** (25 mg, 49%) as colorless oil. ^1^H NMR (400 MHz, CDCl_3_) δ 7.64–7.42 (m, 5H), 3.73–3.46 (m, 1H), 2.95–2.72 (m, 3H), 2.70–2.47 (m, 4H), 2.07–1.76 (m, 3H), 1.62–1.41 (m, 1H). ^19^F NMR (376 MHz, CDCl_3_) δ −67.42. ^13^C NMR (101 MHz, CDCl_3_) δ 206.57, 131.70, 130.45, 130.04, 122.75 (d, *J* = 279.4 Hz), 74.23, 45.22 (q, *J* = 36.3, 35.5 Hz), 40.32, 36.19, 27.43, 21.79. ESI-MS: *m*/*z* = 272.2 (M + H)^+^. HRMS *m*/*z*: calcd for C_14_H_17_F_3_NO 272.1257; found, 272.1264.

#### 3.1.13. *N*-(2-Oxo-1-phenylcyclohexyl)acetamide [35] (**16**)

To a solution of **12a** (36 mg, 0.19 mmol) in DCM, we added TEA (80 μL) and acetic anhydride (27 μL, 0.19 mmol). The mixture was stirred at rt for 2h and then concentrated and purified using prep-HPLC and lyophilized to obtain **16** (25 mg, 56.8%) as colorless oil. ^1^H NMR (400 MHz, CDCl_3_) δ 7.45–7.29 (m, 5H), 3.96–3.79 (m, 1H), 2.83 (s, 1H), 2.45–2.26 (m, 2H), 2.07–1.97 (m, 1H), 1.95–1.69 (m, 7H). ^13^C NMR (101 MHz, CDCl_3_) δ 208.66, 200.02, 137.32, 128.69(2C), 127.92, 127.54(2C), 77.23, 38.50, 35.57, 28.41, 24.07, 22.38. ESI-MS: *m*/*z* = 232.1 (M + H)^+^. HRMS *m*/*z*: calcd for C_14_H_17_NNaO_2_ 254.1151; found, 254.1159.

#### 3.1.14. 2,2,2-Trifluoro-*N*-(2-oxo-1-phenylcyclohexyl)acetamide (**17**)

Compound **17** was synthesized from **12a** and trifluoroacetic anhydride in a process similar to that described for the preparation of **16**. ^1^H NMR (400 MHz, CDCl_3_) δ 8.30 (s, 1H), 7.45–7.30 (m, 5H), 3.90–3.81 (m, 1H), 2.51–2.32 (m, 2H), 2.11–2.01 (m, 1H), 1.94–1.70 (m, 4H). ^19^F NMR (376 MHz, CDCl_3_) δ -76.14. ESI-MS: *m*/*z* = 286.1 (M + H)^+^. HRMS *m*/*z*: calcd for C_14_H_14_F_3_NNaO_2_ 308.0869; found, 308.0875.

#### 3.1.15. 2-(Methylamino)-2-phenylcyclohexan-1-one [34] (**18**)

To a solution of **12a** (86 mg, 0.45 mmol) in THF (3 mL), we added di-tert-butyl dicarbonate (149 mg, 0.44 mmol) and TEA (0.1 mL, 0.9 mmol). The mixture was refluxed at 70 °C for 3 h and then concentrated and purified using silica gel column chromatography (10% EtOAc in Pet. Ether), yielding theyellow oil (92 mg, 70.7%). Then, the compound was dissolved in THF, and LiAlH_4_ (15 mg, 0.39 mmol) was added. The mixture was refluxed for 1 h, quenched using sodium hydroxide solution (10%), and extracted with EtOAc. The combined organic layers were concentrated under reduced pressure and dissolved in acetone (3 mL); then, Jones reagent (1 mL) was added. The mixture was stirred for 1 h at rt, and the pH was rendered basic using hydroxide solution (10%). The mixture was extracted with EtOAc and concentrated under reduced pressure and then purified using prep-HPLC to obtain **18** (55 mg, 69.6%) as white solid. ^1^H NMR (400 MHz, CDCl_3_) δ 7.39 (t, *J* = 7.6 Hz, 2H), 7.34–7.23 (m, 3H), 2.92 (dd, *J* = 14.1, 3.3 Hz, 1H), 2.70 (s, 1H), 2.45–2.39 (m, 1H), 2.37–2.28 (m, 1H), 2.07 (s, 3H), 2.00–1.80 (m, 3H), 1.80–1.67 (m, 2H). ^13^C NMR (101 MHz, CDCl_3_) δ 210.69, 137.58, 129.00, 127.92, 127.32, 70.15, 39.75, 34.96, 28.71, 27.71, 22.20. ESI-MS: *m*/*z* = 204.2 (M + H)^+^. HRMS *m*/*z*: calcd for C_13_H_18_NO 204.1383; found, 204.1389.

#### 3.1.16. 2-Amino-2-phenylcycloheptanone (**19**)

Compound **19** was synthesized in a process similar to that described for the preparation of **12a**. The result was a white solid. ^1^H NMR (400 MHz, CDCl_3_) δ 7.38–7.31 (m, 4H), 7.30–7.24 (m, 1H), 2.71–2.63 (m, 1H), 2.43–2.22 (m, 3H), 2.10 (s, 2H), 2.00–1.86 (m, 3H), 1.60–1.36 (m, 3H). ^13^C NMR (101 MHz, CDCl_3_) δ 214.25, 144.19, 128.91(2C), 127.55, 125.73(2C), 67.90, 40.52, 37.05, 30.49, 27.30, 24.01. ESI-MS: *m*/*z* = 204.2 (M + H)^+^. HRMS *m*/*z*: calcd for C_13_H_18_NO 204.1383; found, 204.1388.

#### 3.1.17. 2-(Ethylamino)-2-phenylcycloheptan-1-one (**20**)

Compound **20** was synthesized in a process similar to that described for the preparation of **13**. The result was a white solid. ^1^H NMR (400 MHz, CDCl_3_) δ 7.37 (t, *J* = 7.5 Hz, 2H), 7.31–7.21 (m, 3H), 2.94–2.85 (m, 1H), 2.44–2.37 (m, 1H), 2.36–2.24 (m, 2H), 2.14 (s, 1H), 2.08–2.00 (m, 1H), 1.98–1.92 (m, 1H), 1.89–1.69 (m, 4H), 0.99 (t, *J* = 7.1 Hz, 3H). ^13^C NMR (101 MHz, CDCl_3_) δ 211.89, 140.08, 128.63 (2C), 127.52, 127.20 (2C), 72.12, 40.01, 36.83, 31.30, 30.34, 27.21, 23.55, 15.49. ESI-MS: *m*/*z* = 232.2 (M + H)^+^. HRMS *m*/*z*: calcd for C_15_H_22_NO 232.1696; found, 232.1704.

#### 3.1.18. 2-(Methylamino)-2-phenylcycloheptan-1-one (**21**)

Compound **21** was synthesized in a process similar to that described for the preparation of **18**. The result was a yellow oil. ^1^H NMR (400 MHz, CDCl_3_) δ 7.39–7.31 (m, 2H), 7.30–7.23 (m, 3H), 2.58–2.49 (m, 1H), 2.38–2.24 (m, 2H), 2.20–2.08 (m, 2H), 1.96 (s, 3H), 1.93–1.81 (m, 3H), 1.59–1.39 (m, 3H). ^13^C NMR (101 MHz, CDCl_3_) δ 212.16, 139.71, 128.63 (2C), 127.46, 127.25 (2C), 71.99, 40.01, 30.44, 30.18, 28.82, 27.40, 23.61. ESI-MS: *m*/*z* = 218.2 (M + H)^+^. HRMS *m*/*z*: calcd for C_14_H_20_NO 218.1539; found, 218.1545.

#### 3.1.19. 2-Amino-2-(2-chlorophenyl)cycloheptanone (**22**)

Compound **22** was synthesized in a process similar to that described for the preparation of **12a**. The result was a yellow oil. ^1^H NMR (400 MHz, CDCl_3_) δ 7.50 (d, *J* = 7.7 Hz, 1H), 7.37 (d, *J* = 7.7 Hz, 1H), 7.26 (dt, *J* = 23.1, 7.3 Hz, 2H), 2.75–2.61 (m, 2H), 2.47–2.35 (m, 1H), 2.10 (s, 2H), 1.97–1.85 (m, 1H), 1.82–1.54 (m, 6H). ^13^C NMR (101 MHz, CDCl_3_) δ 214.13, 142.09, 132.90, 131.29, 128.76, 127.98, 127.15, 68.38, 40.02, 38.26, 28.34, 23.82, 23.71. ESI-MS: *m*/*z* = 238.1 (M + H)^+^. HRMS *m*/*z*: calcd for C_13_H_17_ClNO 238.0993; found, 238.0995.

#### 3.1.20. 2-(2-Chlorophenyl)-2-(ethylamino)cycloheptan-1-one hydrochloride (**23**)

Compound **23** was synthesized in a process similar to that described for the preparation of **13**. The result was a white solid. ^1^H NMR (400 MHz, CDCl_3_) δ 10.74 (s, 1H), 8.80 (s, 1H), 8.17 (d, *J* = 7.7 Hz, 1H), 7.55–7.47 (m, 1H), 7.44–7.35 (m, 2H), 3.51–3.28 (m, 2H), 2.85–2.76 (m, 1H), 2.70–2.60 (m, 1H), 2.58–2.45 (m, 2H), 1.87–1.77 (m, 4H), 1.76–1.71 (m, 1H), 1.48 (t, *J* = 7.1 Hz, 3H), 1.41–1.29 (m, 1H). ^13^C NMR (101 MHz, CDCl_3_) δ 204.67, 133.48, 133.17, 132.17, 131.49, 130.88, 128.74, 75.02, 40.63, 40.11, 36.87, 29.61, 25.63, 23.57, 12.27. ESI-MS: *m*/*z* = 266.1 (M + H)^+^. HRMS *m*/*z*: calcd for C_15_H_21_ClNO 266.1306; found, 266.1315.

#### 3.1.21. 2-(2-Chlorophenyl)-2-(methylamino)cycloheptan-1-one (**24**)

Compound **24** was synthesized in a process similar to that described for the preparation of **18**. The result was a yellow oil. ^1^H NMR (400 MHz, CDCl_3_) δ 7.45 (dd, *J* = 7.8, 1.7 Hz, 1H), 7.36 (dd, *J* = 7.7, 1.6 Hz, 1H), 7.32–7.20 (m, 2H), 2.75–2.66 (m, 1H), 2.49 (ddd, *J* = 12.4, 10.5, 5.5 Hz, 1H), 2.32 (s, 1H), 2.21–2.11 (m, 2H), 2.02 (s, 3H), 1.89–1.66 (m, 4H), 1.64–1.54 (m, 1H), 1.50–1.37 (m, 1H). ^13^C NMR (101 MHz, CDCl_3_) δ 211.37, 138.69, 133.51, 131.25, 129.93, 128.66, 126.57, 73.01, 39.43, 34.00, 29.25, 28.35, 24.06, 23.73. ESI-MS: *m*/*z* = 252.1 (M + H)^+^. HRMS *m*/*z*: calcd for C_14_H_19_ClNO 252.1150; found, 252.1152.

#### 3.1.22. 2-Amino-2-(naphthalen-1-yl)cycloheptan-1-one (**25**)

Compound **25** was synthesized in a process similar to that described for the preparation of **12a**. The result was a yellow oil. ^1^H NMR (400 MHz, CDCl_3_) δ 8.19 (dd, *J* = 6.4, 3.4 Hz, 1H), 7.87 (dd, *J* = 6.3, 3.3 Hz, 1H), 7.81 (d, *J* = 8.2 Hz, 1H), 7.57 (d, *J* = 7.3 Hz, 1H), 7.50–7.41 (m, 3H), 2.67–2.58 (m, 1H), 2.58–2.49 (m, 1H), 2.37–2.28 (m, 1H), 2.23–2.08 (m, 3H), 1.95–1.82 (m, 1H), 1.74–1.51 (m, 5H). ^13^C NMR (101 MHz, CDCl_3_) δ 216.15, 138.51, 134.96, 131.36, 129.38, 129.03, 126.37, 125.58, 125.05, 125.03, 124.49, 69.22, 40.55, 39.29, 28.52, 24.44, 23.62. ESI-MS: *m*/*z* = 254.2 (M + H)^+^. HRMS *m*/*z*: calcd for C_17_H_20_NO 254.1539; found, 254.1546.

#### 3.1.23. 2-(Ethylamino)-2-(naphthalen-1-yl)cycloheptan-1-one (**26**)

Compound **26** was synthesized in a process similar to that described for the preparation of **13**. The result was a yellow oil. ^1^H NMR (400 MHz, CDCl_3_) δ 8.39 (d, *J* = 9.5 Hz, 1H), 7.88–7.78 (m, 2H), 7.60 (d, *J* = 7.4 Hz, 1H), 7.49–7.41 (m, 3H), 2.64 (dd, *J* = 14.2, 6.9 Hz, 1H), 2.45–2.32 (m, 2H), 2.22–2.10 (m, 2H), 1.98–1.89 (m, 1H), 1.84–1.75 (m, 1H), 1.74–1.52 (m, 6H), 0.92 (t, *J* = 7.1 Hz, 3H). ^13^C NMR (101 MHz, CDCl_3_) δ 213.41, 134.49, 132.25, 130.12, 129.03, 128.93, 126.41, 126.12, 125.56, 125.39, 124.72, 73.30, 38.89, 37.01, 35.85, 28.99, 25.12, 23.31, 15.89. ESI-MS: *m*/*z* = 282.2 (M + H)^+^. HRMS *m*/*z*: calcd for C_19_H_24_NO 282.1852; found, 282.1860.

#### 3.1.24. 2-(Methylamino)-2-(naphthalen-1-yl)cycloheptan-1-one (**27**)

Compound **27** was synthesized in a process similar to that described for the preparation of **14**. The result was a yellow oil. ^1^H NMR (400 MHz, CDCl_3_) δ 8.34–8.25 (m, 1H), 7.89–7.78 (m, 2H), 7.62 (d, *J* = 7.3 Hz, 1H), 7.53–7.40 (m, 3H), 2.64 (dd, *J* = 14.5, 6.5 Hz, 1H), 2.40 (td, *J* = 11.5, 3.9 Hz, 1H), 2.20–2.13 (m, 1H), 2.08 (dd, *J* = 14.1, 10.4 Hz, 1H), 1.99 (s, 3H), 1.94 (s, 1H), 1.86–1.77 (m, 1H), 1.72–1.53 (m, 5H). ^13^C NMR (101 MHz, CDCl_3_) δ 213.56, 135.08, 134.53, 132.24, 129.11, 129.01, 126.65, 126.29, 125.57, 125.11, 124.74, 73.32, 38.87, 34.87, 29.15, 28.98, 25.05, 23.25. ESI-MS: *m*/*z* = 268.2 (M + H)^+^. HRMS *m*/*z*: calcd for C_18_H_22_NO 268.1696; found, 268.1698.

#### 3.1.25. 2-(Dimethylamino)-2-(naphthalen-1-yl)cycloheptan-1-one (**28**)

Compound **28** was synthesized in a process similar to that described for the preparation of **14**. The result was a yellow oil. ^1^H NMR (400 MHz, CDCl_3_) δ 9.01 (s, 1H), 7.81 (dd, *J* = 6.4, 3.3 Hz, 1H), 7.76 (d, *J* = 8.0 Hz, 1H), 7.45 (dt, *J* = 6.5, 3.3 Hz, 2H), 7.38 (t, *J* = 7.7 Hz, 1H), 7.34–7.29 (m, 1H), 2.61 (dd, *J* = 14.3, 10.2 Hz, 1H), 2.48 (d, *J* = 10.0 Hz, 2H), 2.36 (s, 7H), 1.82–1.72 (m, 1H), 1.69–1.56 (m, 2H), 1.55–1.40 (m, 3H). ^13^C NMR (101 MHz, CDCl_3_) δ 211.71, 137.36, 134.76, 132.46, 128.74 (2C), 127.80, 127.16, 125.50, 125.43, 124.42, 78.04, 44.00, 40.35 (2C), 32.85, 30.93, 25.76 (2C). ESI-MS: *m*/*z* = 282.2 (M + H)^+^. HRMS *m*/*z*: calcd for C_19_H_24_NO 282.1852; found, 282.1858.

#### 3.1.26. 2-Phenyl-3,4-dihydronaphthalen-1(2H)-one [36] (**29a**)

Sodium tert-butoxide (1 g, 10.3 mmol) was added to a solution of 3,4-dihydronaphthalen-1(2H)-one (1 g, 6.85 mmol) in THF (30 mL). The mixture was stirred at 70 °C for 0.5 h before iodobenzene (0.92 mL, 8.22 mmol), Pd(dba)_2_ (79 mg, 0.14 mmol), and DtBPF (81 mg, 0.17 mmol) were added. The mixture was stirred at this temperature for 12h and then poured into water (50 mL) and extracted with ethyl acetate (40 mL*3). The combined organic layers were concentrated under reduced pressure and purified using silica gel column chromatography (3% EtOAc in Pet. Ether) to obtain **29a** as white solid. ^1^H NMR (400 MHz, CDCl_3_) δ 8.11 (dd, *J* = 7.8, 1.2 Hz, 1H), 7.51 (td, *J* = 7.5, 1.4 Hz, 1H), 7.38–7.32 (m, 3H), 7.31–7.26 (m, 2H), 7.22–7.18 (m, 2H), 3.85–3.77 (m, 1H), 3.17–3.03 (m, 2H), 2.49–2.40 (m, 2H). ESI-MS: *m*/*z* = 223.20 (M + H)^+^.

#### 3.1.27. 2-Bromo-2-phenyl-3,4-dihydronaphthalen-1(2H)-one (**32a**)

Compound **32a** was synthesized in a process similar to that described for the preparation of **4a**. The compound was a yellow solid. ^1^H NMR (400 MHz, CDCl_3_) δ 8.20 (d, *J* = 7.9 Hz, 1H), 7.56 (d, *J* = 7.5 Hz, 2H), 7.50 (t, *J* = 7.4 Hz, 1H), 7.40–7.28 (m, 4H), 7.26–7.20 (m, 1H), 3.26–3.17 (m, 1H), 3.01–2.90 (m, 2H), 2.90–2.82 (m, 1H). ^13^C NMR (101 MHz, CDCl_3_) δ 190.75, 142.63, 139.03, 133.91, 130.70, 129.24, 128.62, 128.49, 128.40 (2C), 127.98 (2C), 127.18, 69.71, 40.37, 28.02.

#### 3.1.28. 2-Azido-2-phenyl-3,4-dihydronaphthalen-1(2H)-one (**33a**)

To a solution of 32a (410 mg, 1.36 mmol) in DMSO (10 mL), we added sodium azide (93 mg, 1.43 mmol). The mixture was stirred at rt for 12h and then poured into water (10 mL) and extracted with ethyl acetate (10 mL*3). The combined organic layers were concentrated under reduced pressure and purified using silica gel column chromatography (5% EtOAc in Pet. Ether) to obtain **33a** as white solid. ^1^H NMR (400 MHz, CDCl_3_) δ 8.21 (dd, *J* = 7.9, 1.1 Hz, 1H), 7.50 (td, *J* = 7.5, 1.3 Hz, 1H), 7.42–7.29 (m, 6H), 7.17 (d, *J* = 7.6 Hz, 1H), 2.92 (dt, *J* = 17.2, 4.1 Hz, 1H), 2.78–2.67 (m, 1H), 2.61 (dt, *J* = 13.7, 4.0 Hz, 1H), 2.38 (ddd, *J* = 13.7, 11.9, 4.5 Hz, 1H).^13^C NMR (101 MHz, CDCl_3_) δ 196.22, 143.26, 135.90, 134.18, 131.86, 129.03(2C), 128.89, 128.79, 128.15, 127.16, 127.08(2C), 71.60, 35.42, 26.14.

#### 3.1.29. 2-Amino-2-phenyl-3,4-dihydronaphthalen-1(2H)-one (**31a**)

We added 5% palladium/C catalyst (30 mg) to a solution of **33a** (300 mg, 1.14 mmol) in MeOH (10 mL). The mixture was stirred in a hydrogen atmosphere for 2h. The mixture was filtrated using celite, and the filtrate was concentrated and purified using silica gel column chromatography, yielding **31a** (200 mg, 74%) as yellow oil. ^1^H NMR (400 MHz, CDCl_3_) δ 8.18 (dd, *J* = 7.8, 1.2 Hz, 1H), 7.46 (td, *J* = 7.5, 1.5 Hz, 1H), 7.38–7.32 (m, 1H), 7.32–7.21 (m, 5H), 7.15 (d, *J* = 7.6 Hz, 1H), 2.92–2.82 (m, 1H), 2.80–2.64 (m, 2H), 2.35–2.24 (m, 1H), 2.14 (s, 2H). ^13^C NMR (101 MHz, CDCl_3_) δ 201.59, 143.77, 141.42, 133.61, 132.35, 128.81, 128.65(2C), 127.85, 127.72, 126.83, 126.43(2C), 63.44, 37.22, 26.62. ESI-MS: *m*/*z* = 238.20 (M + H)^+^.

#### 3.1.30. 2-(Dimethylamino)-2-phenyl-3,4-dihydronaphthalen-1(2H)-one hydrochloride (**34**)

Compound **34** was synthesized in a process similar to that described for the preparation of **14**. The result was a white solid. ^1^H NMR (400 MHz, CDCl_3_) δ 8.14 (d, *J* = 7.8 Hz, 1H), 7.41 (t, *J* = 7.3 Hz, 1H), 7.35–7.25 (m, 6H), 7.08 (d, *J* = 7.6 Hz, 1H), 2.89–2.82 (m, 1H), 2.77–2.54 (m, 3H), 2.47 (s, 6H). ^13^C NMR (101 MHz, CDCl_3_) δ 196.37, 142.62, 134.28, 133.57, 132.80, 129.44, 129.18 (2C), 128.69, 128.65 (2C), 127.90, 127.14, 73.59, 40.27 (2C), 31.75, 26.91. ESI-MS: *m*/*z* = 266.25 (M + H)^+^. HRMS *m*/*z*: calcd for C_18_H_20_NO 266.1539; found, 266.1542.

#### 3.1.31. 2-(Dimethylamino)-6-methoxy-2-phenyl-3,4-dihydronaphthalen-1(2H)-one (**35**)

Compound **35** was synthesized in a process similar to that described for the preparation of **14**. The result was a white solid. ^1^H NMR (400 MHz, CDCl_3_) δ 8.11 (d, *J* = 8.8 Hz, 1H), 7.35–7.23 (m, 5H), 6.83 (dd, *J* = 8.8, 2.4 Hz, 1H), 6.52 (d, *J* = 2.2 Hz, 1H), 3.80 (s, 3H), 2.85–2.47 (m, 4H), 2.43 (s, 6H). ^13^C NMR (101 MHz, CDCl_3_) δ 198.93, 163.49, 145.47, 138.13, 130.03, 128.35 (2C), 127.81, 127.67, 113.43, 112.01, 71.39, 55.39, 48.93, 40.44, 32.06, 27.70. ESI-MS: *m*/*z* = 296.25 (M + H)^+^. HRMS *m*/*z*: calcd for C_19_H_22_NO_2_ 296.1645; found, 296.1649.

#### 3.1.32. 2-(2-Chlorophenyl)-2-(dimethylamino)-3,4-dihydronaphthalen-1(2H)-one (**36**)

Compound **36** was synthesized in a process similar to that described for the preparation of **14**. The result was a white solid. ^1^H NMR (400 MHz, CDCl_3_) δ 8.11 (d, *J* = 7.9 Hz, 1H), 7.42 (td, *J* = 7.5, 1.5 Hz, 1H), 7.33 (q, *J* = 7.6 Hz, 2H), 7.21–7.10 (m, 4H), 3.17 (ddd, *J* = 14.1, 6.0, 4.3 Hz, 1H), 2.97 (dt, *J* = 17.1, 5.1 Hz, 1H), 2.66 (ddd, *J* = 17.0, 9.5, 4.2 Hz, 1H), 2.59 (s, 6H), 2.45 (ddd, *J* = 13.9, 9.4, 4.4 Hz, 1H). ^13^C NMR (101 MHz, CDCl_3_) δ 197.84, 142.82, 137.47, 134.07, 134.02, 132.81, 131.99, 130.96, 128.64, 128.30, 128.12, 126.63, 126.32, 71.99, 40.19 (2C), 32.71, 27.20.ESI-MS: *m*/*z* = 300.2 (M + H)^+^. HRMS *m*/*z*: calcd for C_18_H_19_ClNO 300.1150; found, 300.1146.

#### 3.1.33. 2-(2-Chlorophenyl)-2-(dimethylamino)-2,3-dihydro-1H-inden-1-one Hydrochloride (**37**)

Compound **37** was synthesized in a process similar to that described for the preparation of **14**. The result was a white solid. ^1^H NMR (400 MHz, CDCl_3_) δ 12.41 (s, 1H), 7.96 (d, *J* = 7.7 Hz, 1H), 7.88 (m, 1H), 7.77 (t, *J* = 7.3 Hz, 1H), 7.59–7.47 (m, 3H), 7.44–7.34 (m, 2H), 4.45 (d, *J* = 19.2 Hz, 1H), 3.91 (d, *J* = 19.0 Hz, 1H), 2.90 (d, *J* = 60.9 Hz, 6H). ^13^C NMR (101 MHz, CDCl_3_) δ 198.87, 150.41, 137.75, 135.53, 132.45, 132.07, 131.20, 131.11, 129.22, 127.69, 126.53, 124.98, 74.78, 41.89, 40.66, 40.05. ESI-MS: *m*/*z* = 286.20 (M + H)^+^. HRMS *m*/*z*: calcd for C_17_H_17_ClNO 286.0993; found, 286.0998.

#### 3.1.34. 6-(Dimethylamino)-6-phenyl-6,7,8,9-tetrahydro-5H-benzo[7]annulen-5-one Hydrochloride (**38**)

Compound **38** was synthesized in a process similar to that described for the preparation of **14**. The result was a white solid. ^1^H NMR (400 MHz, CDCl_3_) δ 12.28 (s, 1H), 7.90 (d, *J* = 7.6 Hz, 1H), 7.59–7.47 (m, 4H), 7.43 (t, *J* = 7.5 Hz, 1H), 7.26 (s, 2H), 7.15 (d, *J* = 7.5 Hz, 1H), 3.51–3.42 (m, 1H), 2.83 (d, *J* = 3.6 Hz, 3H), 2.72 (d, *J* = 4.1 Hz, 3H), 2.50–2.26 (m, 3H), 1.92–1.80 (m, 1H), 1.73 (s, 1H). ^13^C NMR (101 MHz, CDCl_3_) δ 202.21, 139.67, 137.49, 134.68, 130.56, 130.48, 130.16 (2C), 129.80 (4C), 127.57, 77.24, 41.88, 39.71, 30.73, 29.22, 22.17. ESI-MS: *m*/*z* = 280.2 (M + H)^+^. HRMS *m*/*z*: calcd for C_19_H_22_NO 280.1696; found, 280.1692.

#### 3.1.35. 2-(Methylamino)-2-phenyl-3,4-dihydronaphthalen-1(2H)-one (**41**)

TEA (87 μL, 0.63 mmol) and di-tert-butyl decarbonate (70 mg, 0.32 mmol) were added to a solution of **31a** (30 mg, 0.13 mmol) in DCM (3 mL). The mixture was stirred at rt for 10 h and directly concentrated and purified using silica gel column chromatography to attain **39a** (40 mg, 91%) as yellow oil. Then, **39a** (40 mg, 0.12) was dissolved in THF (3 mL), and NaH (10 mg, 0.24 mmol) and MeI (15 μL, 0.24 mmol) were added. The mixture was stirred at rt for 1h and then quenched with water and extracted with EtOAc. The combined organic layers were concentrated and dissolved in DCM (2 mL). Then, HCl/dioxane solution (1 mL, 4 M) was added, and the mixture was stirred at rt for 1 h. Sodium hydroxide solution (2.5 N) was added to change the pH so that it was basic. The mixture was extracted with EtOAc, and the combined organic was concentrated and then purified using HPLC and acidified to attain **41** (10 mg, 30%) as white solid. ^1^H NMR (400 MHz, CDCl_3_) δ 10.29 (d, *J* = 22.7 Hz, 2H), 8.12 (d, *J* = 7.7 Hz, 1H), 7.61–7.52 (m, 2H), 7.46 (t, *J* = 7.1 Hz, 1H), 7.42–7.30 (m, 4H), 7.09 (d, *J* = 7.6 Hz, 1H), 3.39–3.27 (m, 1H), 3.11–2.92 (m, 2H), 2.86–2.74 (m, 1H), 2.61 (s, 3H). ^13^C NMR (101 MHz, CDCl_3_) δ 193.39, 142.23, 134.51, 131.44, 130.11, 129.79, 129.51 (2C), 128.69 (2C), 128.64, 128.35, 127.20, 69.48, 31.43, 29.49, 25.94. ESI-MS: *m*/*z* = 252.25 (M + H)^+^. HRMS *m*/*z*: calcd for C_17_H_18_NO 252.1383; found, 252.1385.

#### 3.1.36. 6-Methoxy-2-(methylamino)-2-phenyl-3,4-dihydronaphthalen-1(2H)-one (**42**)

Compound **42** was synthesized in a process similar to that described for the preparation of **41**. The result was a white solid. ^1^H NMR (400 MHz, CDCl_3_) δ 10.17 (d, *J* = 39.4 Hz, 2H), 8.10 (d, *J* = 8.8 Hz, 1H), 7.63–7.53 (m, 2H), 7.44–7.34 (m, 3H), 6.85 (dd, *J* = 8.8, 2.2 Hz, 1H), 6.53 (d, *J* = 2.0 Hz, 1H), 3.81 (s, 3H), 3.28 (d, *J* = 12.9 Hz, 1H), 3.07–2.86 (m, 2H), 2.81–2.68 (m, 1H), 2.60 (t, *J* = 5.2 Hz, 3H). ^13^C NMR (101 MHz, CDCl_3_) δ 191.90, 164.56, 144.92, 130.88, 130.24, 129.99, 129.44 (2C), 128.68 (2C), 124.85, 114.18, 112.27, 69.20, 55.54, 31.42, 29.58, 26.29. ESI-MS: *m*/*z* = 282.20 (M + H)^+^. HRMS *m*/*z*: calcd for C_18_H_20_NO_2_ 282.1489; found, 282.1494.

#### 3.1.37. 2-(2-Chlorophenyl)-2-(methylamino)-2,3-dihydro-1H-inden-1-one (**43**)

Compound **43** was synthesized in a process similar to that described for the preparation of **41**. The result was a white solid. ^1^H NMR (400 MHz, CDCl_3_) δ 11.34 (s, 1H), 10.10 (s, 1H), 7.90 (d, *J* = 7.6 Hz, 1H), 7.67 (t, *J* = 7.3 Hz, 1H), 7.59 (d, *J* = 7.2 Hz, 1H), 7.50–7.39 (m, 3H), 7.37–7.28 (m, 2H), 4.23 (d, *J* = 17.8 Hz, 1H), 3.86 (d, *J* = 17.6 Hz, 1H), 2.73 (s, 3H). ^13^C NMR (101 MHz, CDCl_3_) δ 198.84, 149.89, 136.85, 134.66, 133.12, 131.78, 131.69, 130.75, 130.38, 128.78, 127.54, 126.35, 125.23, 69.47, 40.69, 29.51. ESI-MS: *m*/*z* = 272.15 (M + H)^+^. HRMS *m*/*z*: calcd for C_16_H_15_ClNO 272.0837; found, 272.0845.

### 3.2. Biology

#### Electrophysiological Patch Clamp Experiment Conducted using HEK293 Cells

(1) A glass slice seeded with HEK293 cells was first placed in a recording tank and subsequently perfused with extracellular fluid at a rate of 4 mL/min. The composition of the extracellular fluid was as follows: NaCl, 140 mM; KCl, 2.8 mM; HEPES, 10 mM; CaCl_2_, 1 mM; glycine, 0.1 mM (pH 7.2). (2) An ALA-VC3 eight-channel pressure perfusion system was filled with extracellular fluid containing sodium glutamate (1 mM) and sodium glutamate plus extracellular fluid with measured compounds, respectively, with a drip rate of about 1 drop/sec, with 10 μL/drop. (3) Cells with moderate fluorescence intensity and good adhesion were then selected using fluorescence microscopy for testing. (4) The microtube of the perfusion system was placed approximately 10 cells away from the cells to be tested. The drug was sprayed on the surfaces of the cells under air pressure (about 0.02 MPa). (5) The glass electrode was drawn with a microelectrode drawing instrument with a resistance of 4–6 MΩ, and the electrode was filled with liquid (CsCl 125 mM, HEPES 10 mM, EGTA 11 mM). Subsequently, the glass electrode was pushed toward the cell with a microelectrode manipulator, and negative pressure was applied after the electrode was attached to the cell membrane so that a high-resistance seal of more than 1 GΩ formed between the electrode tip and the cell membrane, and then negative pressure was applied to suck the electrode through the cell membrane to facilitate a whole-cell recording mode. (6) After facilitating the whole-cell recording mode, the cell membrane potential was clamped at −70 mV, and the extracellular fluid containing 1 mM of sodium glutamate, 1 mM of monosodium glutamate, and the compound to be tested; the extracellular fluid containing 1 mM of monosodium glutamate; and the extracellular fluid were sprayed at 10 s, 20 s, 20 s, 20 s, and 20 s sequentially on the surface of the cell membrane to record the NMDA-receptor-mediated current and evaluate the compound to be tested for GluN2A-containing inhibition of NMDAR. The experimental process was controlled using pCLAMP 10.6, and a Digidata 1550A digital-to-analog converter was used to complete the generation of stimulus signals and the acquisition of feedback signals.

## 4. Conclusions

Depression is a chronic and debilitating mental illness with limited treatment options. Ketamine, an NMDAR antagonist, can rapidly relieve most symptoms of depression. However, its psychotomimetic side effects limit its application. Therefore, there is a high demand for potent and brain-penetrating NMDAR inhibitors to meet the unmet medical need. This study analyzed the cryo-EM structure of ketamine and MK-801 with NMDAR and developed a molecular design using an approximate conformational search. Initially, we attempted to make a complex structural modification to the aromatic region of ketamine. However, due to significant steric hindrance, no compounds with satisfactory activity were found. Subsequently, we modified the structure of the B fragment with nitrogen alkylation; unfortunately, there were no compounds with better activity than ketamine. Next, the aliphatic ring region was modified, leading to the discovery of seven-member ring compounds **23** and **24**, which exhibit excellent inhibitory activity against NMDAR. Additionally, benzo series compounds were designed and synthesized, which also demonstrate a certain inhibitory effect on NMDAR. These results indicate that this series of compounds has the potential to be used as leads for developing novel NMDAR inhibitors. Further experiments on PK/PD and toxicity will be conducted and verified in the future.

## Figures and Tables

**Table 1:** Ketamine derivatives **6**–**8** and **13**–**18**.

Compounds	Structure	Inhibitory Rate (%)	Compounds	Structure	Inhibitory Rate (%)
**6**	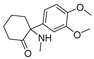	NA	**7**	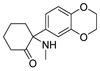	10
**8**	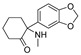	34	**13**	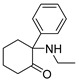	40
**14**	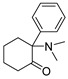	20	**15**	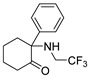	NA
**16**	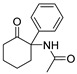	NA	**17**	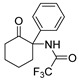	NA
**18**	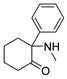	65	ket	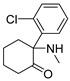	91

**Table 2:** Ketamine derivatives **19**–**28**.

Compounds	Structure	Inhibitory Rate (%)	Compounds	Structure	Inhibitory Rate (%)
**19**	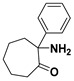	NA	**20**	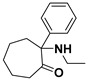	72
**21**	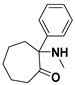	49	**22**	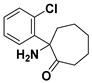	49
**23**	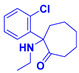	101	**24**	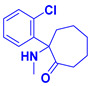	95
**25**	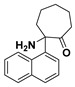	NA	**26**	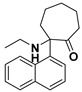	8
**27**	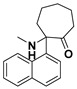	NA	**28**	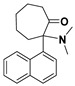	NA

**Table 3:** Ketamine derivatives **34**–**38** and **41**–**43**.

Compounds	Structure	Inhibitory Rate (%)	Compounds	Structure	Inhibitory Rate (%)
**34**	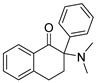	30	**35**	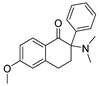	3
**36**	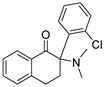	NA	**37**	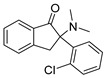	NA
**38**	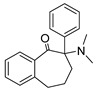	NA	**41**	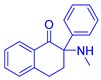	58
**42**	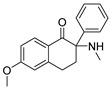	40	**43**	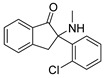	20

## Data Availability

The data are contained within the article and Supplementary Materials.

## Supplementary Materials

The following supporting information can be downloaded at https://www.mdpi.com/article/10.3390/molecules29112459/s1. ^1^H, ^13^C NMR Spectra of synthesized compounds.
