# Supplementary material for: Design, Synthesis and Biological Evaluation of Novel Ketamine Derivatives as NMDAR Antagonists
Source: Molecules. 2024 May 23;29(11):2459. doi: 10.3390/molecules29112459 (PMC11173549; doi:10.3390/molecules29112459)
Supplement: Supplementary file 1 [file molecules-29-02459-s001.zip › molecules-2986113-supplementary.pdf]

Design, synthesis and biological evaluation of novel ketamine derivatives as NMDAR antagonists

Shiyun Li [1,2,*], Bin Wen [1], Wei Zhao [3], Lulu Wang [3] and Xingquan Chen [1,*]

[1]Qingyuan Innovation Laboratory, Quanzhou 362801, China.

[2]Interdisciplinary Research Center on Biology and Chemistry, Shanghai Institute of Organic Chemistry, Chinese Academy of Sciences, Shanghai 201203, China

[3]College of Chemical Engineering, Fuzhou University, Fuzhou 350108, China

[*]Correspondence: lisy@sioc.ac.cn (S.L.); chenxqnxu@163.com (X.C.)

^{1}H NMR and ^{13}C NMR spectra of these compounds

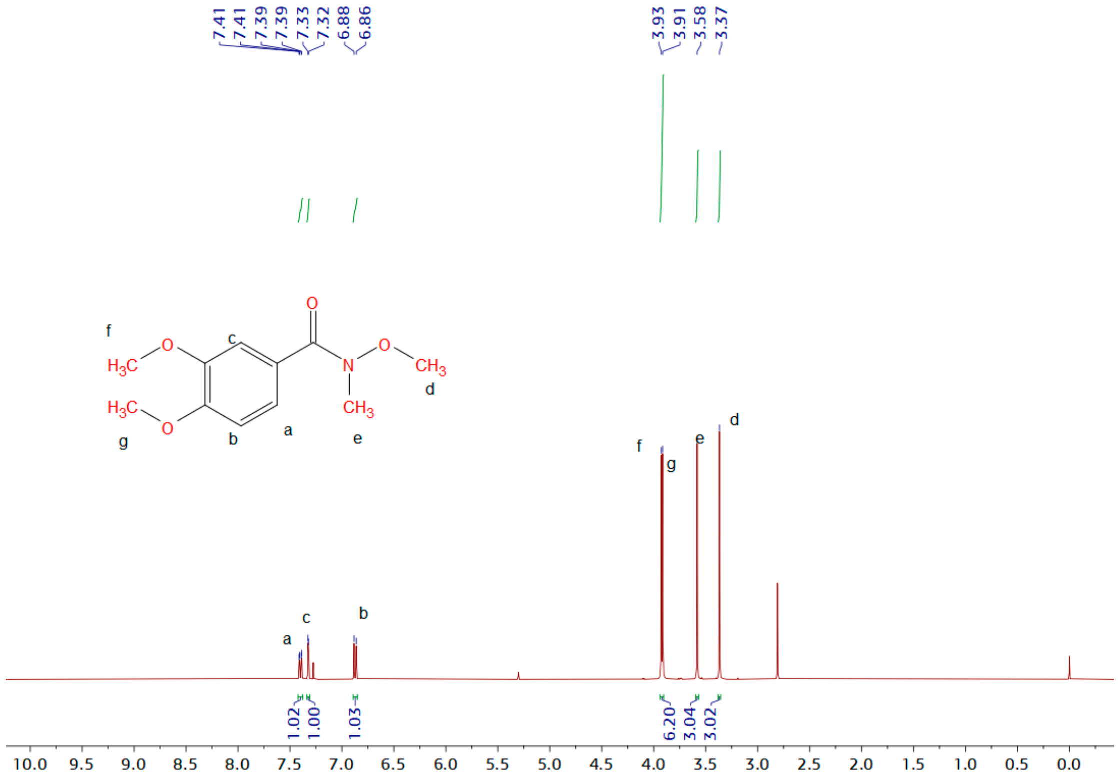

Figure S1 ^{1}H NMR spectrum of compound **2a** ($CDCl_3$, 400 MHz)

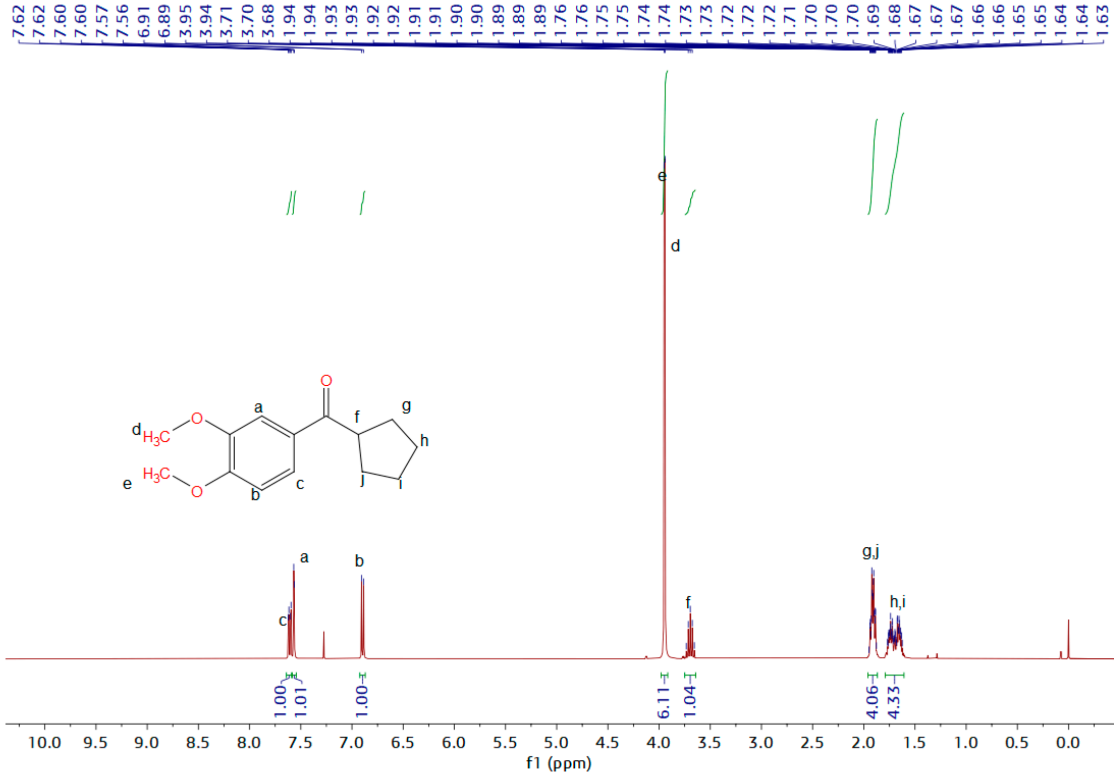

Figure S2 ^{1}H NMR spectrum of compound **3a** ($CDCl_3$, 400 MHz)

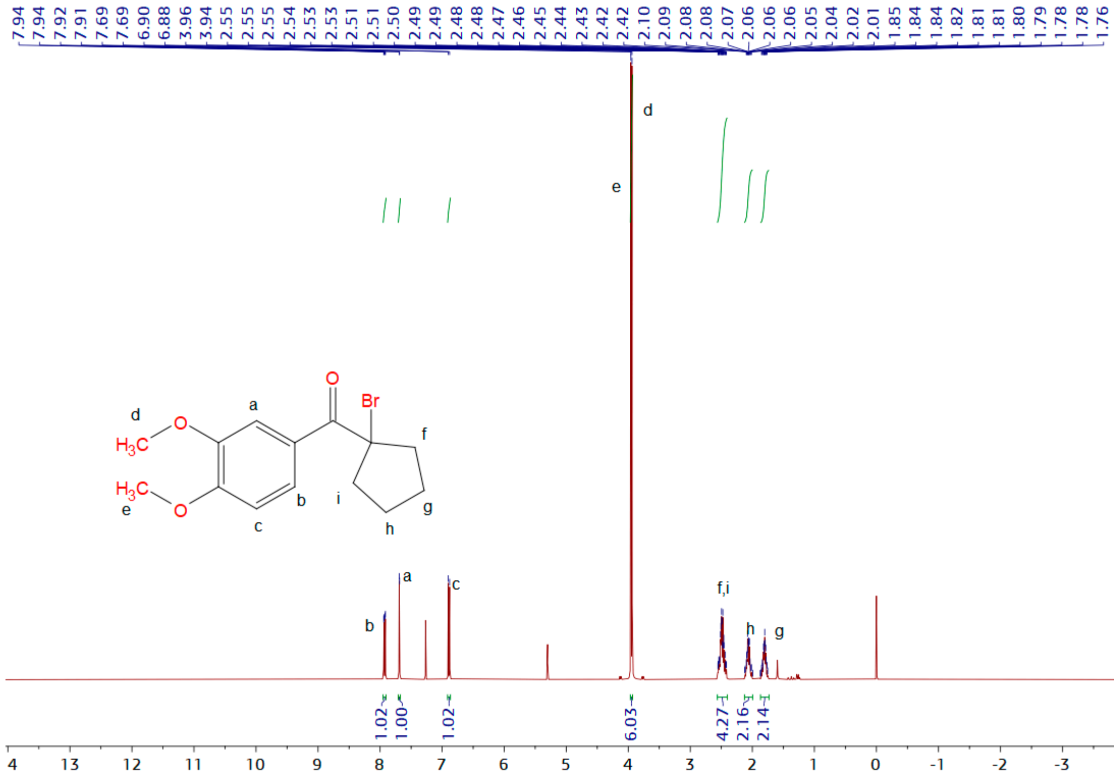

Figure S3 ^{1}H NMR spectrum of compound **4a** ($CDCl_3$, 400 MHz)

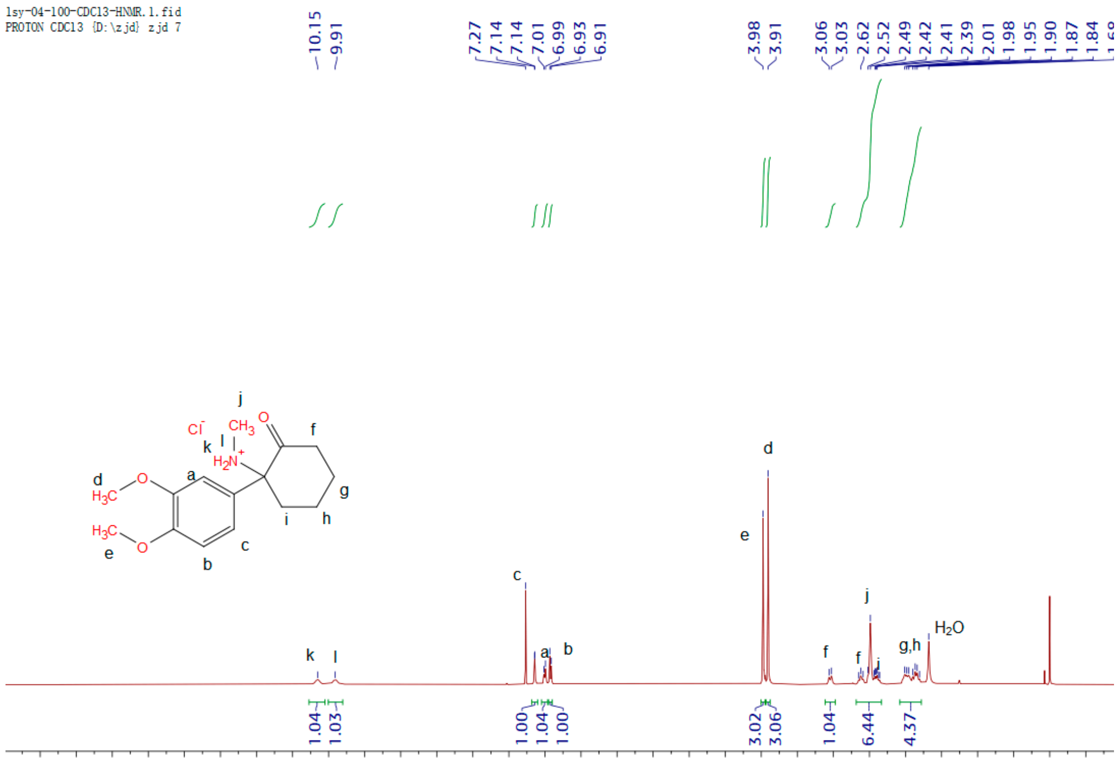

Figure S4 ^{1}H NMR spectrum of compound **6** ($CDCl_3$, 400 MHz)

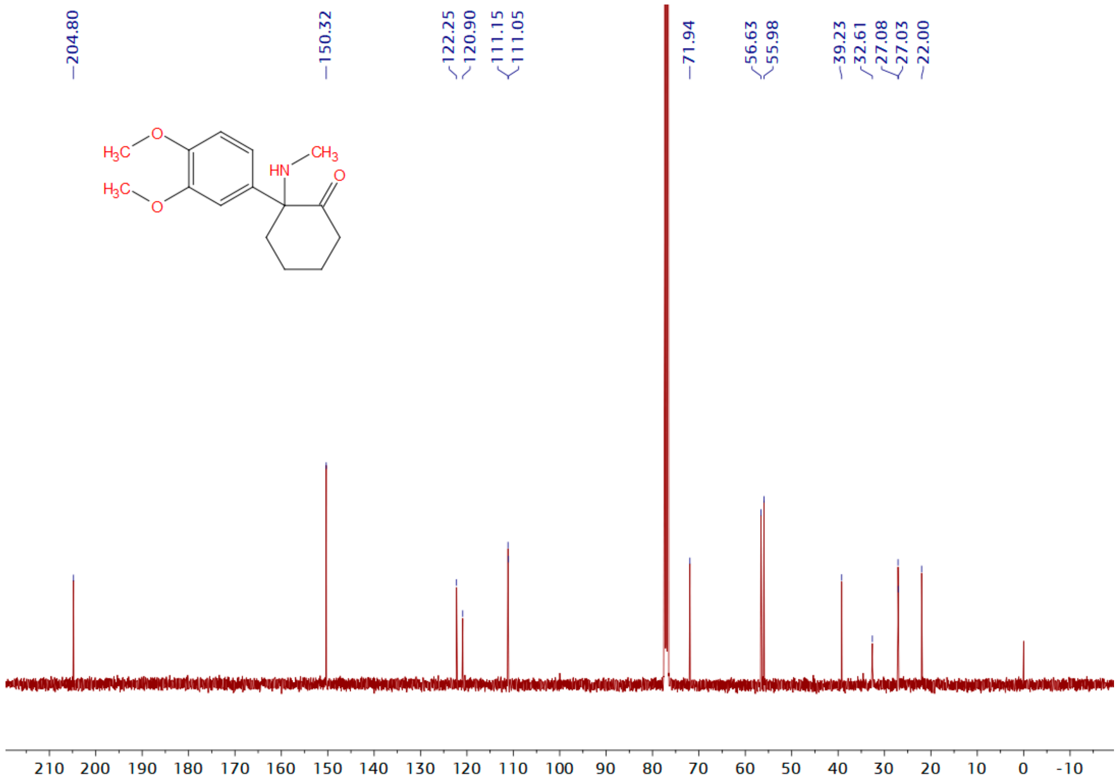

Figure S5 ^{13}C NMR spectrum of compound **6** ($CDCl_3$, 100 MHz)

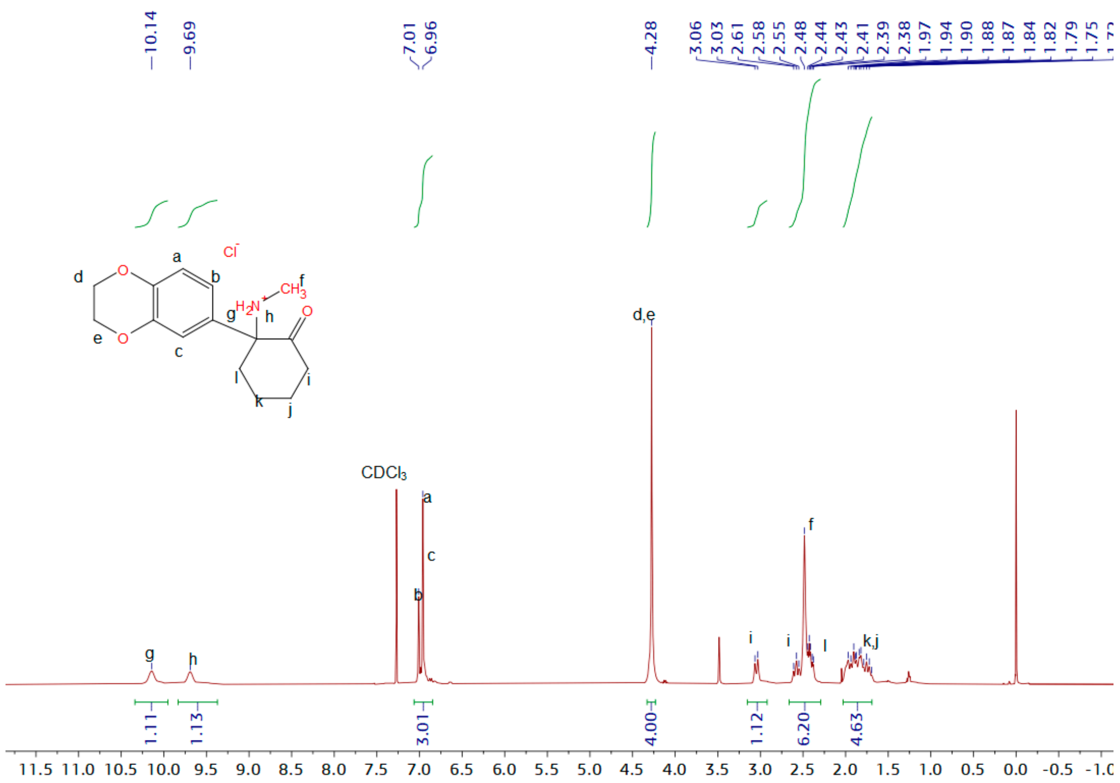

Figure S6 ^{1}H NMR spectrum of compound **7** ($CDCl_3$, 400 MHz)

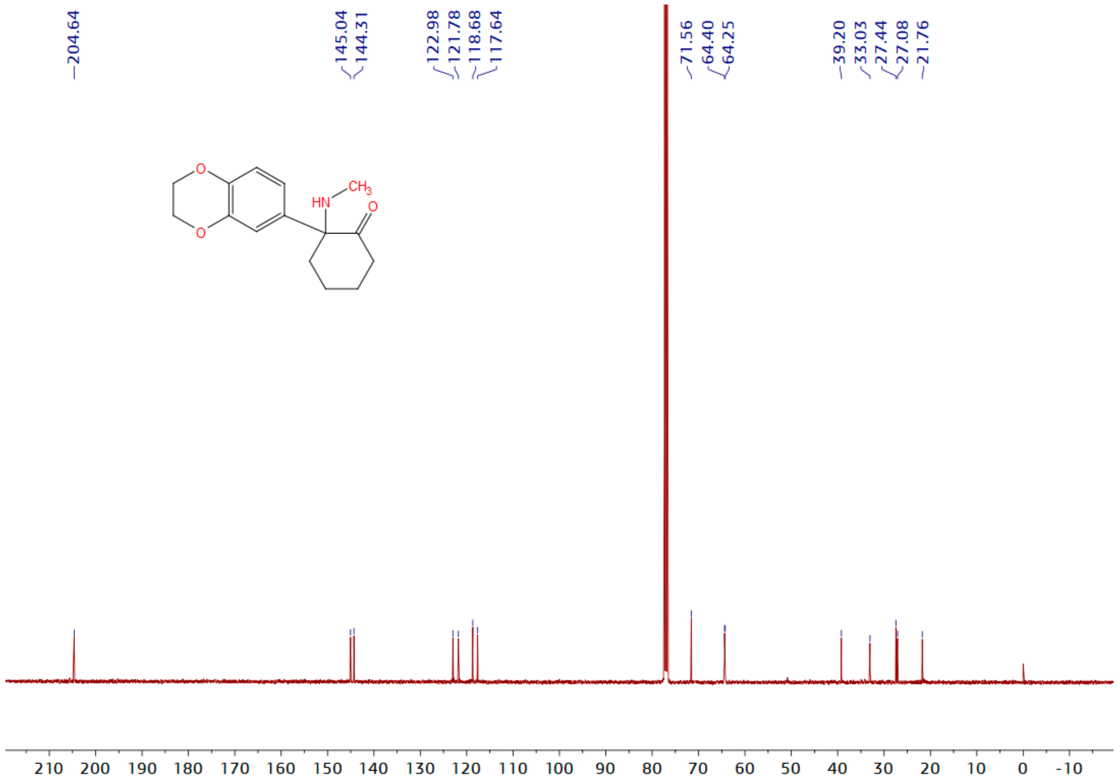

Figure S7 ^{13}C NMR spectrum of compound **7** ($CDCl_3$, 100 MHz)

Mass Spectrum SmartFormula Report

Analysis Info

Analysis Name	D:\Data\SHUJVFENXI\MADAWEIGROUP\2017195-LSY-5-14_RE1_01_31468.d	Acquisition Date	8/24/2022 2:36:09 AM
Method	20150915.m	Operator	BDAL@DE
Sample Name	2017195-LSY-5-14	Instrument / Ser#	maXis 4G 21240
Comment			

Acquisition Parameter

Source Type	ESI	Ion Polarity	Positive	Set Nebulizer	1.0 Bar
Focus	Not active	Set Capillary	4500 V	Set Dry Heater	220 °C
Scan Begin	50 m/z	Set End Plate Offset	-500 V	Set Dry Gas	6.0 l/min
Scan End	1500 m/z	Set Collision Cell RF	500.0 Vpp	Set Divert Valve	Waste

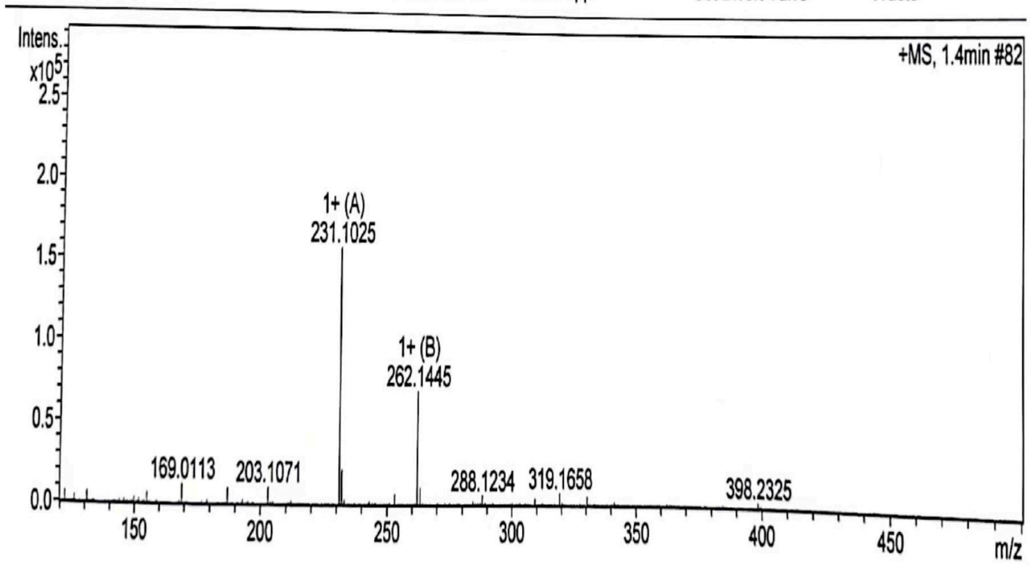

Meas. m/z	#	Formula	Score	m/z	err [ppm]	Mean err [ppm]	mSigma	rdb	e⁻ Conf	N-Rule
262.1445	1	C 15 H 20 N O 3	100.00	262.1438	-2.9	-2.8	9.2	6.5	even	ok

Figure S8 HRMS spectrum of compound **7**

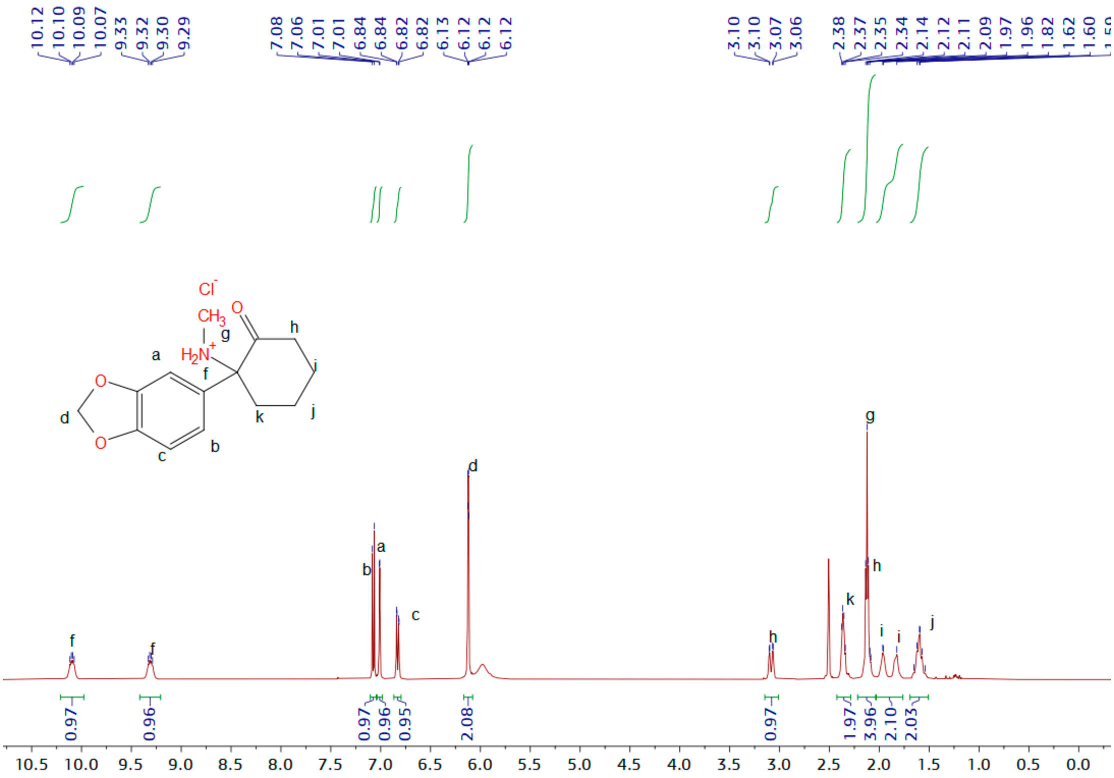

Figure S9 ^{1}H NMR spectrum of compound **8** ($CDCl_3$, 400 MHz)

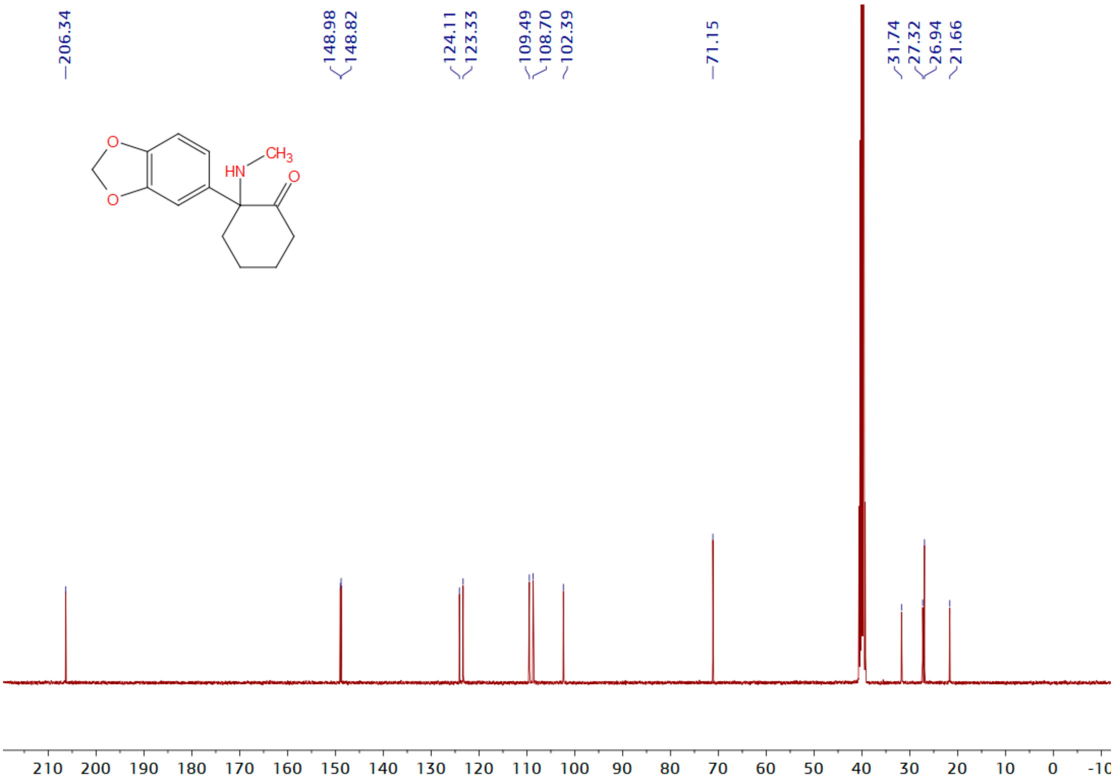

Figure S10 ^{13}C NMR spectrum of compound **8** ($CDCl_3$, 100 MHz)

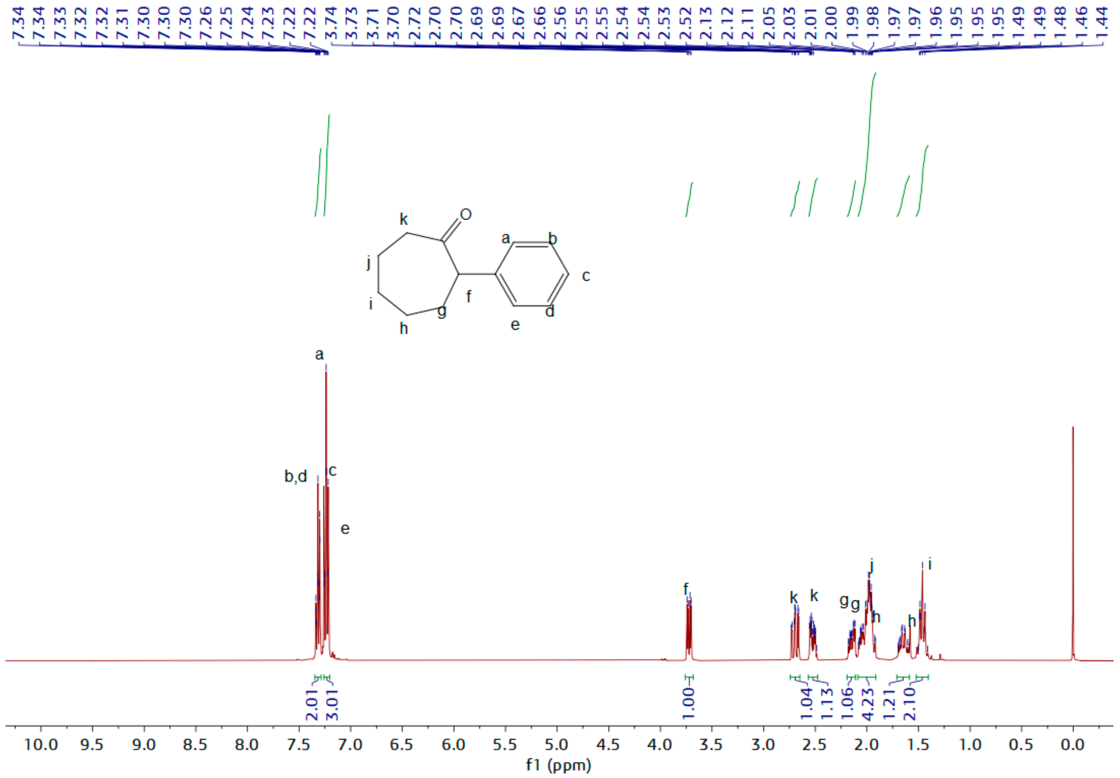

Figure S11 ^{1}H NMR spectrum of compound **10b** ($CDCl_3$, 400 MHz)

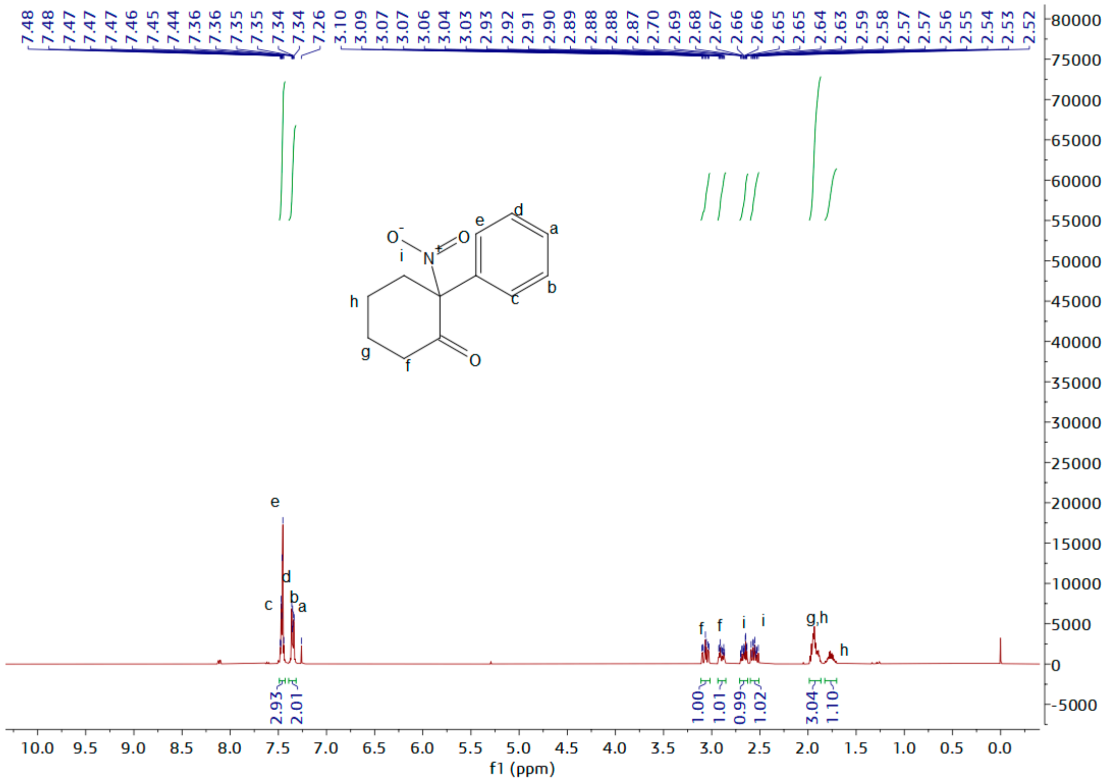

Figure S12 ^{1}H NMR spectrum of compound **11a** ($CDCl_3$, 400 MHz)

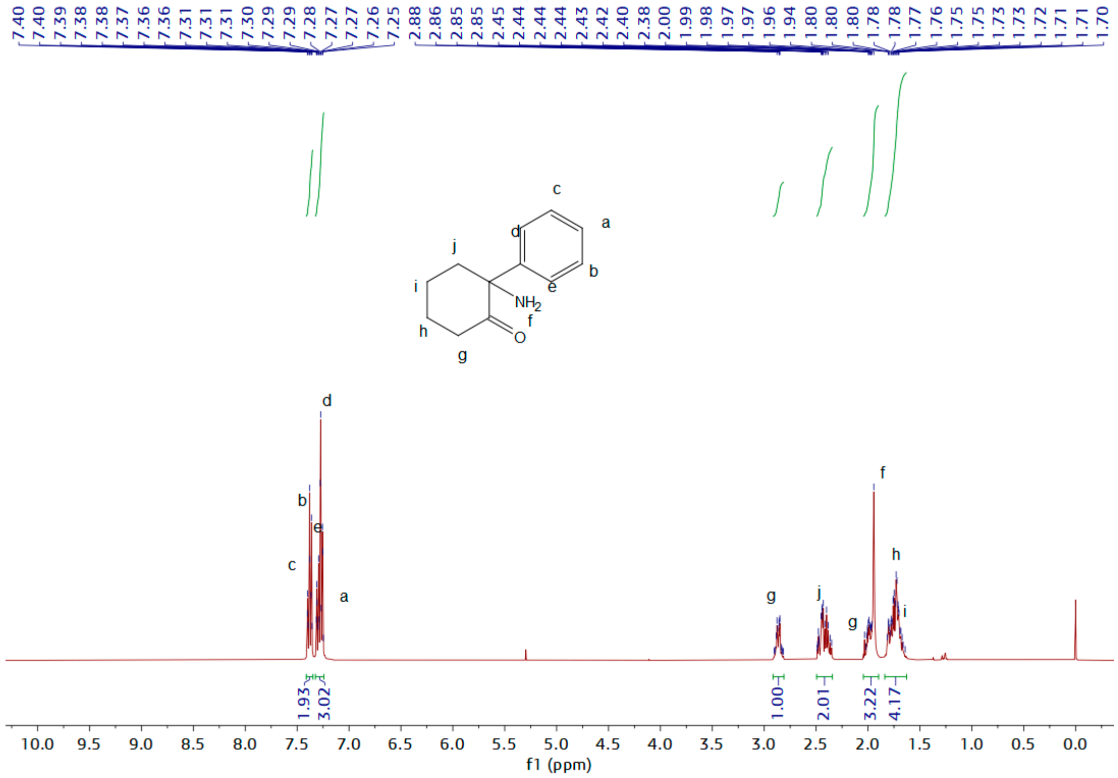

Figure S13 ^{1}H NMR spectrum of compound **12a** ($CDCl_3$, 400 MHz)

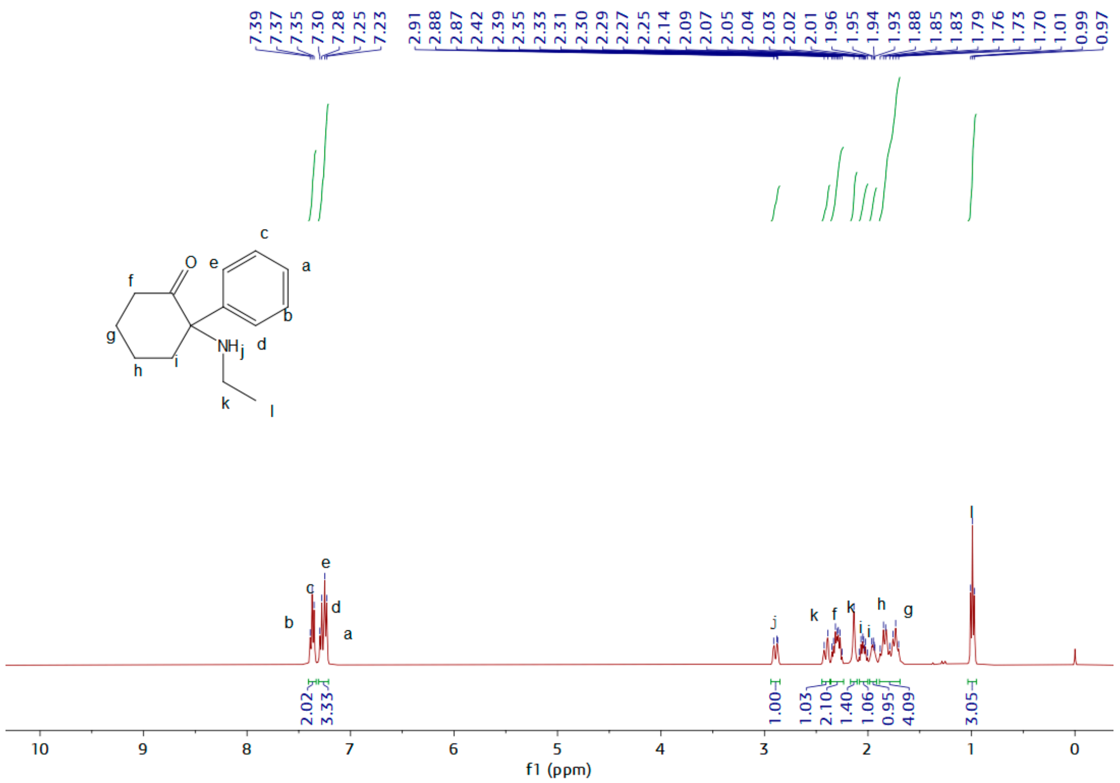

Figure S14 ^{1}H NMR spectrum of compound **13** ($CDCl_3$, 400 MHz)

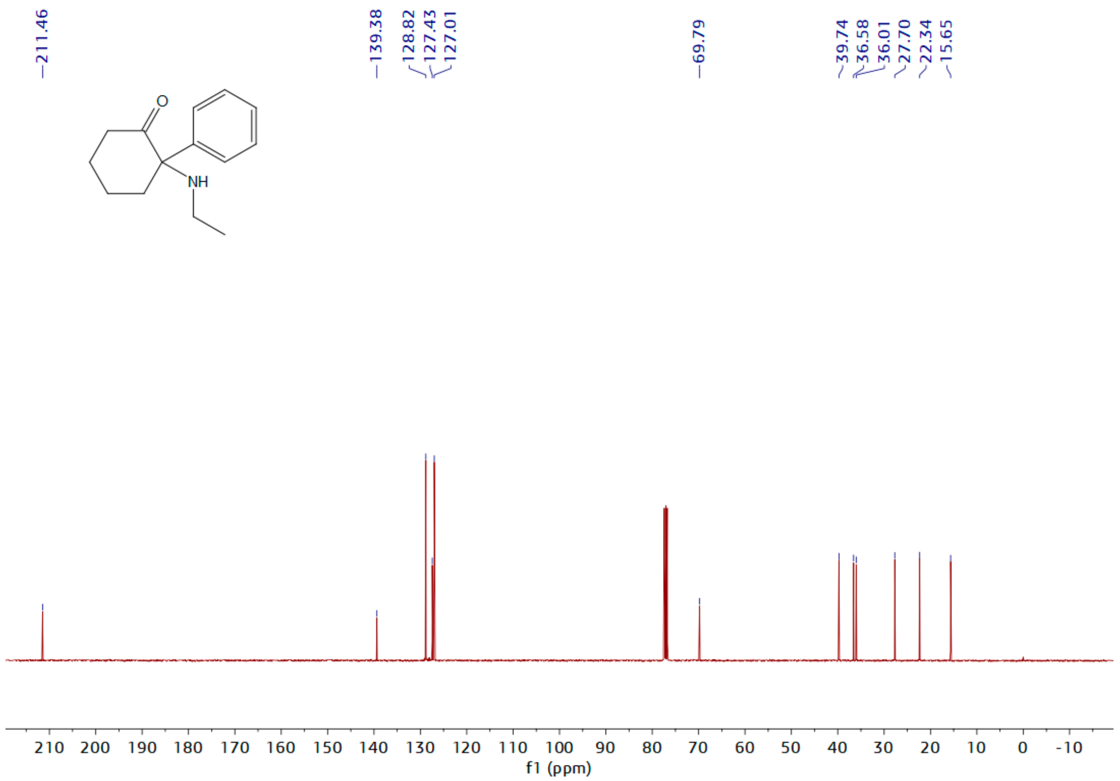

Figure S15 ^{13}C NMR spectrum of compound **13** ($CDCl_3$, 100 MHz)

Mass Spectrum SmartFormula Report

Analysis Info

Analysis Name D:\Data\SHUJVFENXI\MADAWEIGROUP\2017195-LSY-5-52_BD4_01_30890.d
Method 20150915.m
Sample Name 2017195-LSY-5-52
Comment

Acquisition Date 7/18/2022 8:39:14 AM
Operator BDAL@DE
Instrument / Ser# maXis 4G 21240

Acquisition Parameter

Source Type	ESI	Ion Polarity	Positive	Set Nebulizer	1.0 Bar
Focus	Not active	Set Capillary	4500 V	Set Dry Heater	220 °C
Scan Begin	50 m/z	Set End Plate Offset	-500 V	Set Dry Gas	6.0 l/min
Scan End	1500 m/z	Set Collision Cell RF	500.0 Vpp	Set Divert Valve	Waste

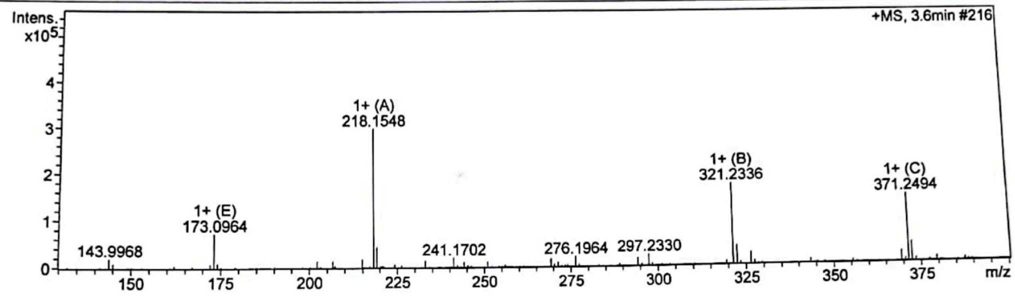

eas. m/z	#	Formula	Score	m/z	err [ppm]	Mean err [ppm]	mSigma	rdb	e⁻ Conf	N-Rule
218.1548	1	C 14 H 20 N O	100.00	218.1539	-4.1	-3.5	4.4	5.5	even	ok

Figure S16 HRMS spectrum of compound **13**

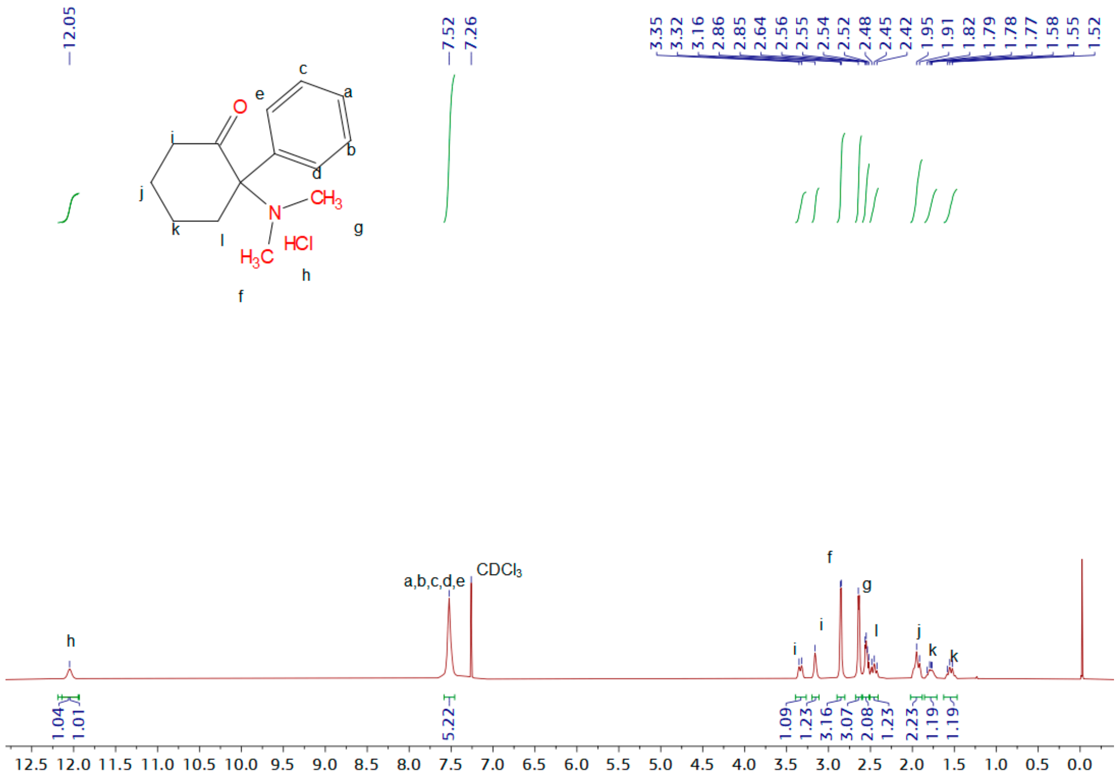

Figure S17 ^{1}H NMR spectrum of compound **14** ($CDCl_3$, 400 MHz)

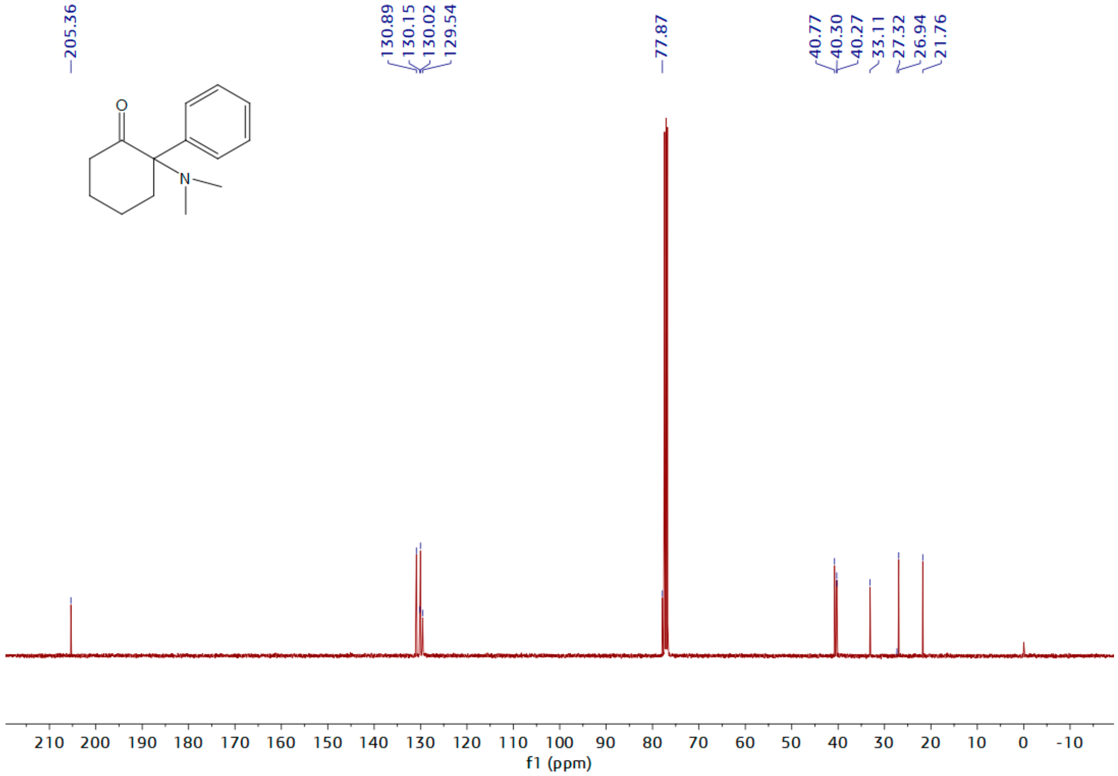

Figure S18 ^{13}C NMR spectrum of compound **14** ($CDCl_3$, 100 MHz)

Mass Spectrum SmartFormula Report

Analysis Info

Analysis Name	D:\Data\SHUJVFENXI\MADAWEIGROUP\2017195-LSY-5-82-1_RC6_01_31429.d	Acquisition Date	8/23/2022 7:38:22 AM
Method	20150915.m	Operator	BDAL@DE
Sample Name	2017195-LSY-5-82-1	Instrument / Ser#	maXis 4G 21240
Comment			

Acquisition Parameter

Source Type	ESI	Ion Polarity	Positive	Set Nebulizer	1.0 Bar
Focus	Not active	Set Capillary	4500 V	Set Dry Heater	220 °C
Scan Begin	50 m/z	Set End Plate Offset	-500 V	Set Dry Gas	6.0 l/min
Scan End	1500 m/z	Set Collision Cell RF	500.0 Vpp	Set Divert Valve	Waste

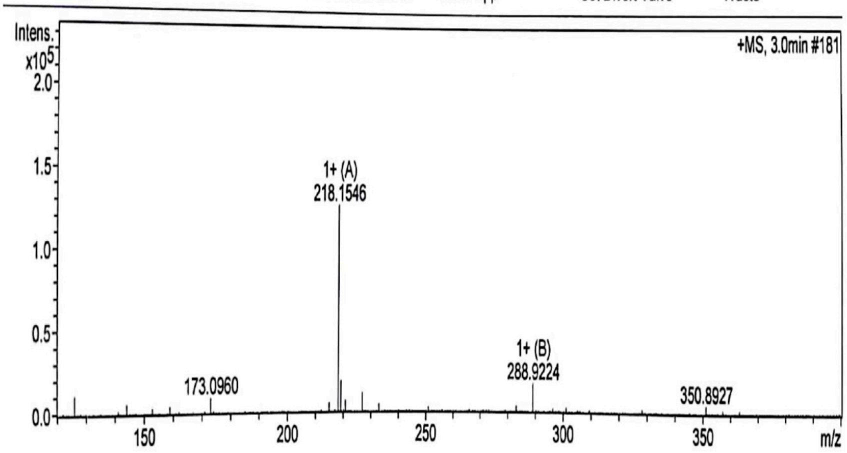

Meas. m/z	#	Formula	Score	m/z	err [ppm]	Mean err [ppm]	mSigma	rdb	e⁻ Conf	N-Rule
218.1546	1	C 14 H 20 N O	100.00	218.1539	-3.0	-2.6	3.4	5.5	even	ok

Figure S19 HRMS spectrum of compound **14**

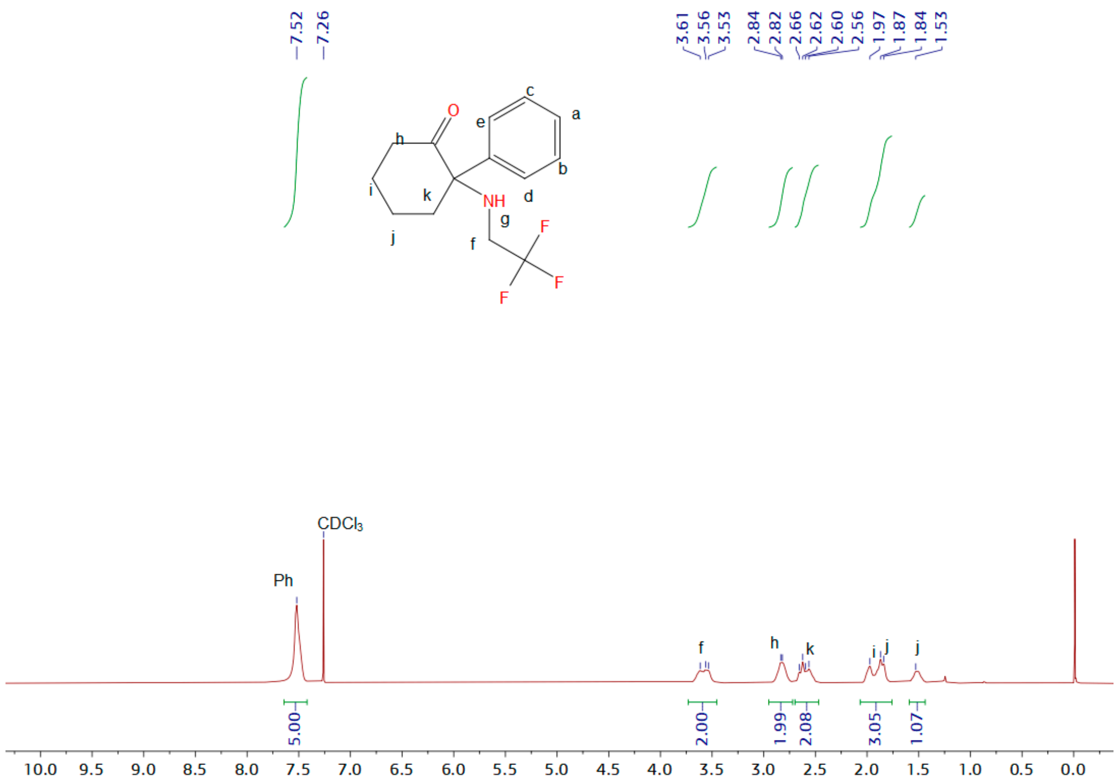

Figure S20 ^{1}H NMR spectrum of compound **15** ($CDCl_3$, 400 MHz)

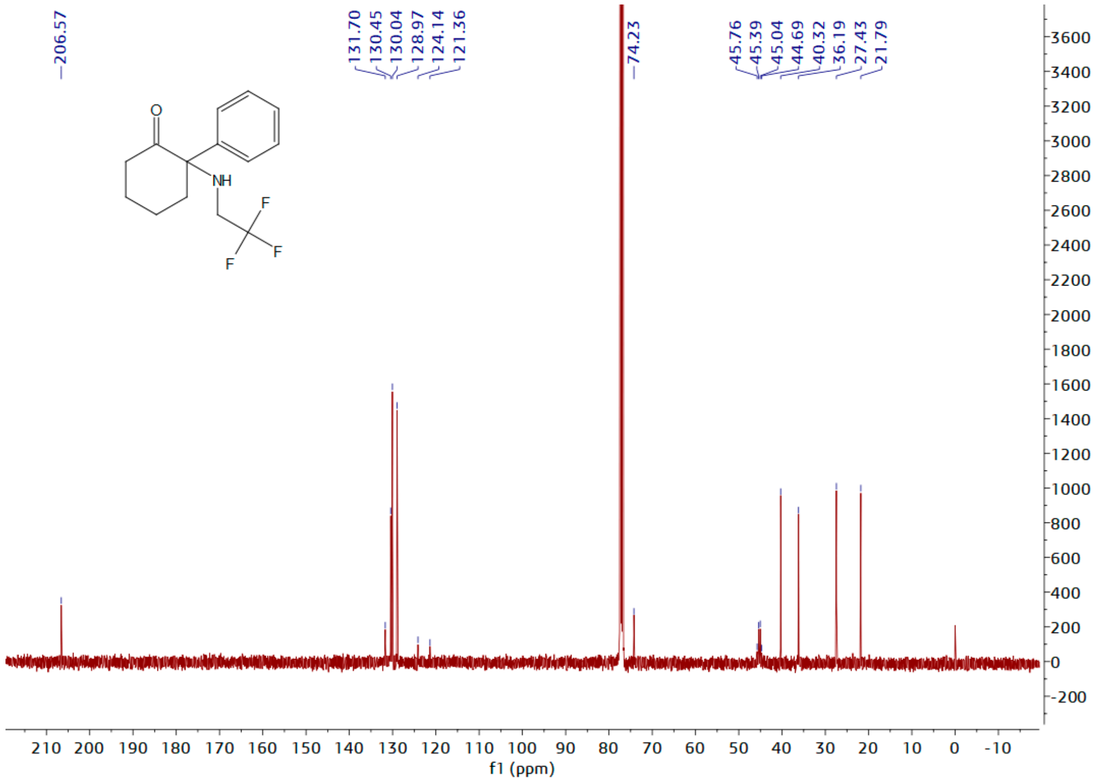

Figure S21 ^{13}C NMR spectrum of compound **15** ($CDCl_3$, 100 MHz)

Mass Spectrum SmartFormula Report

Analysis Info

Analysis Name	D:\Data\SHUJVFENXI\MADAWEIGROUP\2017195-LSY-6-18_RC7_01_31430.d	Acquisition Date	8/23/2022 7:44:55 AM
Method	20150915.m	Operator	BDAL@DE
Sample Name	2017195-LSY-6-18	Instrument / Ser#	maXis 4G 21240
Comment			

Acquisition Parameter

Source Type	ESI	Ion Polarity	Positive	Set Nebulizer	1.0 Bar
Focus	Not active	Set Capillary	4500 V	Set Dry Heater	220 °C
Scan Begin	50 m/z	Set End Plate Offset	-500 V	Set Dry Gas	6.0 l/min
Scan End	1500 m/z	Set Collision Cell RF	500.0 Vpp	Set Divert Valve	Waste

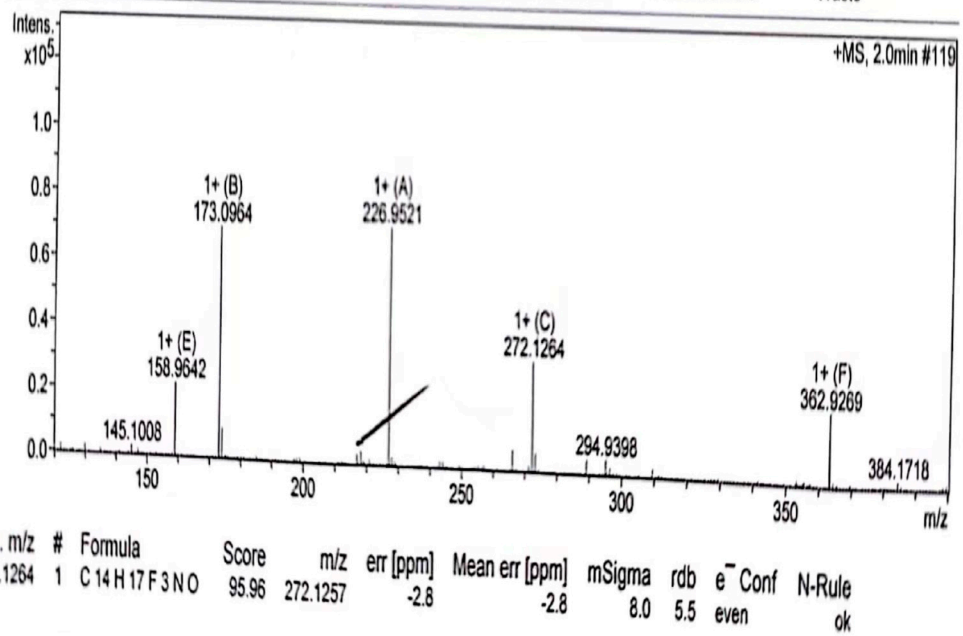

Meas. m/z	#	Formula	Score	m/z	err [ppm]	Mean err [ppm]	mSigma	rdb	e⁻ Conf	N-Rule
272.1264	1	C 14 H 17 F 3 N O	95.96	272.1257	-2.8	-2.8	8.0	5.5	even	ok

Figure S22 HRMS spectrum of compound **15**

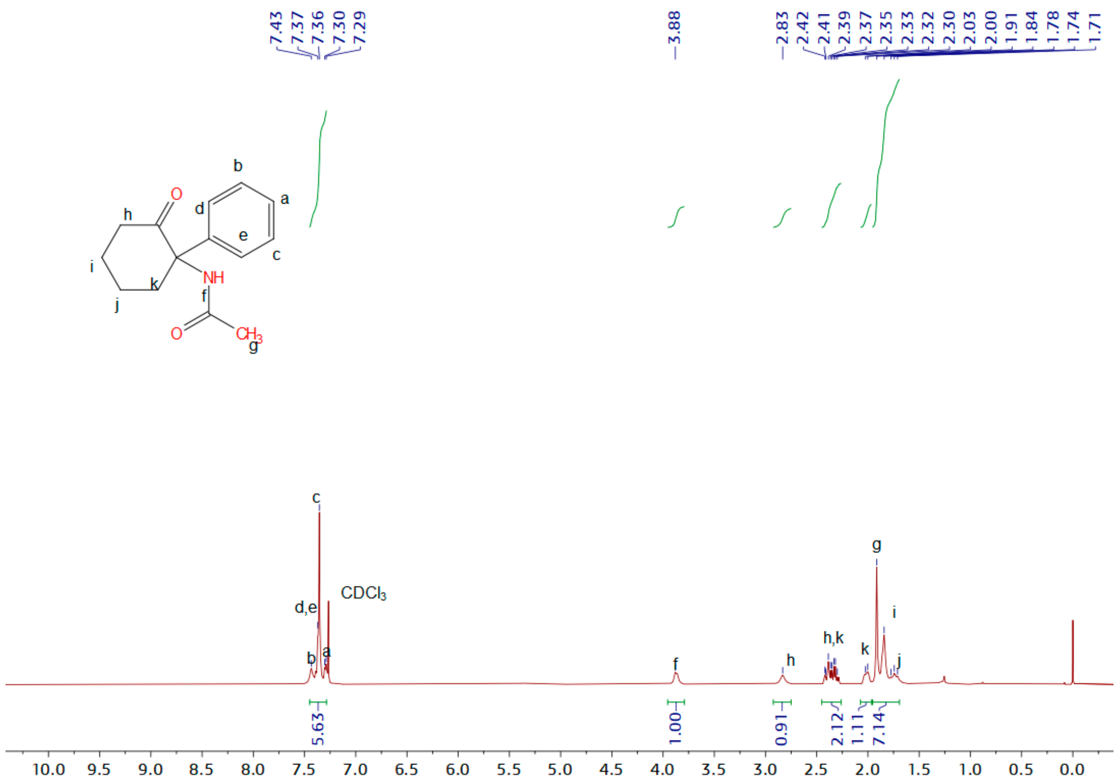

Figure S23 ^{1}H NMR spectrum of compound **16** ($CDCl_3$, 400 MHz)

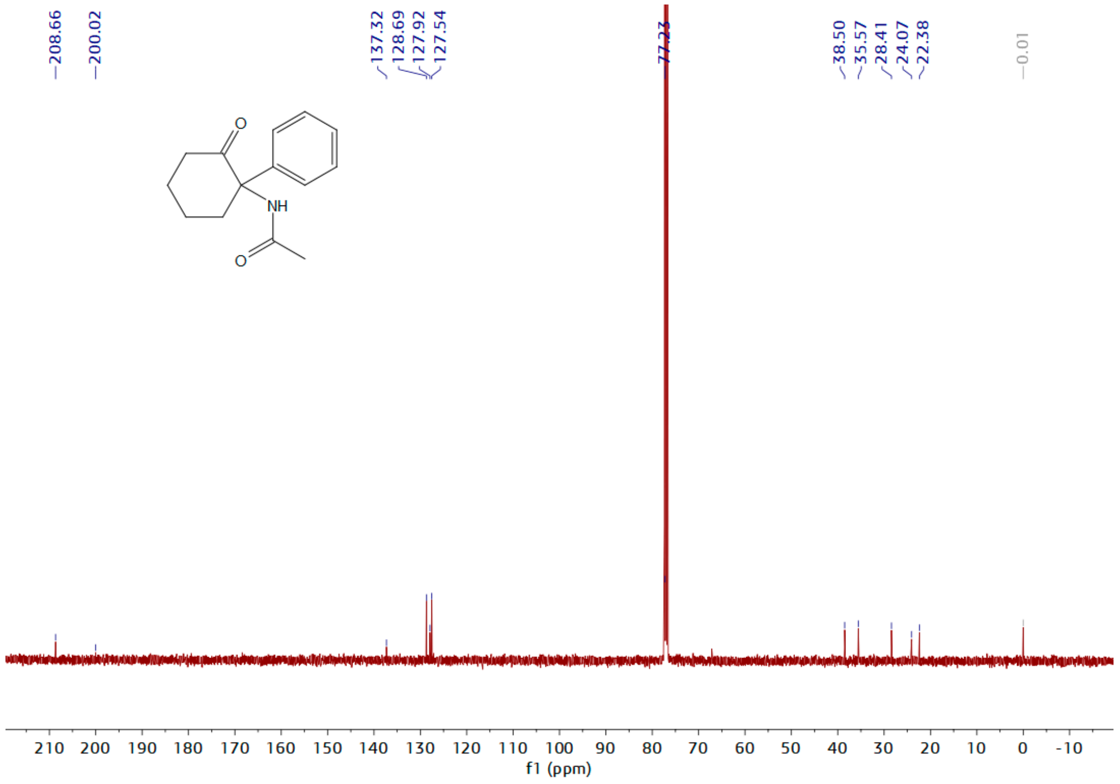

Figure S24 ^{13}C NMR spectrum of compound **16** ($CDCl_3$, 100 MHz)

Mass Spectrum SmartFormula Report

Analysis Info			
Analysis Name	D:\Data\SHUJVFENXI\MADAWEIGROUP\2017195-LSY-6-17_RB8_01_31423.d	Acquisition Date	8/23/2022 7:00:04 AM
Method	20150915.m	Operator	BDAL@DE
Sample Name	2017195-LSY-6-17	Instrument / Ser#	maXis 4G 21240
Comment			

Acquisition Parameter

Source Type	ESI	Ion Polarity	Positive	Set Nebulizer	1.0 Bar
Focus	Not active	Set Capillary	4500 V	Set Dry Heater	220 °C
Scan Begin	50 m/z	Set End Plate Offset	-500 V	Set Dry Gas	6.0 l/min
Scan End	1500 m/z	Set Collision Cell RF	500.0 Vpp	Set Divert Valve	Waste

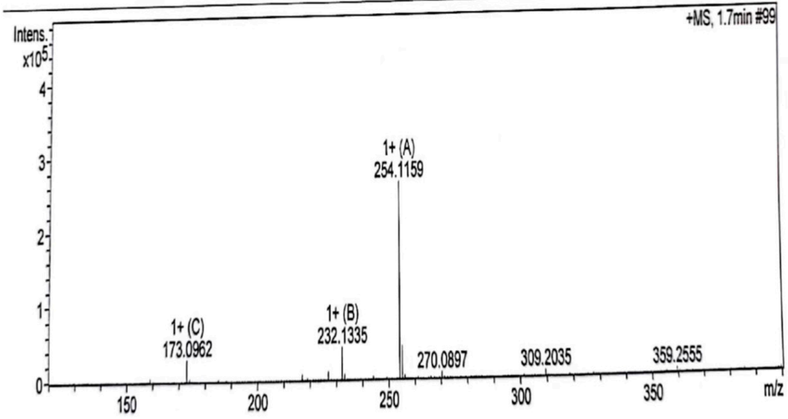

Meas. m/z	#	Formula	Score	m/z	err [ppm]	Mean err [ppm]	mSigma	rdb	e⁻ Conf	N-Rule
232.1335	1	C 14 H 18 N O 2	100.00	232.1332	-1.2	-1.2	1.3	6.5	even	ok
254.1159	1	C 14 H 17 N Na O 2	100.00	254.1151	-3.0	-2.7	4.9	6.5	even	ok

Figure S25 HRMS spectrum of compound **16**

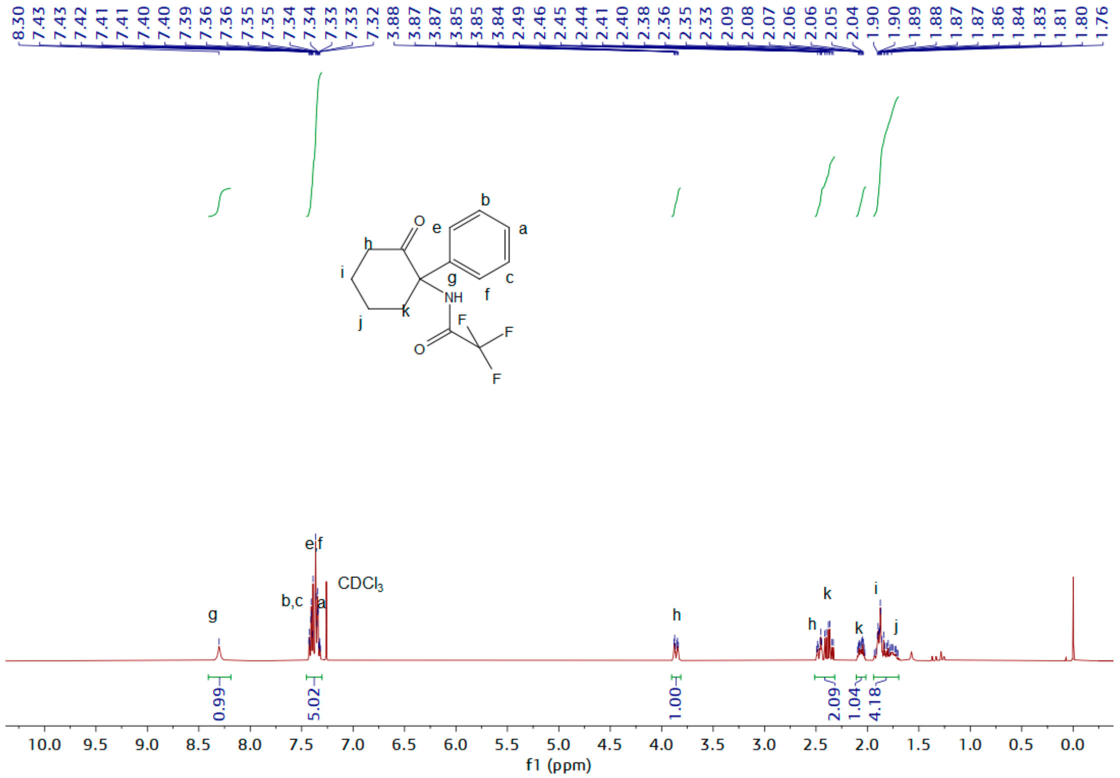

Figure S26 ^{1}H NMR spectrum of compound **17** ($CDCl_3$, 400 MHz)

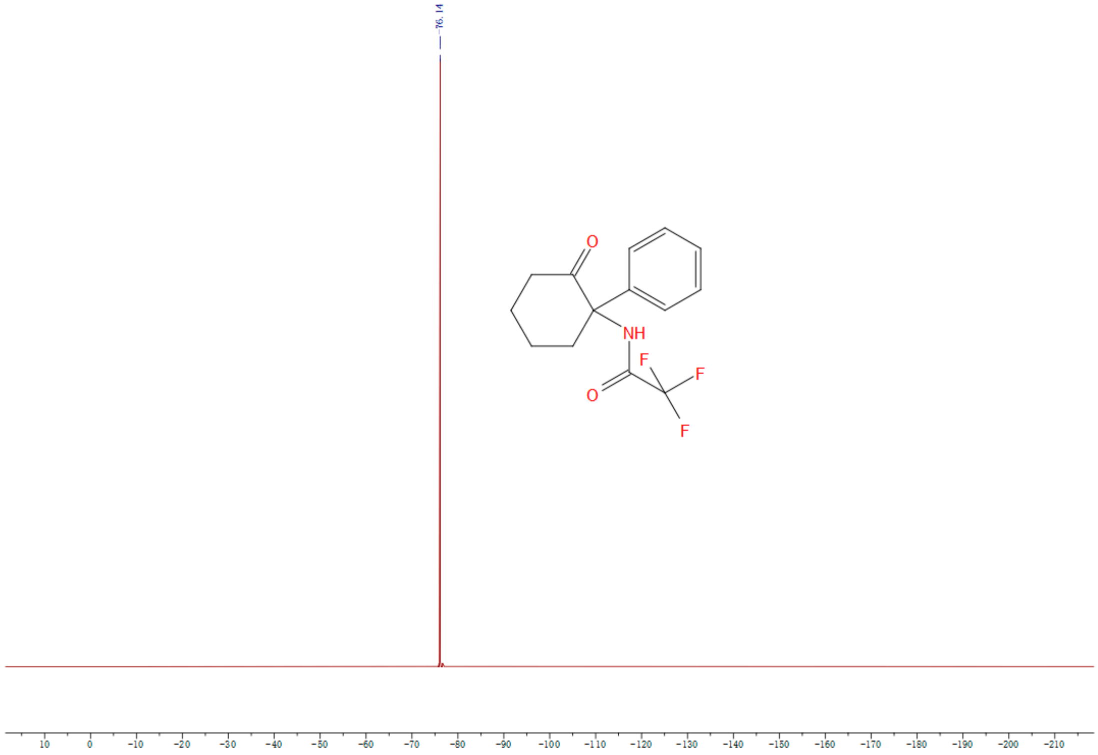

Figure S27 ^{19}F NMR spectrum of compound **17** ($CDCl_3$, 376 MHz)

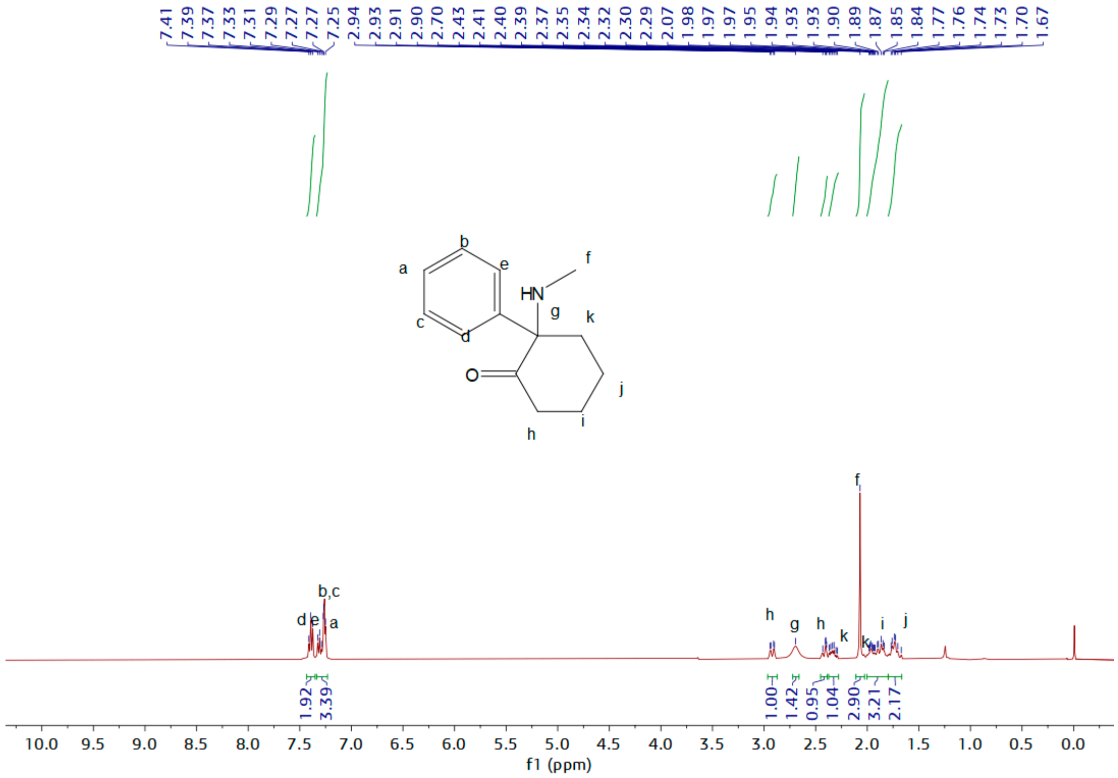

Figure S28 ^{1}H NMR spectrum of compound **18** ($CDCl_3$, 400 MHz)

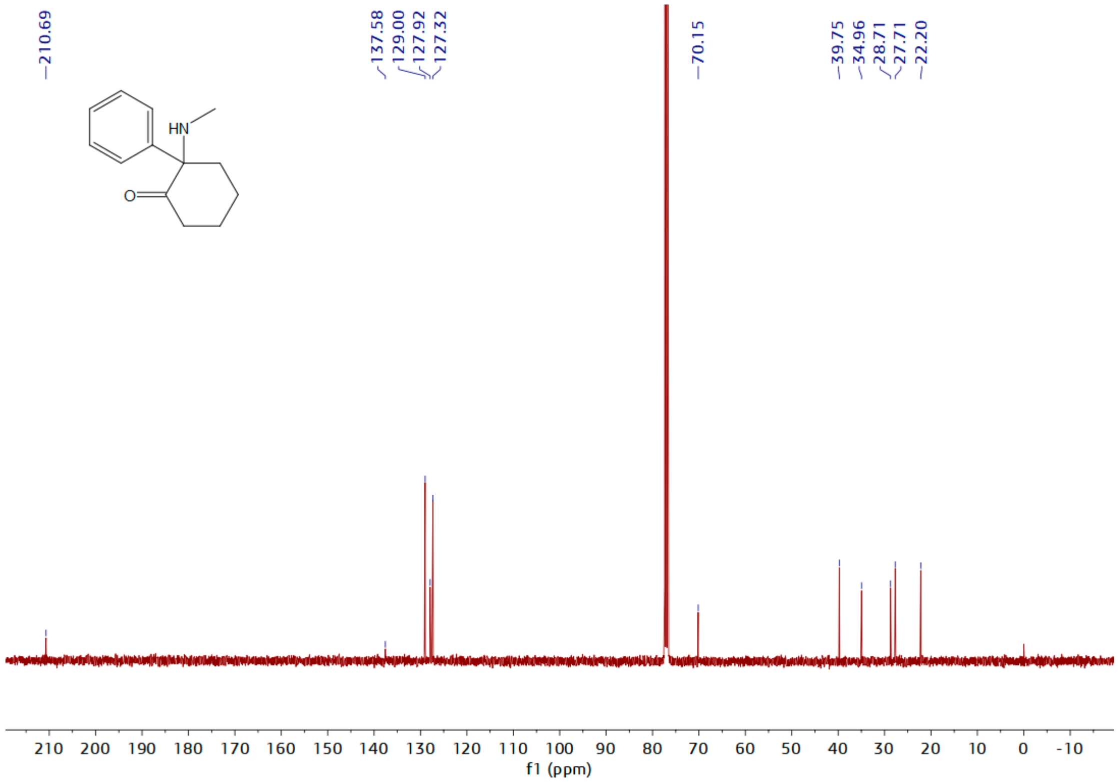

Figure S29 ^{13}C NMR spectrum of compound **18** ($CDCl_3$, 100 MHz)

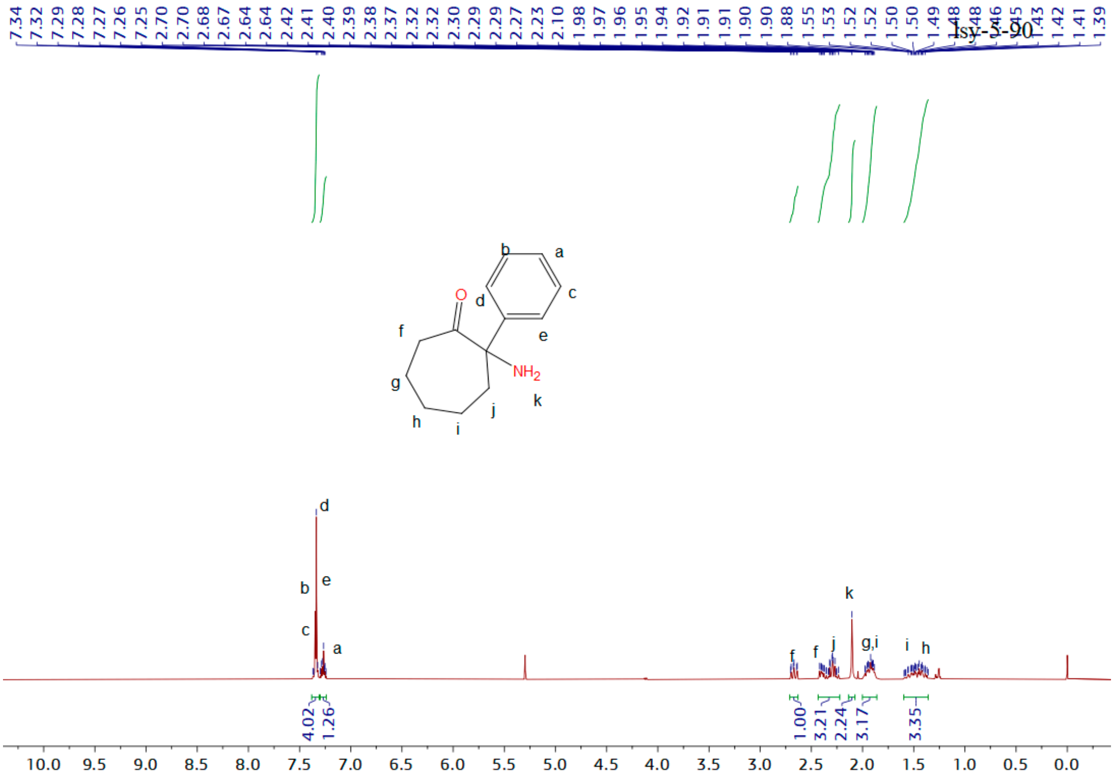

Figure S30 ^{1}H NMR spectrum of compound **19** ($CDCl_3$, 400 MHz)

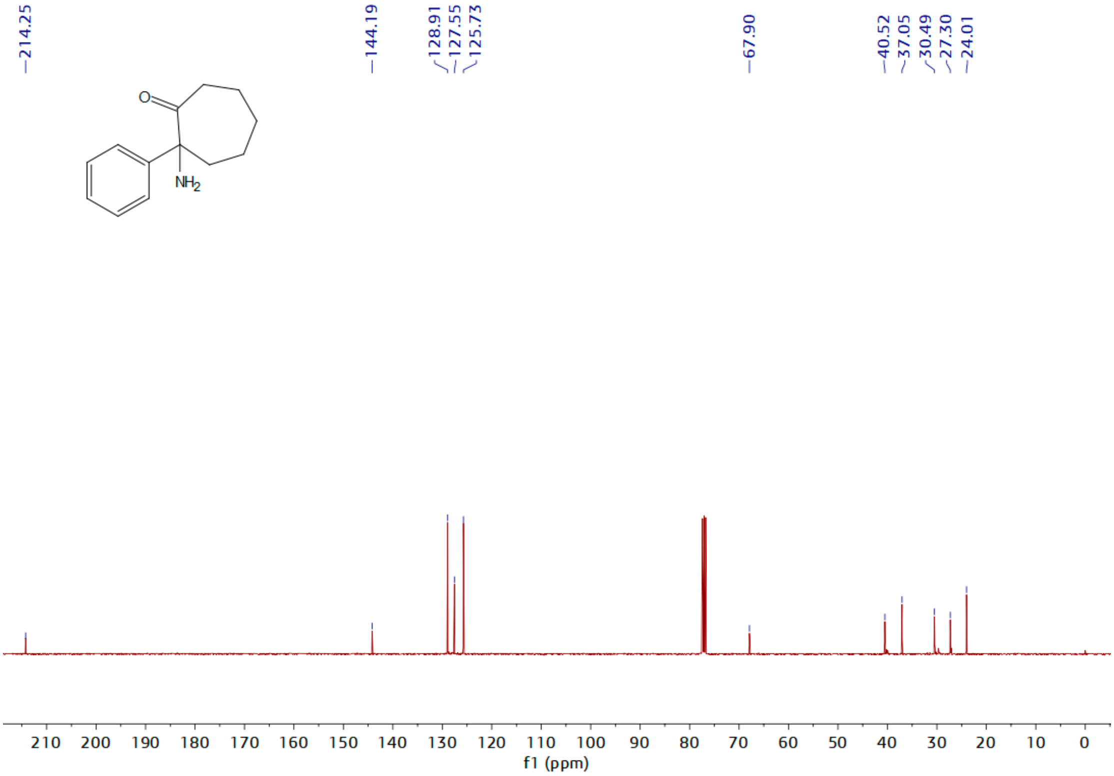

Figure S31 ^{13}C NMR spectrum of compound **19** ($CDCl_3$, 100 MHz)

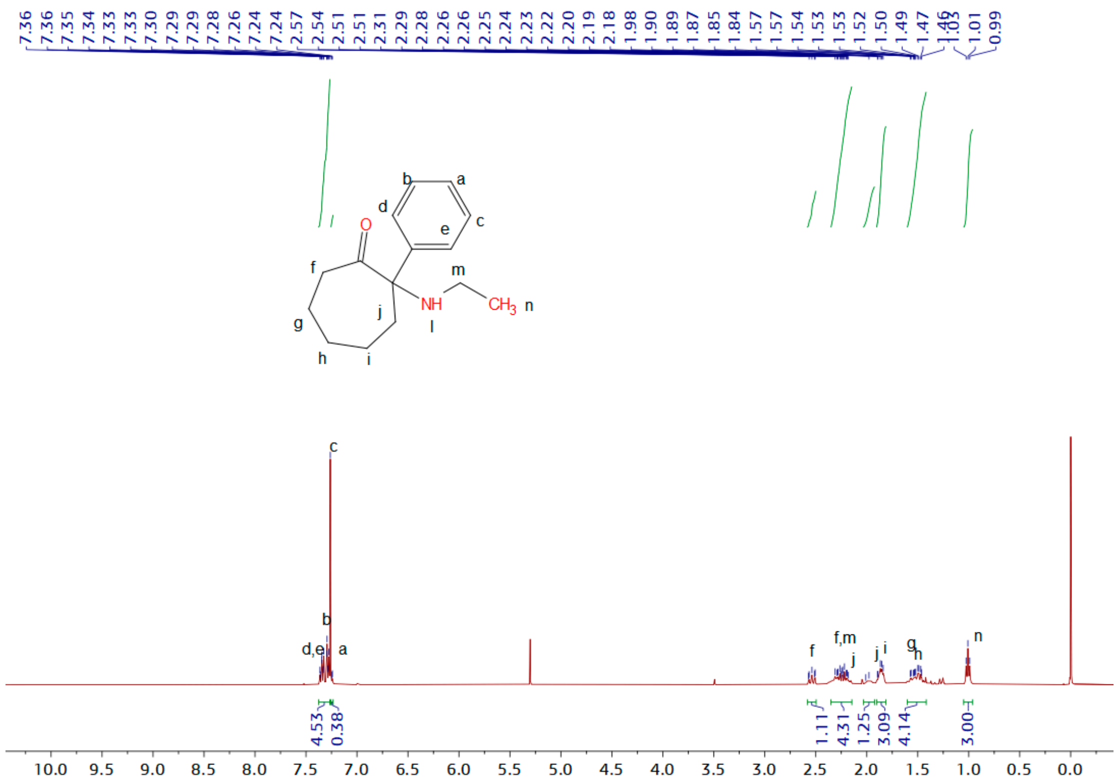

Figure S32 ^{1}H NMR spectrum of compound **20** ($CDCl_3$, 400 MHz)

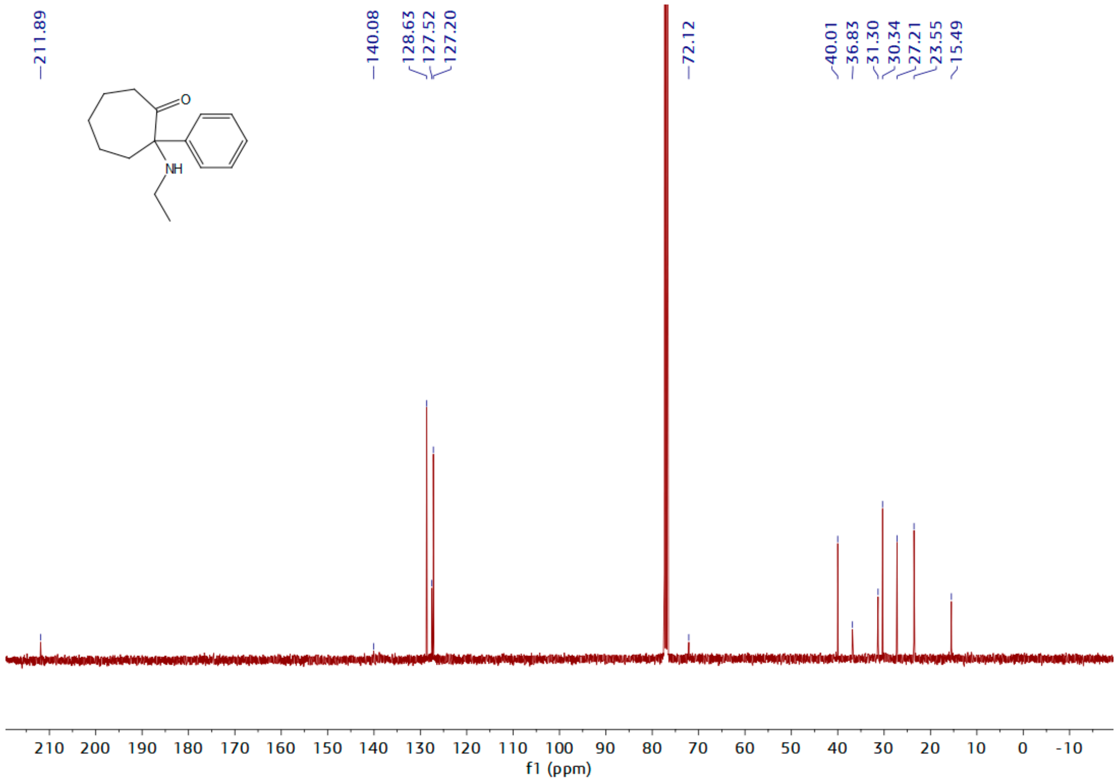

Figure S33 ^{13}C NMR spectrum of compound **20** ($CDCl_3$, 100 MHz)

Mass Spectrum SmartFormula Report

Analysis Info Acquisition Date 7/18/2022 8:22:43 AM

Analysis Name D:\Data\SHUJVFENXI\MADAWEIGROUP\2017195-LSY-5-103_BD1_01_30887.d
Method 20150915.m Operator BDAL@DE
Sample Name 2017195-LSY-5-103 Instrument / Ser# maXis 4G 21240
Comment

Acquisition Parameter

Source Type	ESI	Ion Polarity	Positive	Set Nebulizer	1.0 Bar
Focus	Not active	Set Capillary	4500 V	Set Dry Heater	220 °C
Scan Begin	50 m/z	Set End Plate Offset	-500 V	Set Dry Gas	6.0 l/min
Scan End	1500 m/z	Set Collision Cell RF	500.0 Vpp	Set Divert Valve	Waste

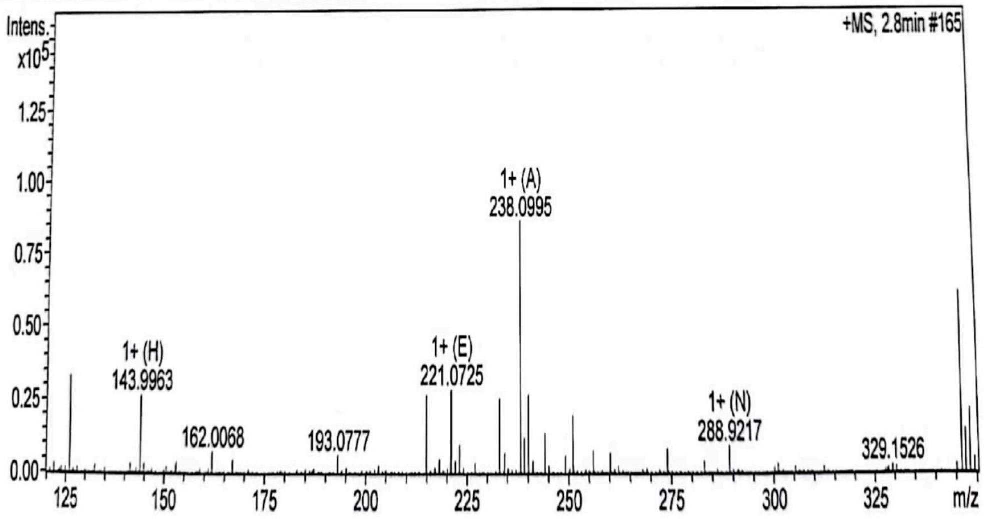

Meas. m/z	#	Formula	Score	m/z	err [ppm]	Mean err [ppm]	mSigma	rdb	e⁻ Conf	N-Rule
238.0995	1	C 13 H 17 Cl N O	100.00	238.0993	-0.7	-0.1	11.5	5.5	even	ok

Figure S34 HRMS spectrum of compound **20**

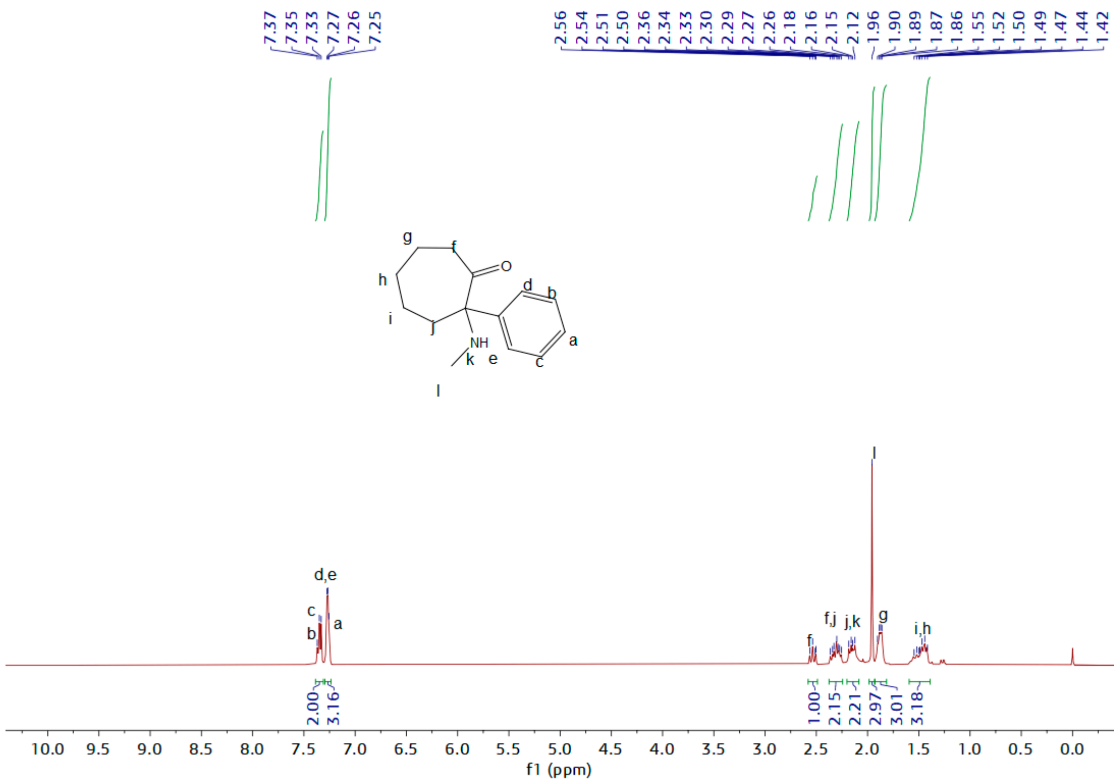

Figure S35 ^{1}H NMR spectrum of compound **21** ($CDCl_3$, 400 MHz)

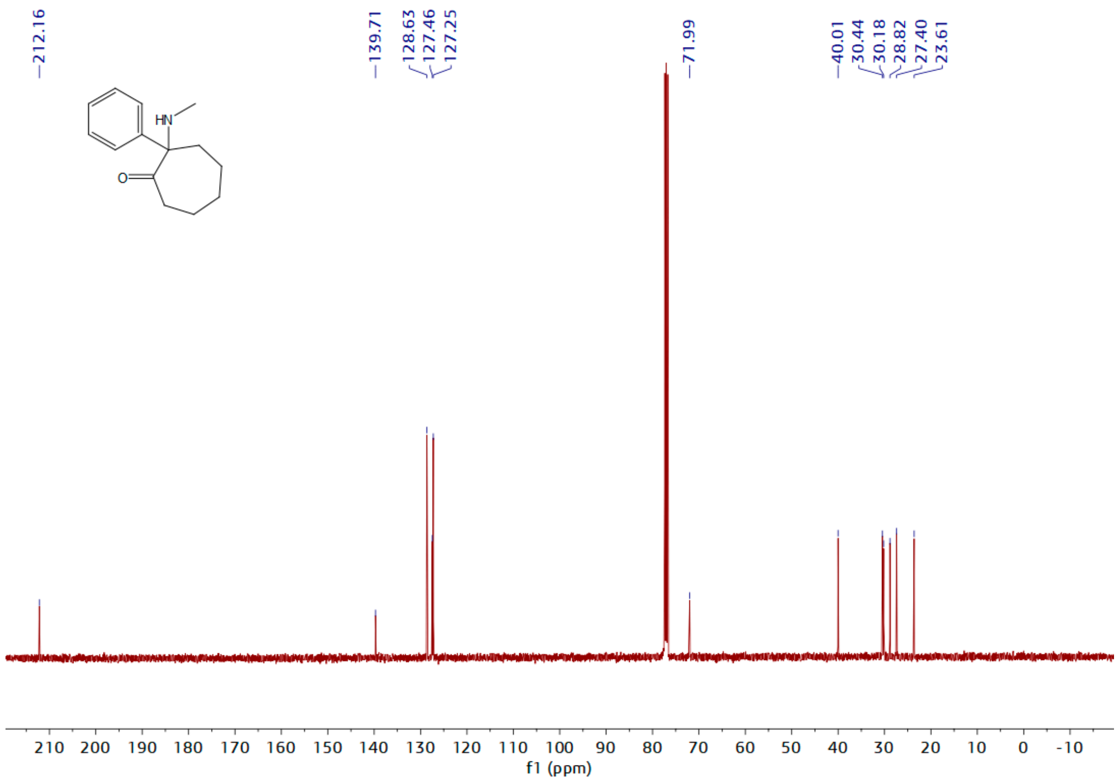

Figure S36 ^{13}C NMR spectrum of compound **21** ($CDCl_3$, 100 MHz)

Mass Spectrum SmartFormula Report

**Analysis Info**		Acquisition Date	7/18/2022 8:11:41 AM
Analysis Name	D:\Data\SHUJVFENXI\MADAWEIGROUP\2017195-LSY-5-96_BC7_01_30885.d		
Method	20150915.m	Operator	BDAL@DE
Sample Name	2017195-LSY-5-96	Instrument / Ser#	maXis 4G 21240
Comment			

Acquisition Parameter

Source Type	ESI	Ion Polarity	Positive	Set Nebulizer	1.0 Bar
Focus	Not active	Set Capillary	4500 V	Set Dry Heater	220 °C
Scan Begin	50 m/z	Set End Plate Offset	-500 V	Set Dry Gas	6.0 l/min
Scan End	1500 m/z	Set Collision Cell RF	500.0 Vpp	Set Divert Valve	Waste

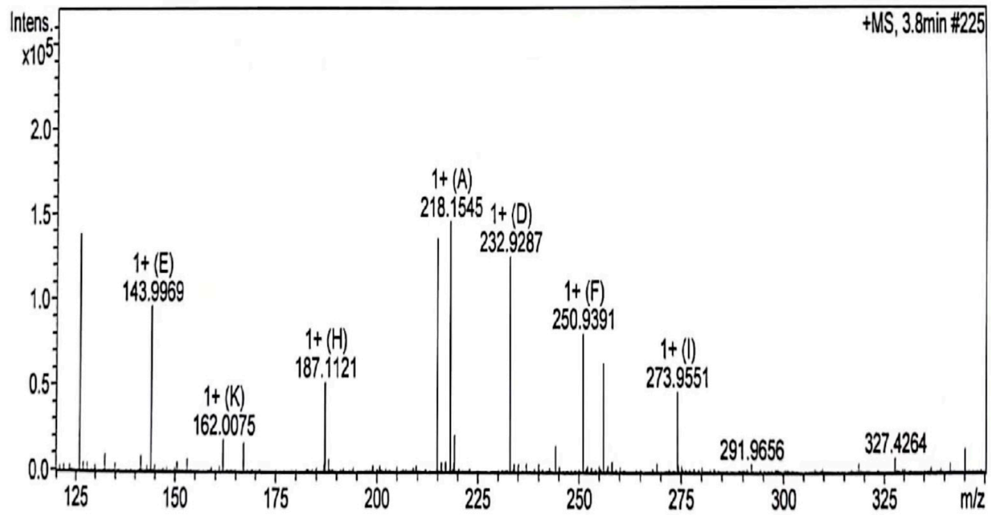

Meas. m/z	#	Formula	Score	m/z	err [ppm]	Mean err [ppm]	mSigma	rdb	e⁻ Conf	N-Rule
218.1545	1	C 14 H 20 N O	100.00	218.1539	-2.5	-2.2	4.1	5.5	even	ok

Figure S37 HRMS spectrum of compound **21**

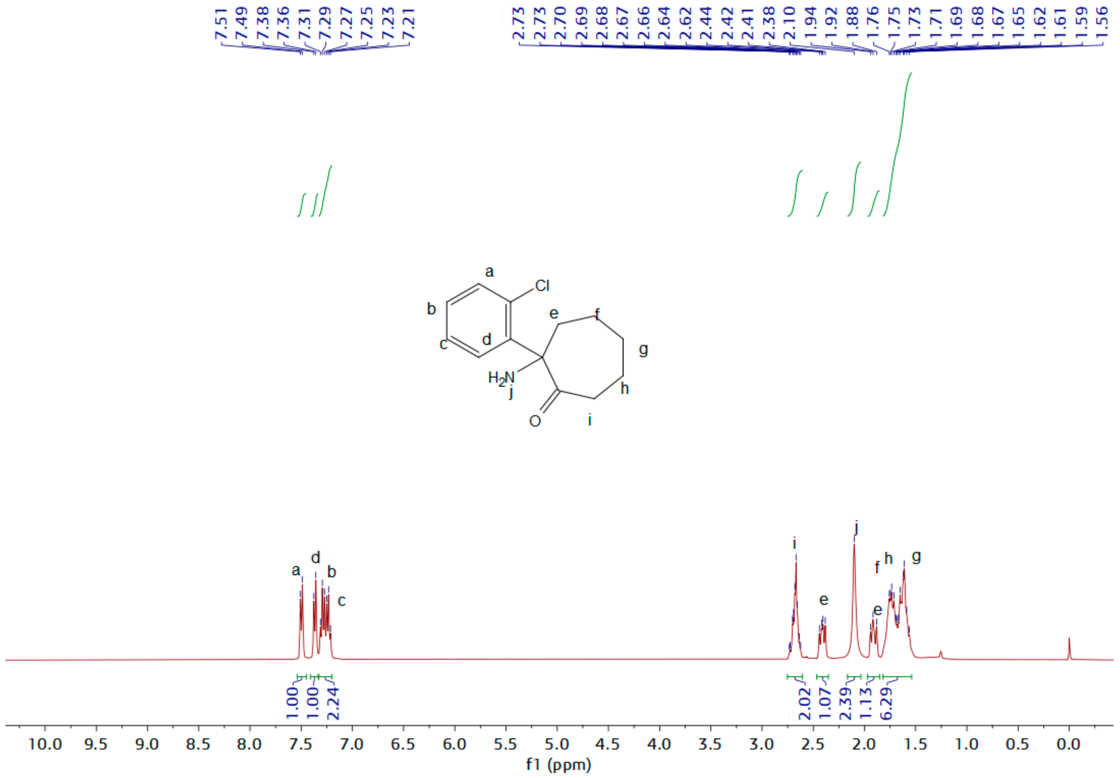

Figure S38 ^{1}H NMR spectrum of compound **22** ($CDCl_3$, 400 MHz)

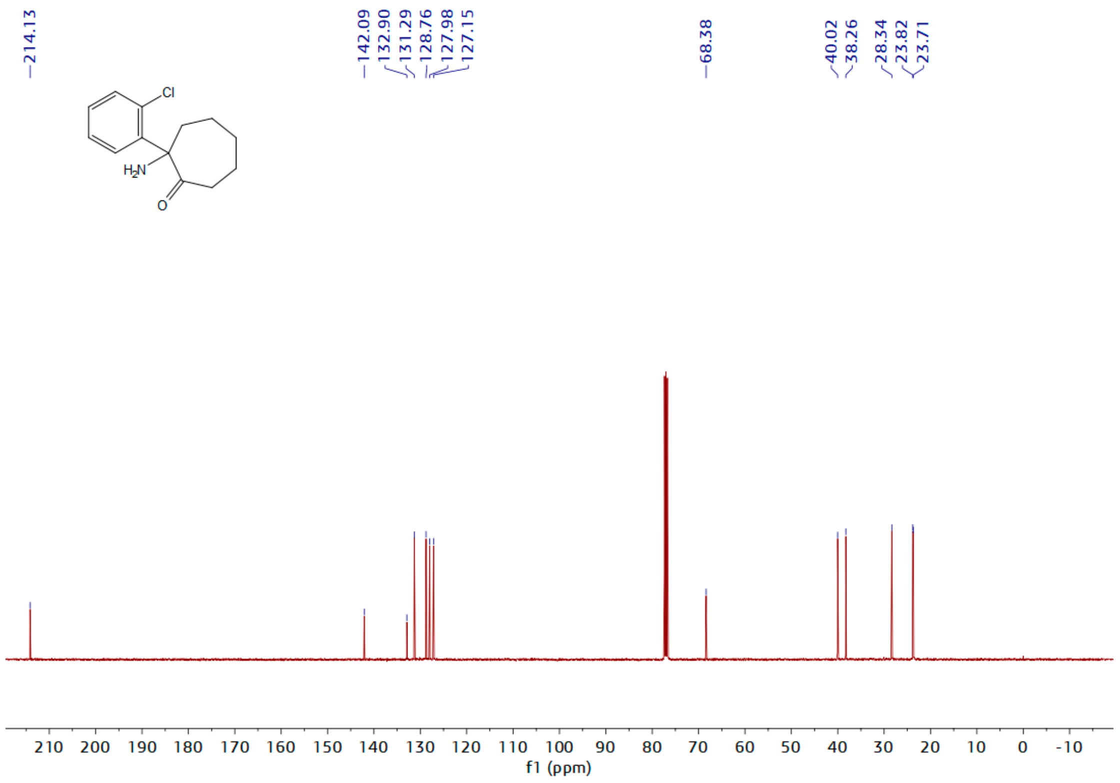

Figure S39 ^{13}C NMR spectrum of compound **22** ($CDCl_3$, 100 MHz)

Mass Spectrum SmartFormula Report

**Analysis Info**		Acquisition Date	7/18/2022 8:22:43 AM
Analysis Name	D:\Data\SHUJVFENXI\MADAWEIGROUP\2017195-LSY-5-103_BD1_01_30887.d		
Method	20150915.m	Operator	BDAL@DE
Sample Name	2017195-LSY-5-103	Instrument / Ser#	maXis 4G 21240
Comment			

Acquisition Parameter

Source Type	ESI	Ion Polarity	Positive	Set Nebulizer	1.0 Bar
Focus	Not active	Set Capillary	4500 V	Set Dry Heater	220 °C
Scan Begin	50 m/z	Set End Plate Offset	-500 V	Set Dry Gas	6.0 l/min
Scan End	1500 m/z	Set Collision Cell RF	500.0 Vpp	Set Divert Valve	Waste

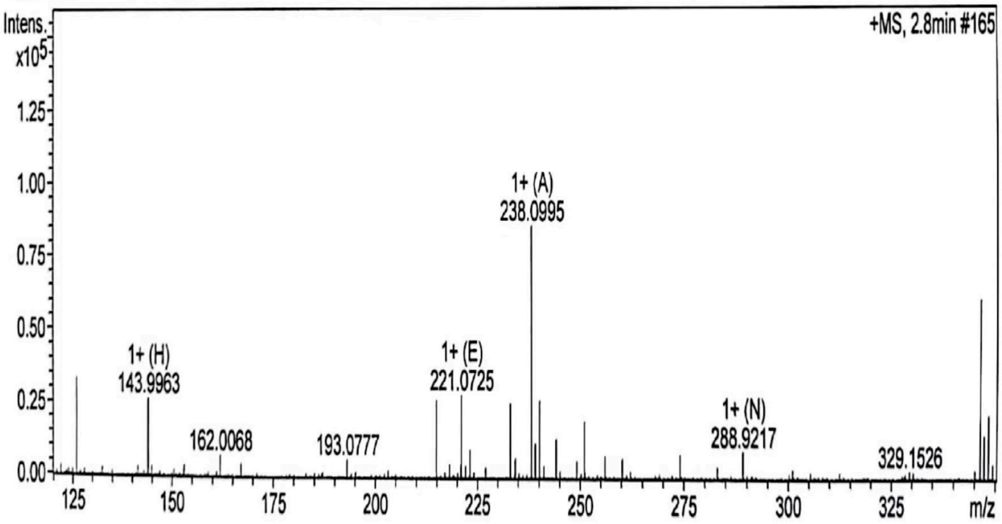

Meas. m/z	#	Formula	Score	m/z	err [ppm]	Mean err [ppm]	mSigma	rdb	e⁻ Conf	N-Rule
238.0995	1	C 13 H 17 Cl N O	100.00	238.0993	-0.7	-0.1	11.5	5.5	even	ok

Figure S40 HRMS spectrum of compound **22**

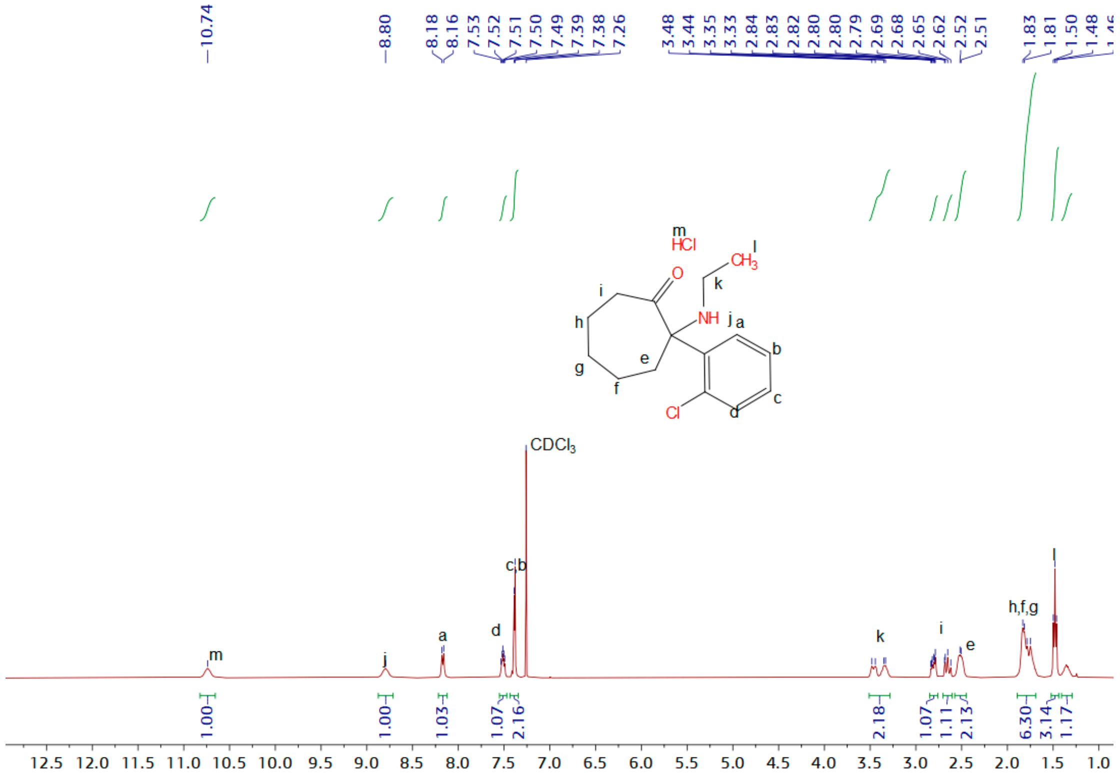

Figure S41 ^{1}H NMR spectrum of compound **23** ($CDCl_3$, 400 MHz)

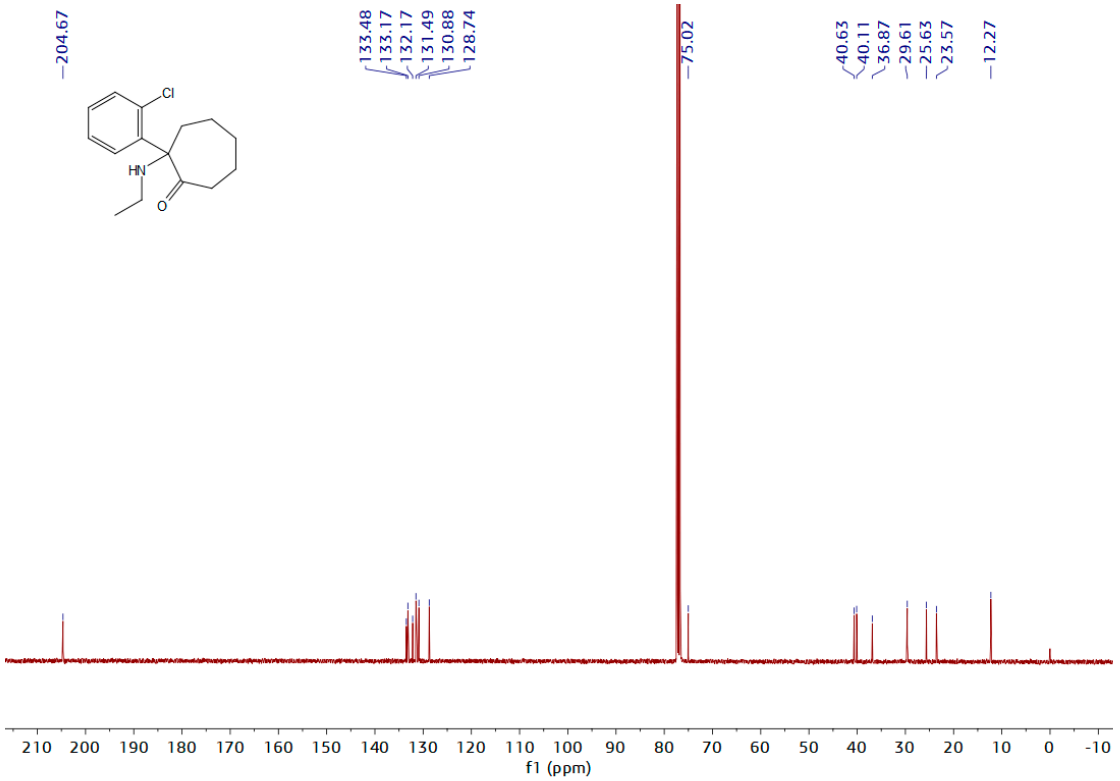

Figure S42 ^{13}C NMR spectrum of compound **23** ($CDCl_3$, 100 MHz)

Mass Spectrum SmartFormula Report

Analysis Info

Analysis Name	D:\Data\SHUJVFENXI\MADAWEIGROUP\2017195-LSY-6-1_BA3_01_31449.d	Acquisition Date	8/23/2022 9:46:39 AM
Method	20150915.m	Operator	BDAL@DE
Sample Name	2017195-LSY-6-1	Instrument / Ser#	maXis 4G 21240
Comment			

Acquisition Parameter

Source Type	ESI	Ion Polarity	Positive	Set Nebulizer	1.0 Bar
Focus	Not active	Set Capillary	4500 V	Set Dry Heater	220 °C
Scan Begin	50 m/z	Set End Plate Offset	-500 V	Set Dry Gas	6.0 l/min
Scan End	1500 m/z	Set Collision Cell RF	500.0 Vpp	Set Divert Valve	Waste

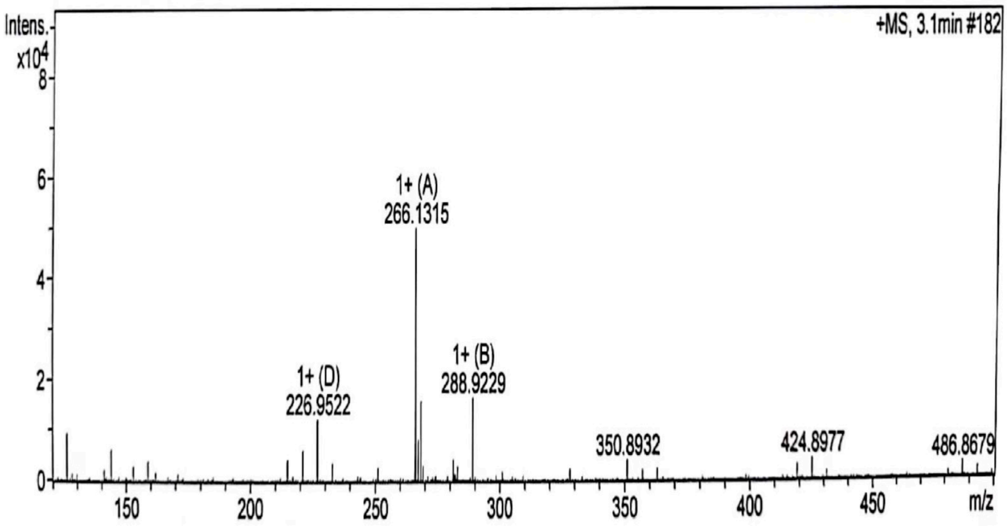

Meas. m/z	#	Formula	Score	m/z	err [ppm]	Mean err [ppm]	mSigma	rdb	e⁻ Conf	N-Rule
266.1315	1	C 15 H 21 Cl N O	100.00	266.1306	-3.1	-3.3	9.4	5.5	even	ok

Figure S43 HRMS spectrum of compound **23**

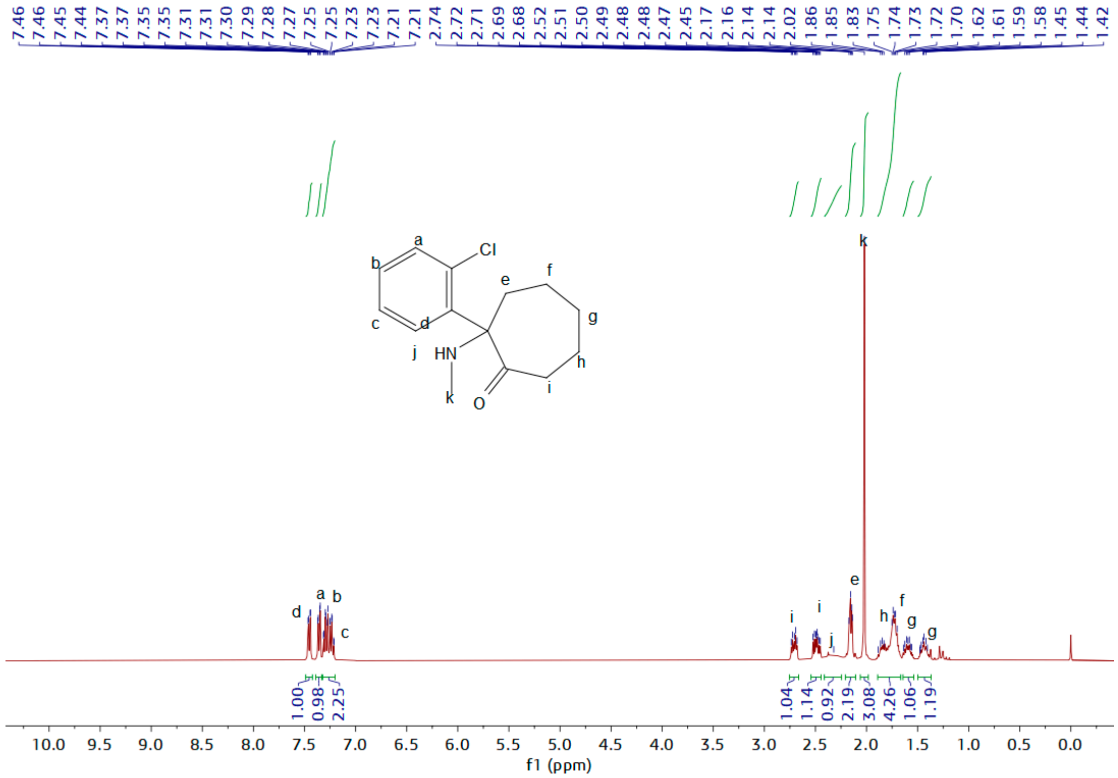

Figure S44 ^{1}H NMR spectrum of compound **24** ($CDCl_3$, 400 MHz)

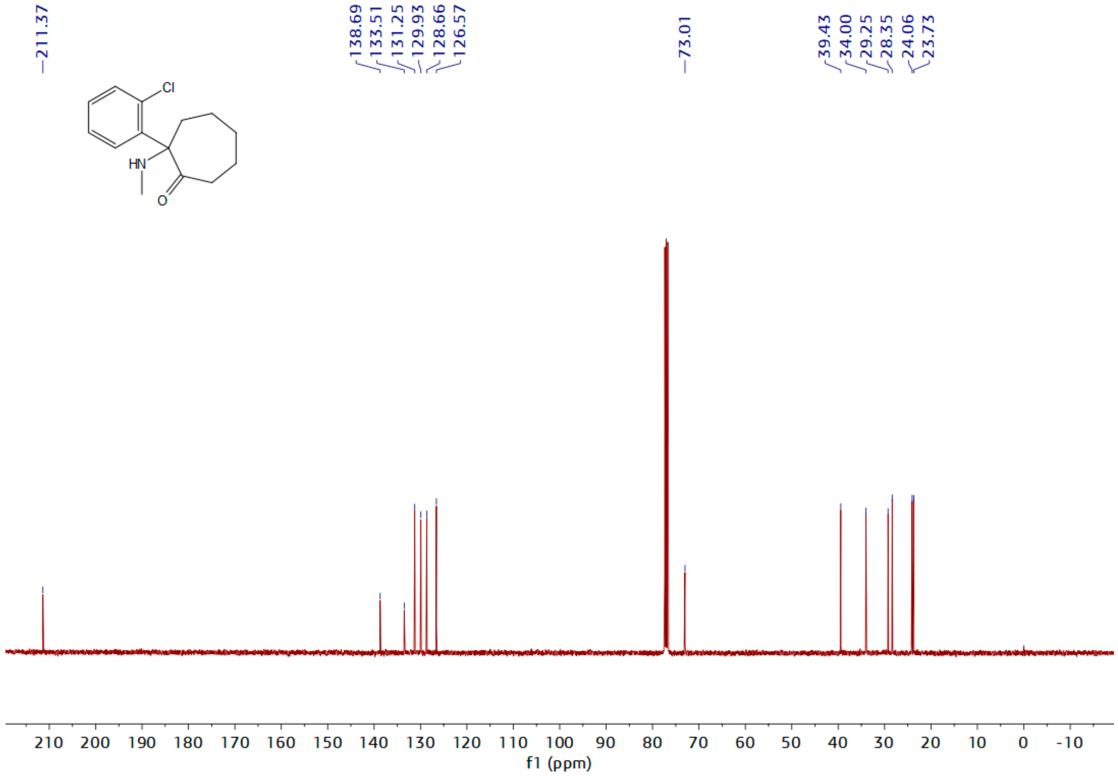

Figure S45 ^{13}C NMR spectrum of compound **24** ($CDCl_3$, 100 MHz)

Mass Spectrum SmartFormula Report

Analysis Info

Analysis Name	D:\Data\SHUJVFENXI\MADAWEIGROUP\2017195-LSY-18-77_BB5_01_31162.d	Acquisition Date	8/8/2022 6:00:47 AM
Method	20150915.m	Operator	BDAL@DE
Sample Name	2017195-LSY-18-77	Instrument / Ser#	maXis 4G 21240
Comment			

Acquisition Parameter

Source Type	ESI	Ion Polarity	Positive	Set Nebulizer	1.0 Bar
Focus	Not active	Set Capillary	4500 V	Set Dry Heater	220 °C
Scan Begin	50 m/z	Set End Plate Offset	-500 V	Set Dry Gas	6.0 l/min
Scan End	1500 m/z	Set Collision Cell RF	500.0 Vpp	Set Divert Valve	Waste

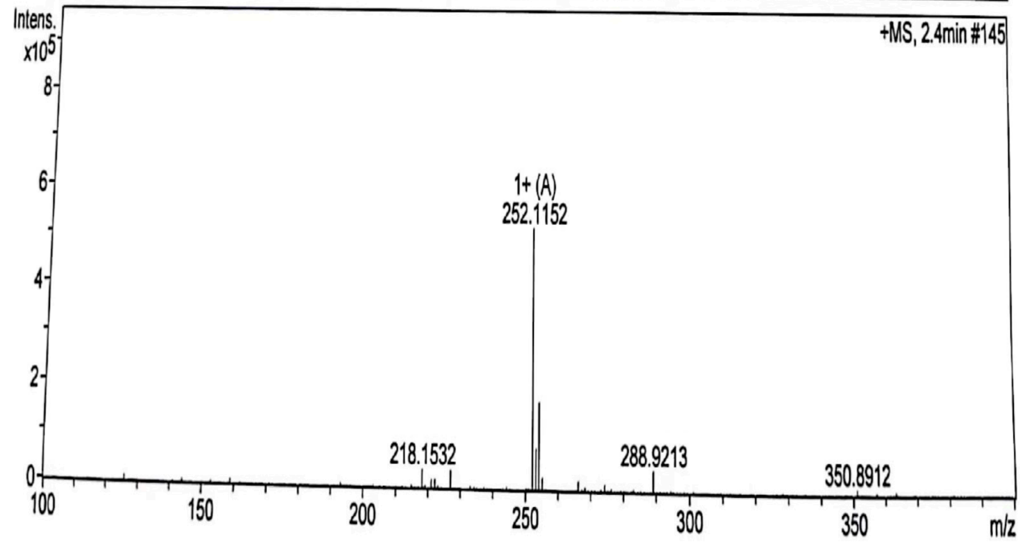

Meas. m/z	#	Formula	Score	m/z	err [ppm]	Mean err [ppm]	mSigma	rdb	e⁻ Conf	N-Rule
252.1152	1	C 14 H 19 Cl N O	100.00	252.1150	-1.0	-0.2	3.2	5.5	even	ok

Figure S46 HRMS spectrum of compound **24**

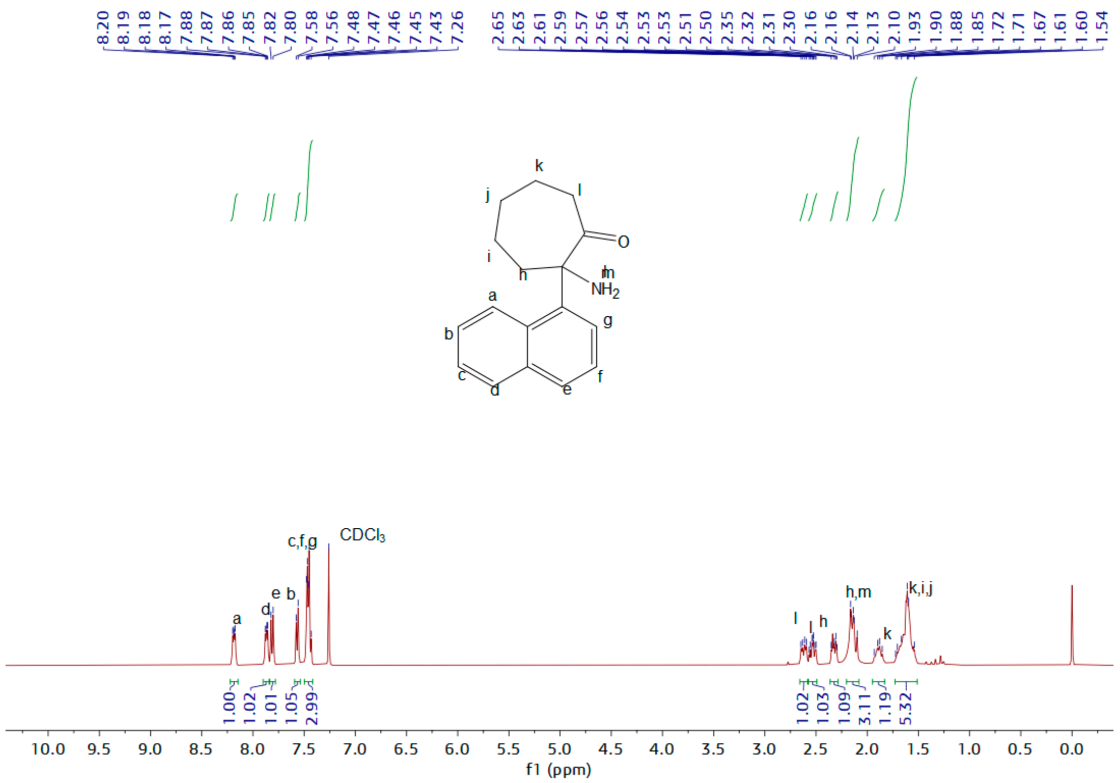

Figure S47 ^{1}H NMR spectrum of compound **25** ($CDCl_3$, 400 MHz)

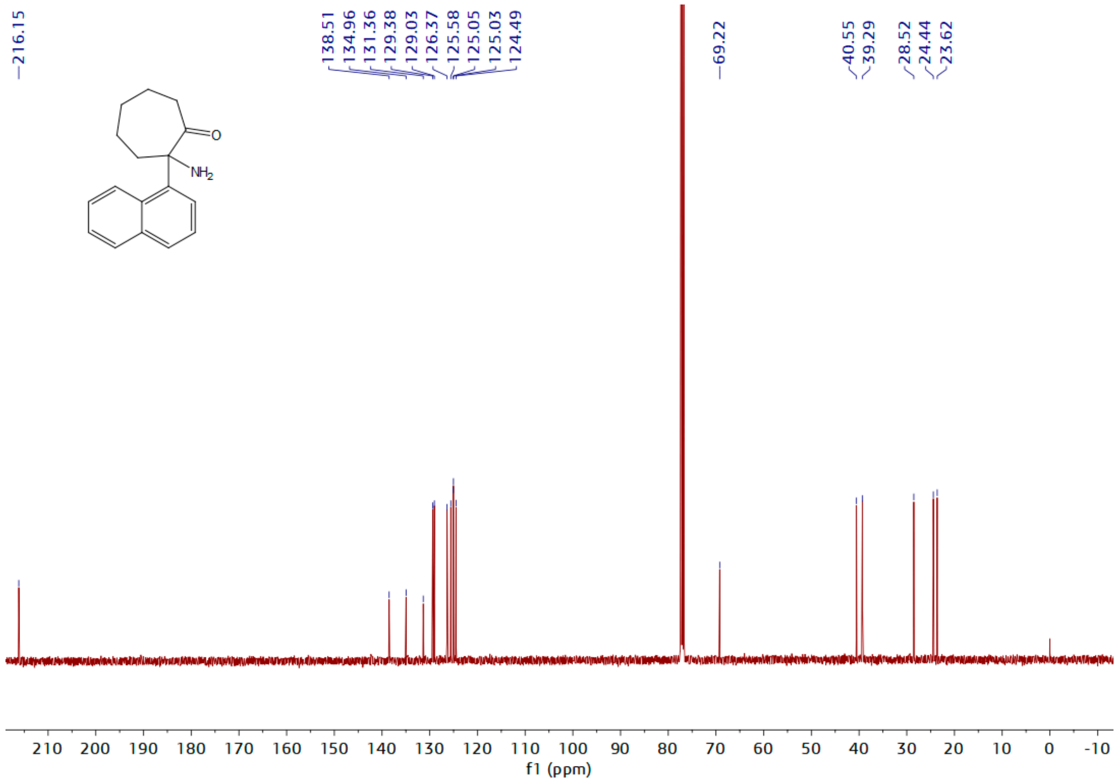

Figure S48 ^{13}C NMR spectrum of compound **25** ($CDCl_3$, 100 MHz)

Mass Spectrum SmartFormula Report

Analysis Info

Analysis Name	D:\Data\SHUJVFENXI\MADAWEIGROUP\2017195-LSY-6-6_RD1_01_31432.d	Acquisition Date	8/23/2022 7:57:31 AM
Method	20150915.m	Operator	BDAL@DE
Sample Name	2017195-LSY-6-6	Instrument / Ser#	maXis 4G 21240
Comment			

Acquisition Parameter

Source Type	ESI	Ion Polarity	Positive	Set Nebulizer	1.0 Bar
Focus	Not active	Set Capillary	4500 V	Set Dry Heater	220 °C
Scan Begin	50 m/z	Set End Plate Offset	-500 V	Set Dry Gas	6.0 l/min
Scan End	1500 m/z	Set Collision Cell RF	500.0 Vpp	Set Divert Valve	Waste

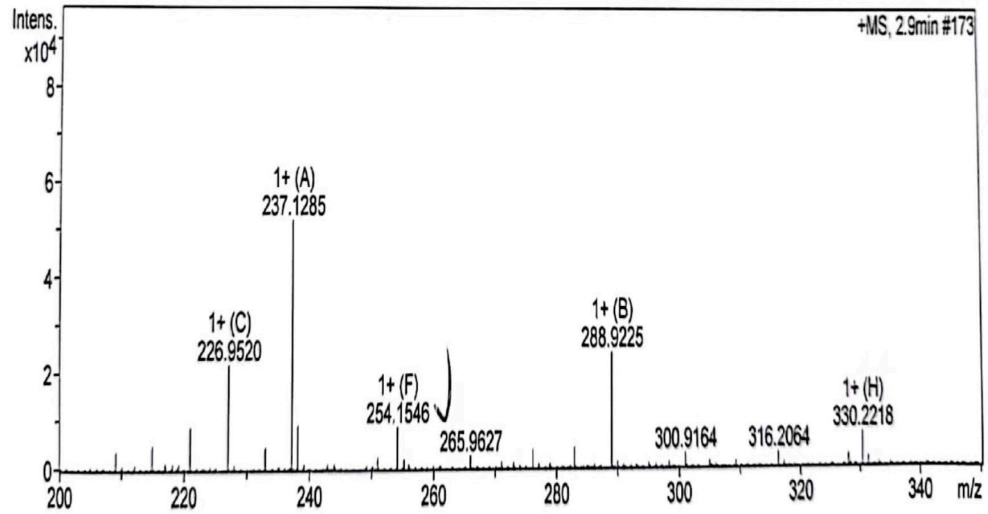

Meas. m/z	#	Formula	Score	m/z	err [ppm]	Mean err [ppm]	mSigma	rdb	e⁻ Conf	N-Rule
254.1546	1	C 17 H 20 N O	100.00	254.1539	-2.5	-2.5	13.2	8.5	even	ok

Figure S49 HRMS spectrum of compound **25**

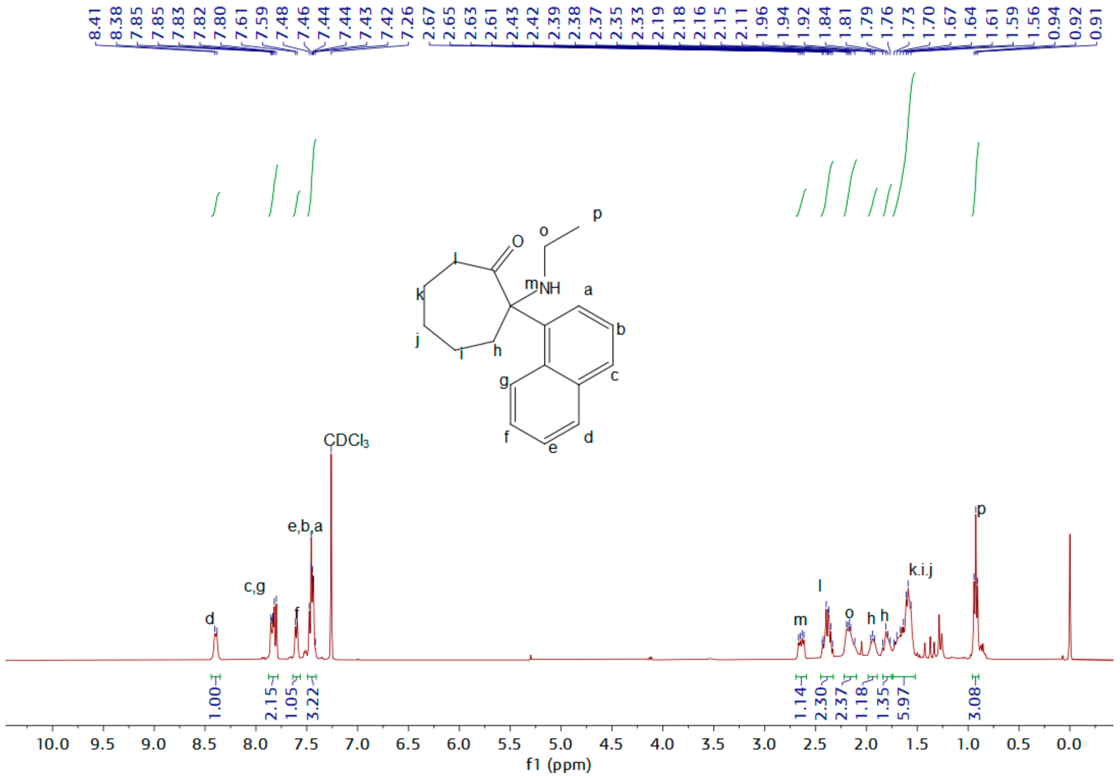

Figure S50 ^{1}H NMR spectrum of compound **26** ($CDCl_3$, 400 MHz)

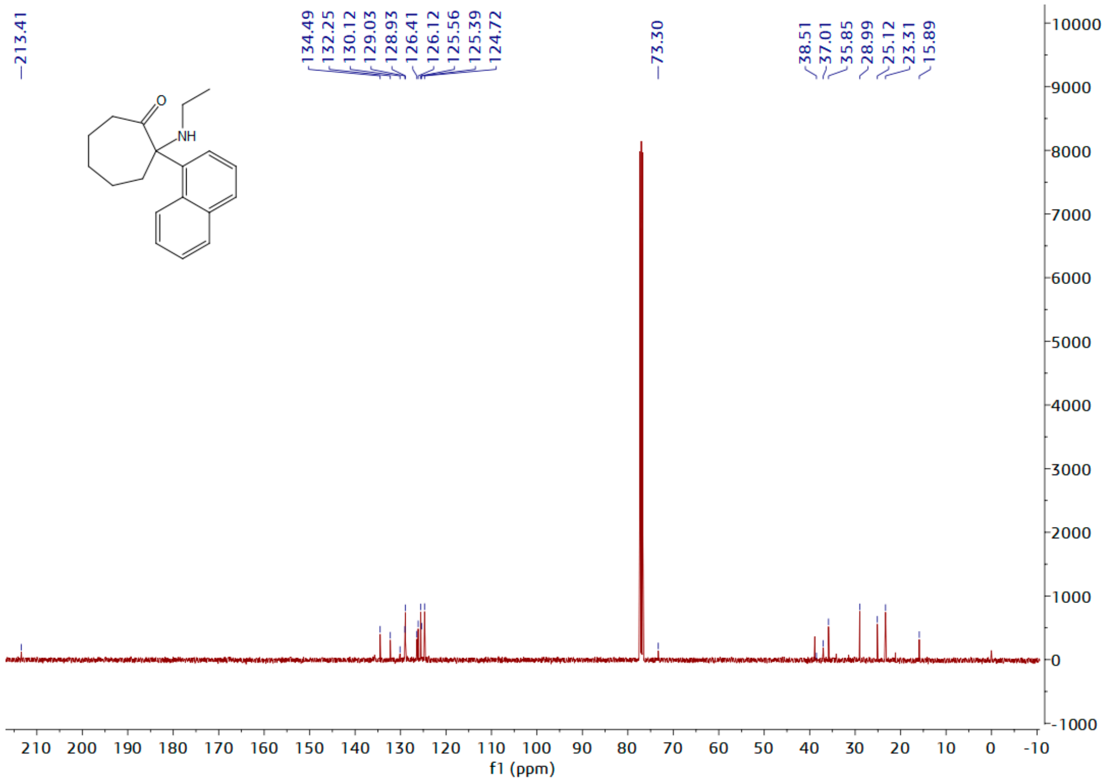

Figure S51 ^{13}C NMR spectrum of compound **26** ($CDCl_3$, 100 MHz)

Mass Spectrum SmartFormula Report

Analysis Info

Analysis Name	D:\Data\SHUJVFENXI\MADAWEIGROUP\2017195-LSY-6-7_BA6_01_31452.d	Acquisition Date	8/23/2022 10:05:48 AM
Method	20150915.m	Operator	BDAL@DE
Sample Name	2017195-LSY-6-7	Instrument / Ser#	maXis 4G 21240
Comment			

Acquisition Parameter

Source Type	ESI	Ion Polarity	Positive	Set Nebulizer	1.0 Bar
Focus	Not active	Set Capillary	4500 V	Set Dry Heater	220 °C
Scan Begin	50 m/z	Set End Plate Offset	-500 V	Set Dry Gas	6.0 l/min
Scan End	1500 m/z	Set Collision Cell RF	500.0 Vpp	Set Divert Valve	Waste

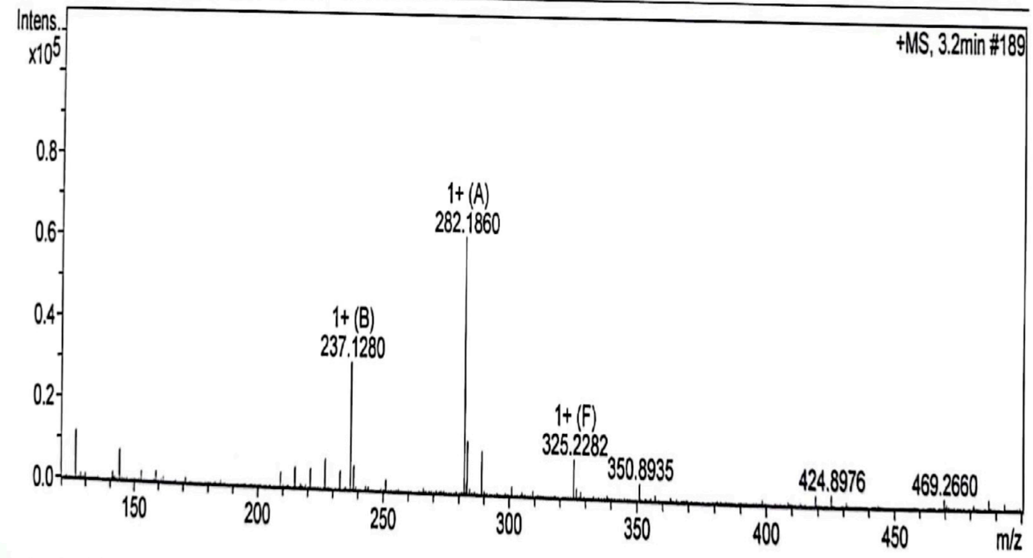

Meas. m/z	#	Formula	Score	m/z	err [ppm]	Mean err [ppm]	mSigma	rdb	e⁻ Conf	N-Rule
282.1860	1	C 19 H 24 N O	100.00	282.1852	-2.8	-2.6	2.8	8.5	even	ok

Figure S52 HRMS spectrum of compound **26**

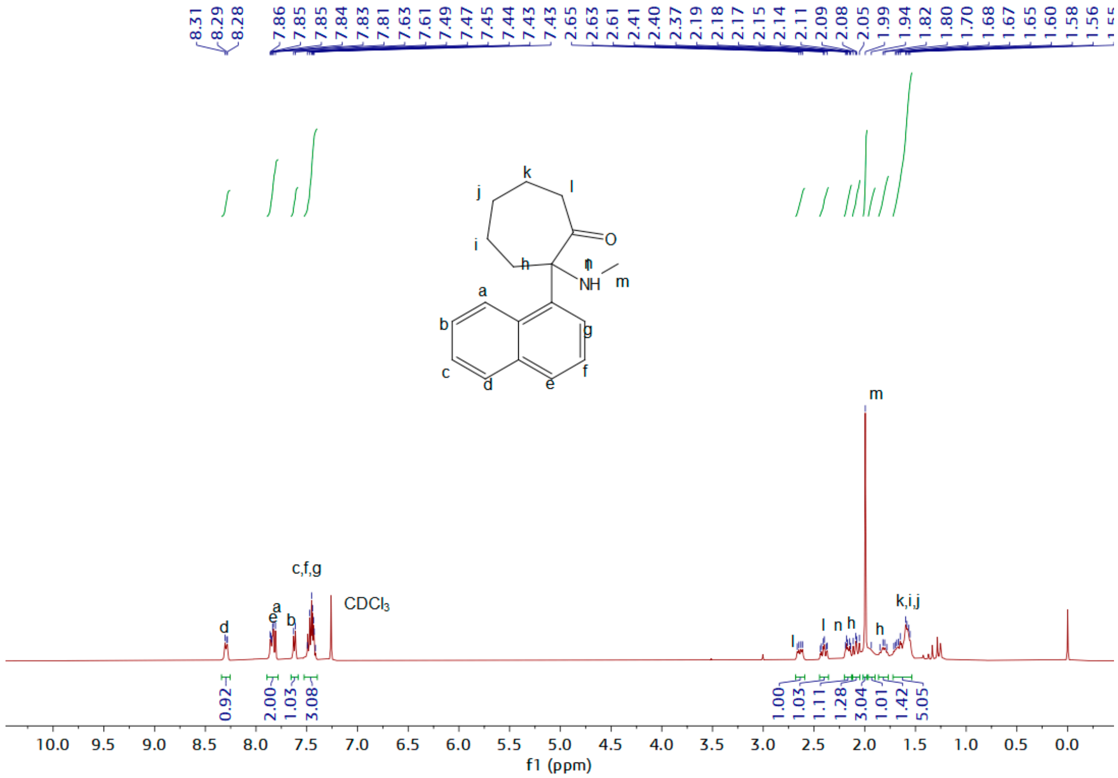

Figure S53 ^{1}H NMR spectrum of compound **27** ($CDCl_3$, 400 MHz)

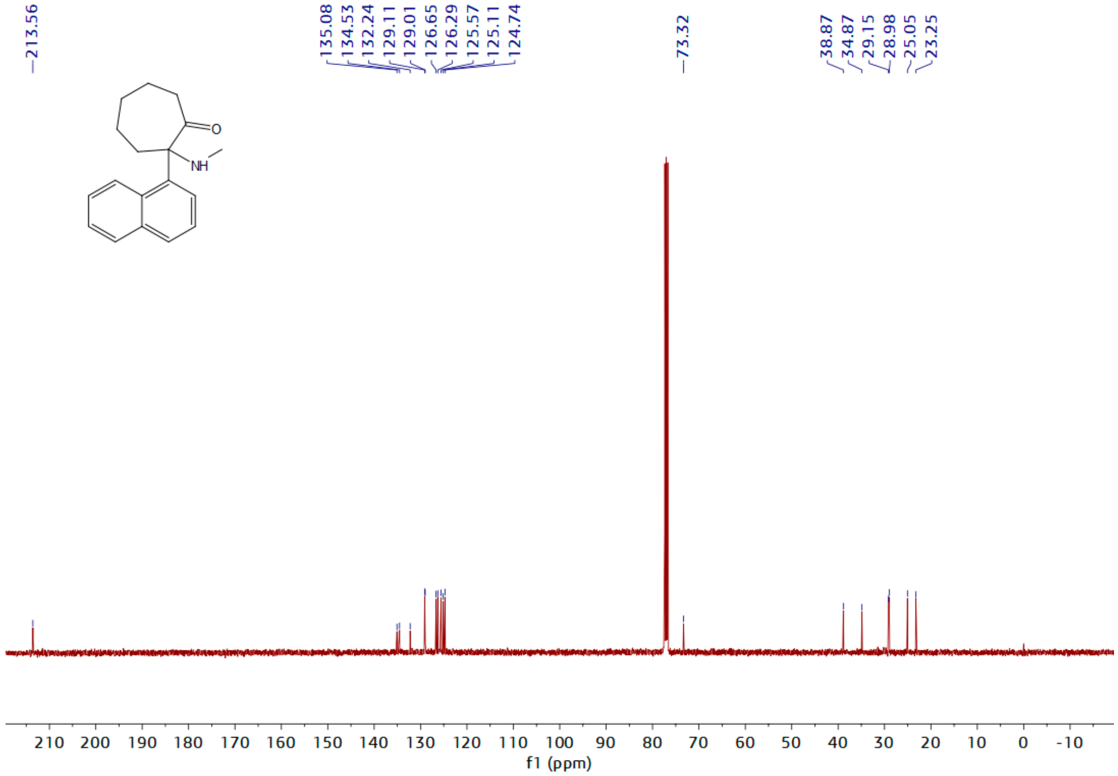

Figure S54 ^{13}C NMR spectrum of compound **27** ($CDCl_3$, 100 MHz)

Mass Spectrum SmartFormula Report

Analysis Info

Analysis Name	D:\Data\SHUJVFENXI\MADAWEIGROUP\2017195-LSY-18-92-2_BB2_01_31171.d	Acquisition Date	8/8/2022 11:40:46 PM
Method	20150915.m	Operator	BDAL@DE
Sample Name	2017195-LSY-18-92-2	Instrument / Ser#	maXis 4G 21240
Comment			

Acquisition Parameter

Source Type	ESI	Ion Polarity	Positive	Set Nebulizer	1.0 Bar
Focus	Not active	Set Capillary	4500 V	Set Dry Heater	220 °C
Scan Begin	50 m/z	Set End Plate Offset	-500 V	Set Dry Gas	6.0 l/min
Scan End	1500 m/z	Set Collision Cell RF	500.0 Vpp	Set Divert Valve	Waste

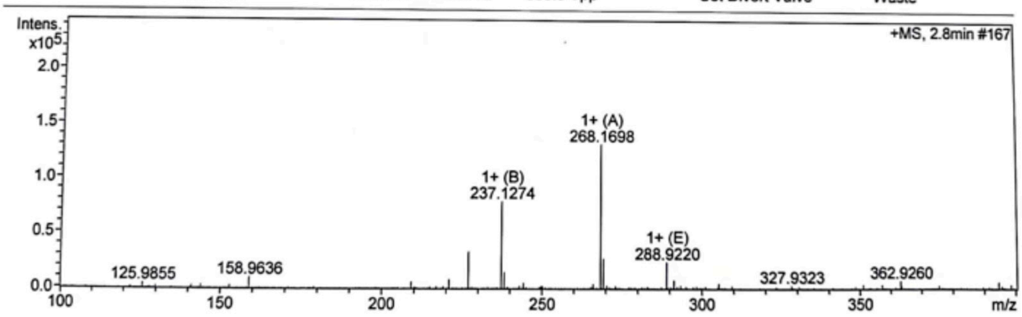

Meas. m/z	#	Formula	Score	m/z	err [ppm]	Mean err [ppm]	mSigma	rdb	e⁻ Conf	N-Rule
268.1698	1	C 18 H 22 N O	100.00	268.1696	-0.6	-0.4	4.5	8.5	even	ok

Figure S55 HRMS spectrum of compound **27**

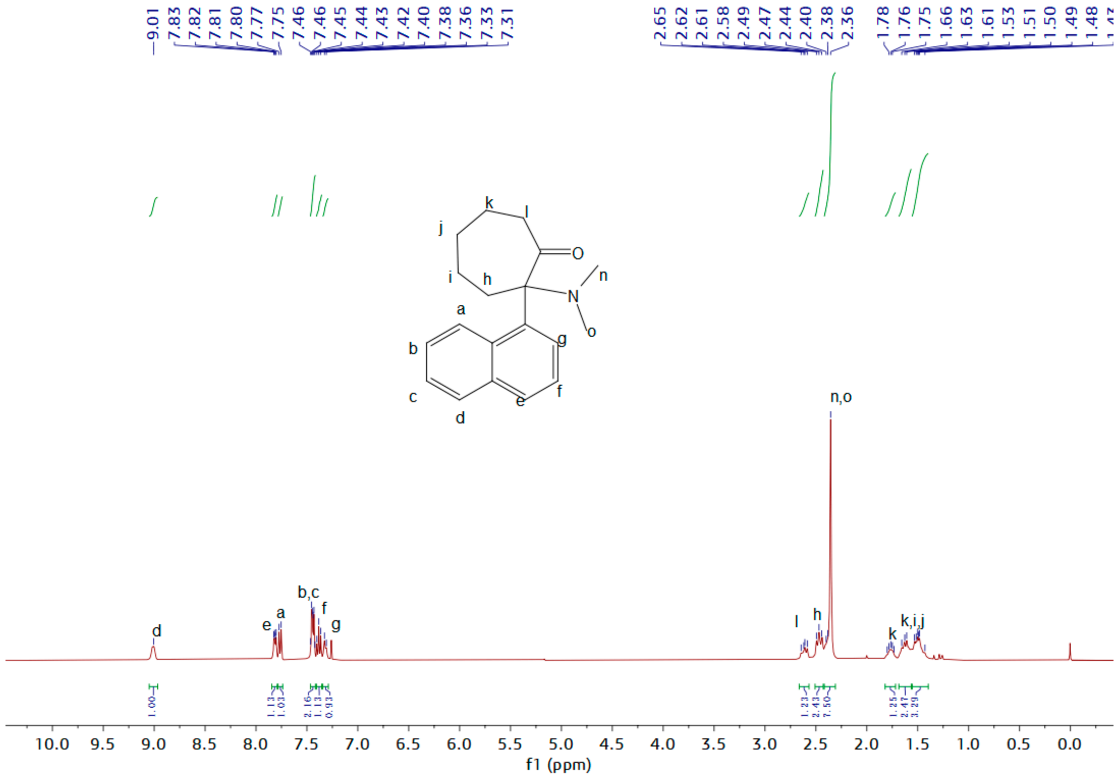

Figure S56 ^{1}H NMR spectrum of compound **28** ($CDCl_3$, 400 MHz)

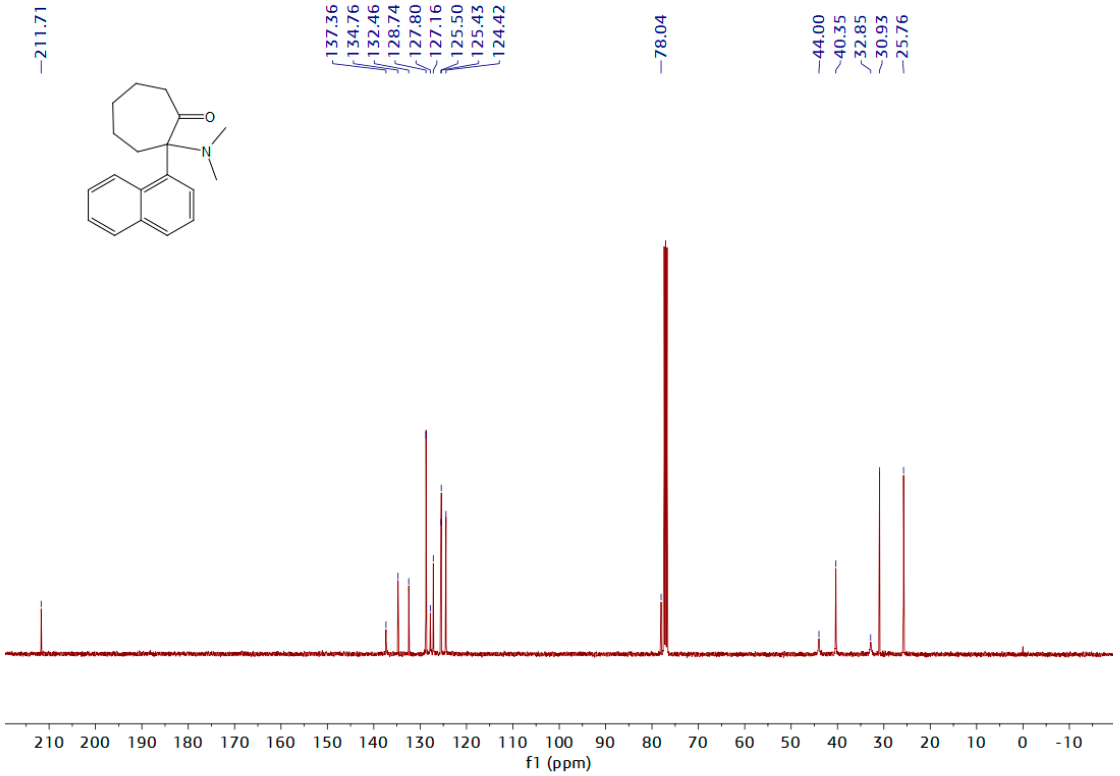

Figure S57 ^{13}C NMR spectrum of compound **28** ($CDCl_3$, 100 MHz)

Mass Spectrum SmartFormula Report

Analysis Info

Analysis Name	D:\Data\SHUJVFENXI\MADAWEIGROUP\2017195-LSY-18-92-1_RB1_01_31172.d	Acquisition Date	8/8/2022 11:47:20 PM
Method	20150915.m	Operator	BDAL@DE
Sample Name	2017195-LSY-18-92-1	Instrument / Ser#	maXis 4G 21240
Comment			

Acquisition Parameter

Source Type	ESI	Ion Polarity	Positive	Set Nebulizer	1.0 Bar
Focus	Not active	Set Capillary	4500 V	Set Dry Heater	220 °C
Scan Begin	50 m/z	Set End Plate Offset	-500 V	Set Dry Gas	6.0 l/min
Scan End	1500 m/z	Set Collision Cell RF	500.0 Vpp	Set Divert Valve	Waste

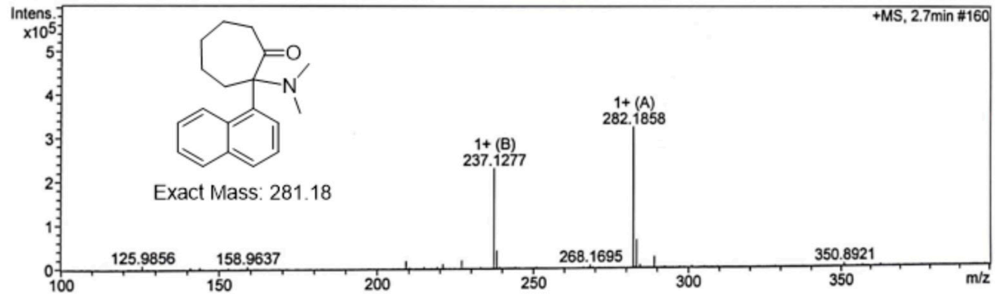

Meas. m/z	#	Formula	Score	m/z	err [ppm]	Mean err [ppm]	mSigma	rdb	e⁻ Conf	N-Rule
282.1858	1	C 19 H 24 N O	100.00	282.1852	-1.9	-1.7	6.6	8.5	even	ok

Figure S58 HRMS spectrum of compound **28**

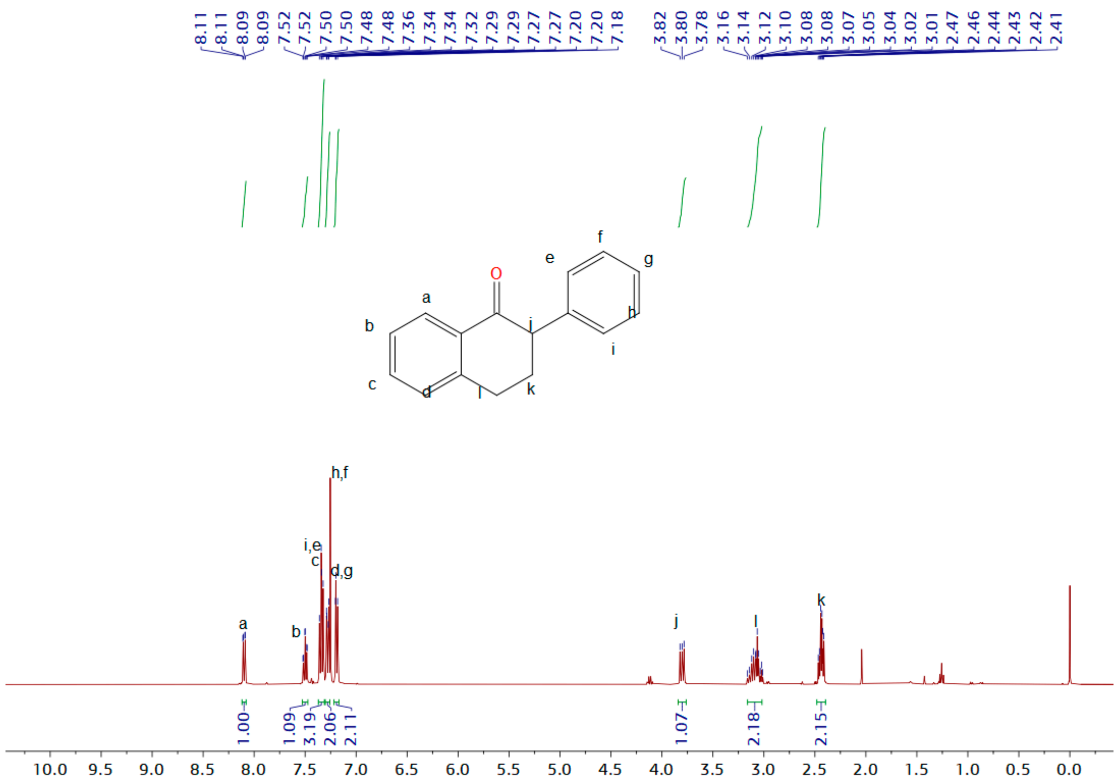

Figure S59 ^{1}H NMR spectrum of compound **29a** ($CDCl_3$, 400 MHz)

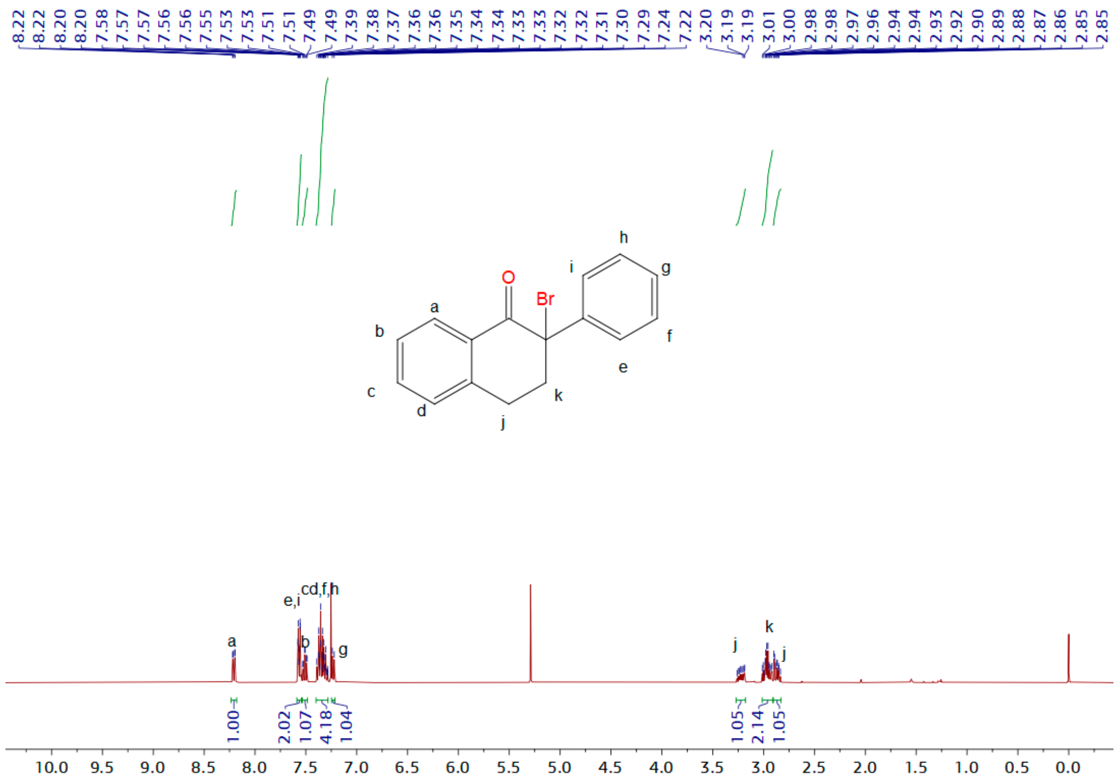

Figure S60 ^{1}H NMR spectrum of compound **32a** ($CDCl_3$, 400 MHz)

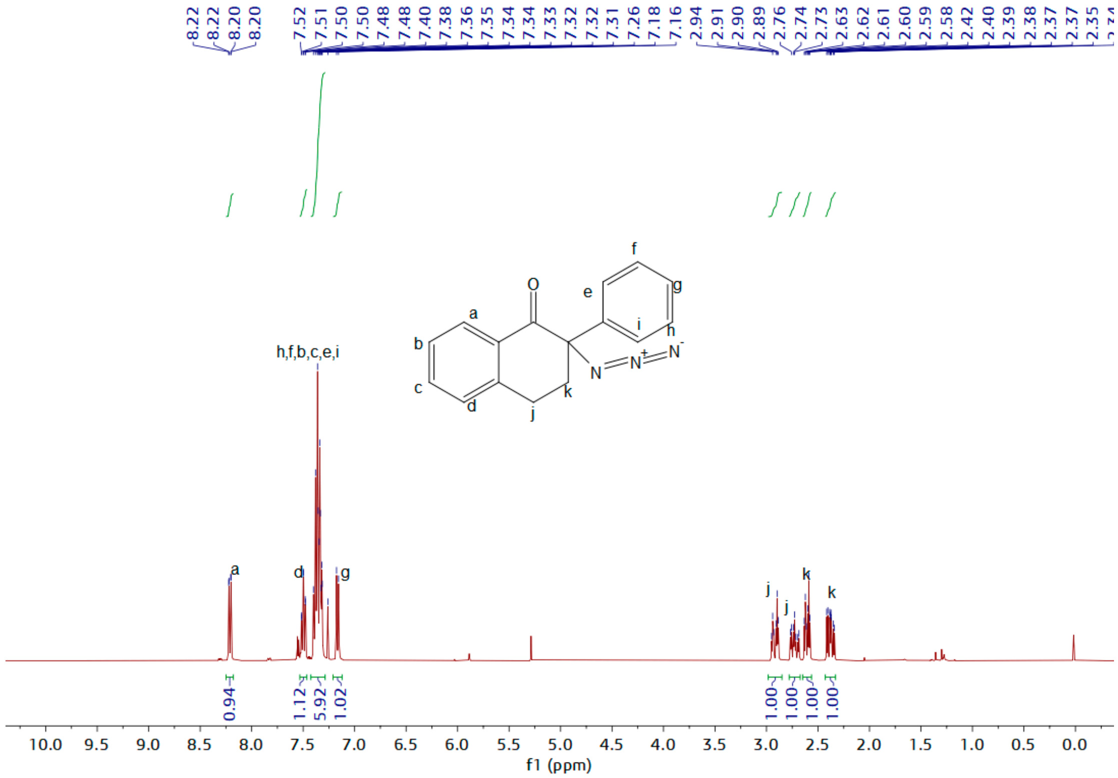

Figure S61 ^{1}H NMR spectrum of compound **33a** ($CDCl_3$, 400 MHz)

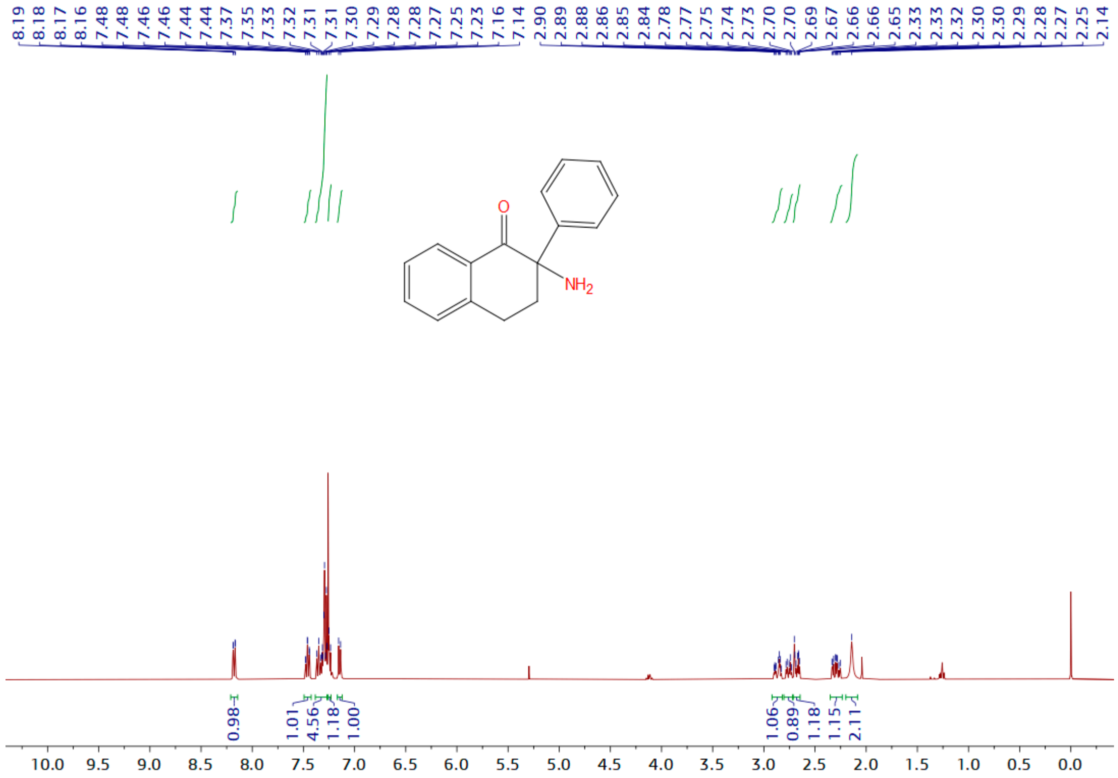

Figure S62 ^{1}H NMR spectrum of compound **31a** ($CDCl_3$, 400 MHz)

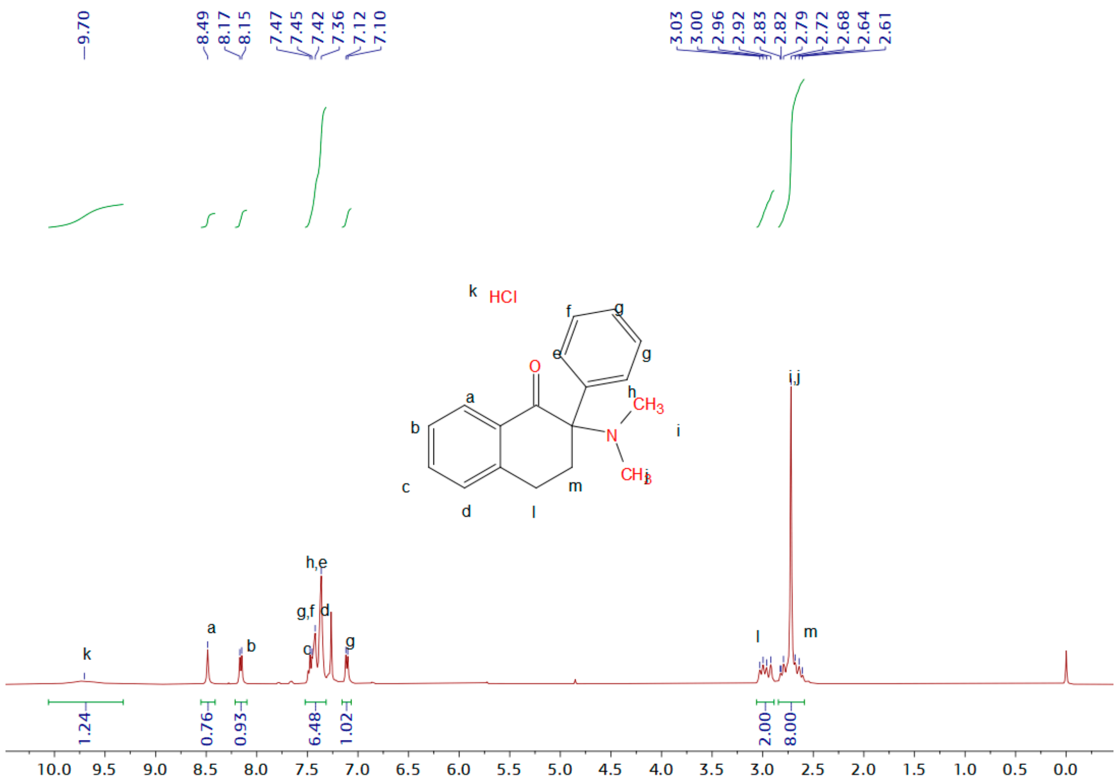

Figure S63 ^{1}H NMR spectsrum of compound **34** ($CDCl_3$, 400 MHz)

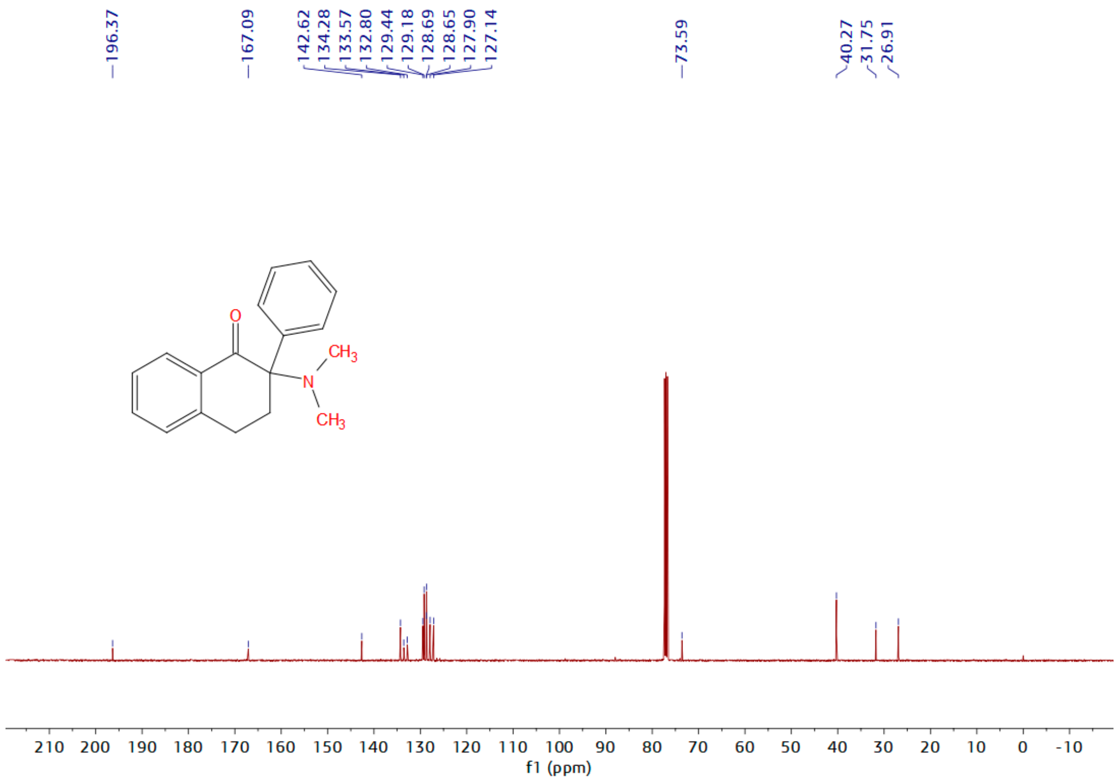

Figure S64 ^{13}C NMR spectrum of compound **34** ($CDCl_3$, 100 MHz)

Mass Spectrum SmartFormula Report

Analysis Info

Analysis Name	D:\Data\SHUJVFENXI\MADAWEIGROUP\2017195-LSY-7-37_BC2_01_31165.d	Acquisition Date	8/8/2022 6:18:22 AM
Method	20150915.m	Operator	BDAL@DE
Sample Name	2017195-LSY-7-37	Instrument / Ser#	maXis 4G 21240
Comment			

Acquisition Parameter

Source Type	ESI	Ion Polarity	Positive	Set Nebulizer	1.0 Bar
Focus	Not active	Set Capillary	4500 V	Set Dry Heater	220 °C
Scan Begin	50 m/z	Set End Plate Offset	-500 V	Set Dry Gas	6.0 l/min
Scan End	1500 m/z	Set Collision Cell RF	500.0 Vpp	Set Divert Valve	Waste

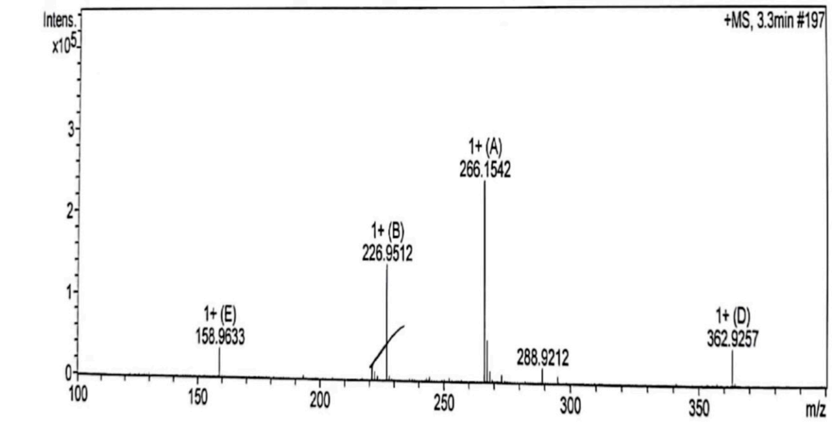

Meas. m/z	#	Formula	Score	m/z	err [ppm]	Mean err [ppm]	mSigma	rdb	e⁻ Conf	N-Rule
266.1542	1	C 18 H 20 N O	100.00	266.1539	-1.0	-1.5	18.0	9.5	even	ok

Figure S65 HRMS spectrum of compound **34**

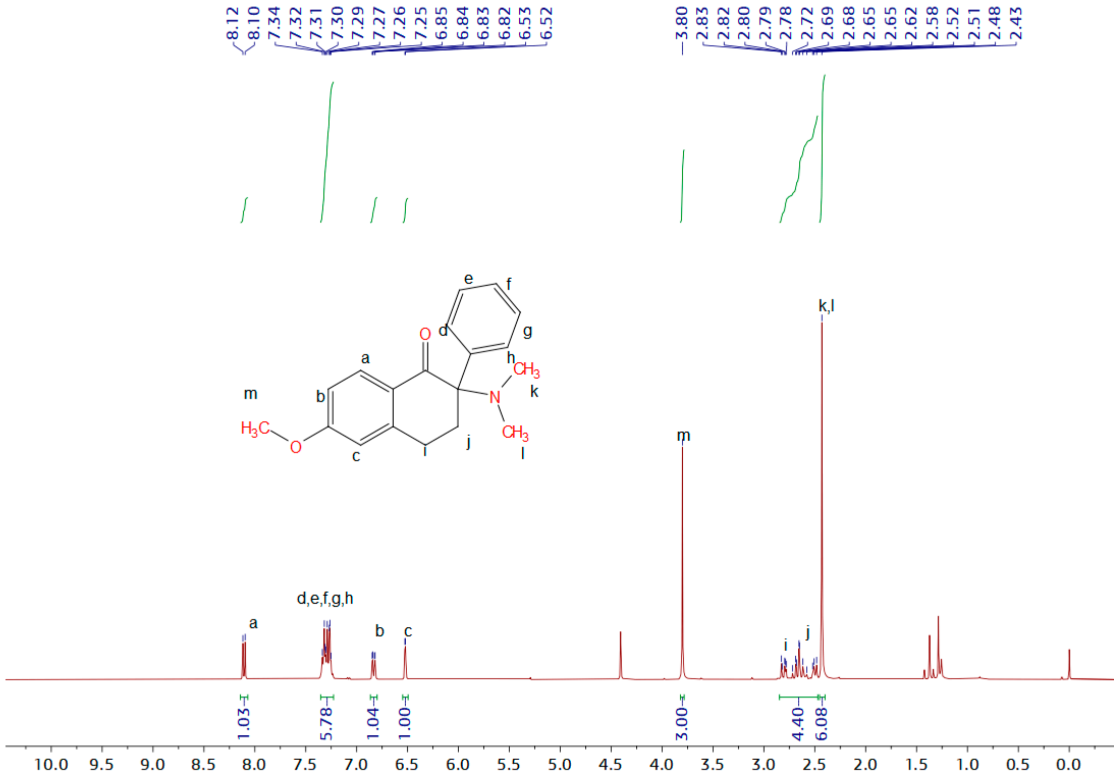

Figure S66 ^{1}H NMR spectsrum of compound **35** ($CDCl_3$, 400 MHz)

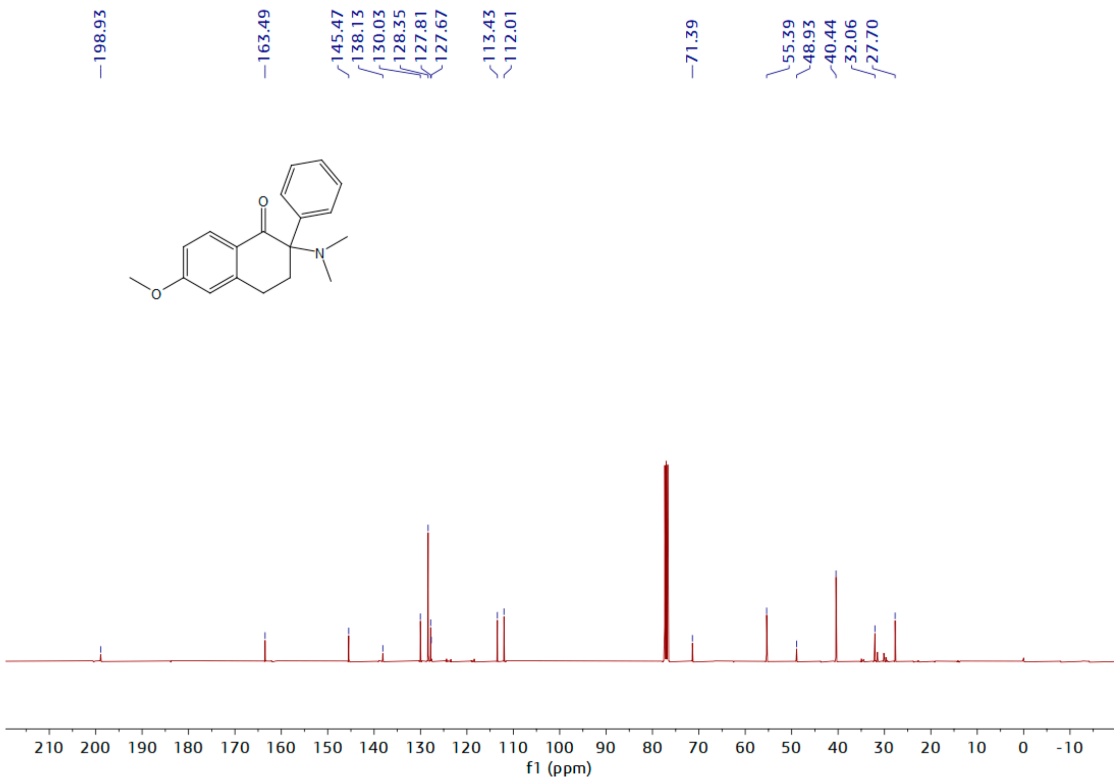

Figure S67 ^{13}C NMR spectrum of compound **35** ($CDCl_3$, 100 MHz)

Mass Spectrum SmartFormula Report

Analysis Info

Analysis Name	D:\Data\SHUJVFENXI\MADAWEIGROUP\2017195-LSY-6-100_RC3_01_31426.d	Acquisition Date	8/23/2022 7:19:13 AM
Method	20150915.m	Operator	BDAL@DE
Sample Name	2017195-LSY-6-100	Instrument / Ser#	maXis 4G 21240
Comment			

Acquisition Parameter

Source Type	ESI	Ion Polarity	Positive	Set Nebulizer	1.0 Bar
Focus	Not active	Set Capillary	4500 V	Set Dry Heater	220 °C
Scan Begin	50 m/z	Set End Plate Offset	-500 V	Set Dry Gas	6.0 l/min
Scan End	1500 m/z	Set Collision Cell RF	500.0 Vpp	Set Divert Valve	Waste

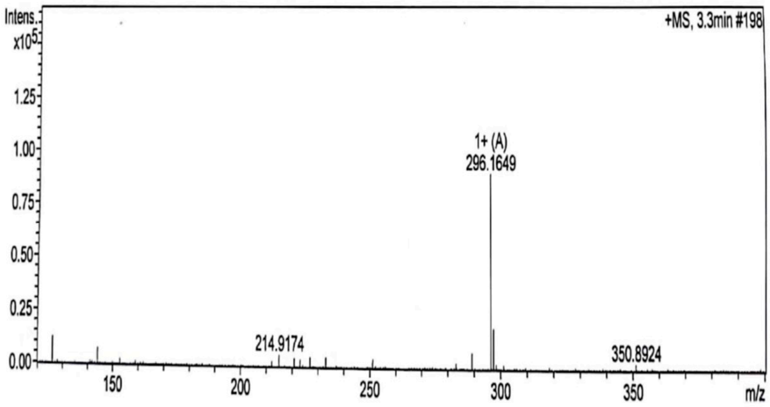

Meas. m/z	#	Formula	Score	m/z	err [ppm]	Mean err [ppm]	mSigma	rdb	e⁻ Conf	N-Rule
296.1649	1	C 19 H 22 N O 2	100.00	296.1645	-1.4	-1.2	3.5	9.5	even	ok

Figure S68 HRMS spectrum of compound **35**

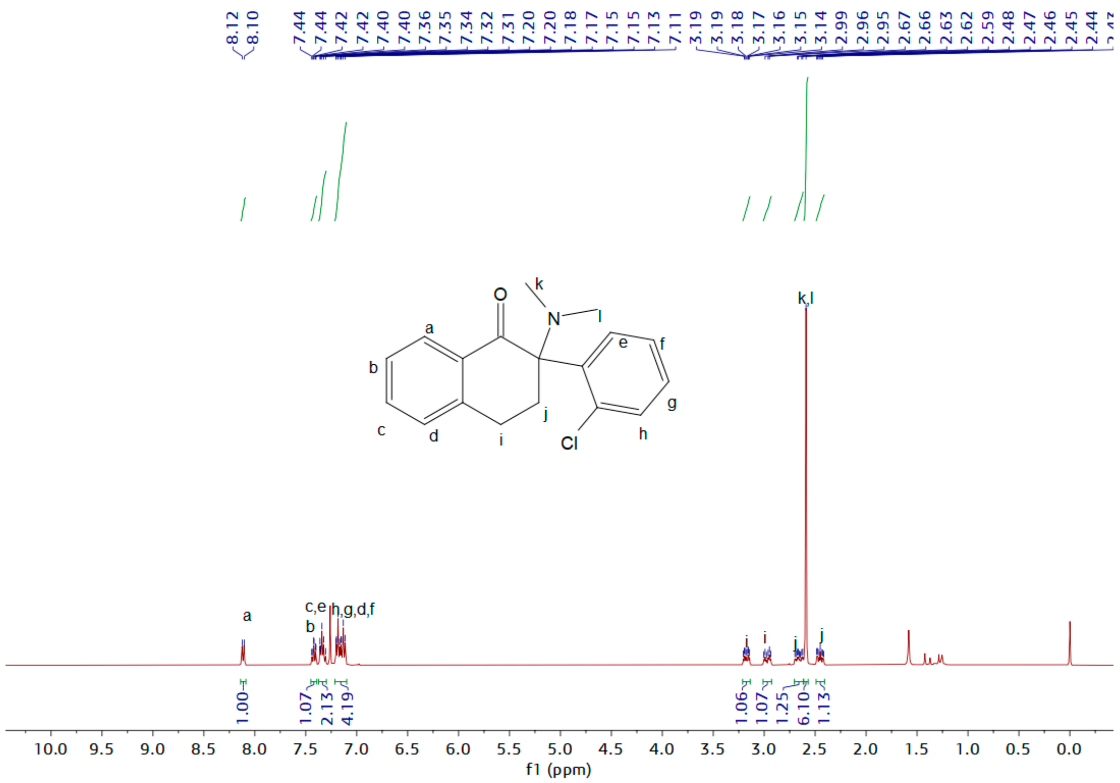

Figure S69 ^{1}H NMR spectsrum of compound **36** ($CDCl_3$, 400 MHz)

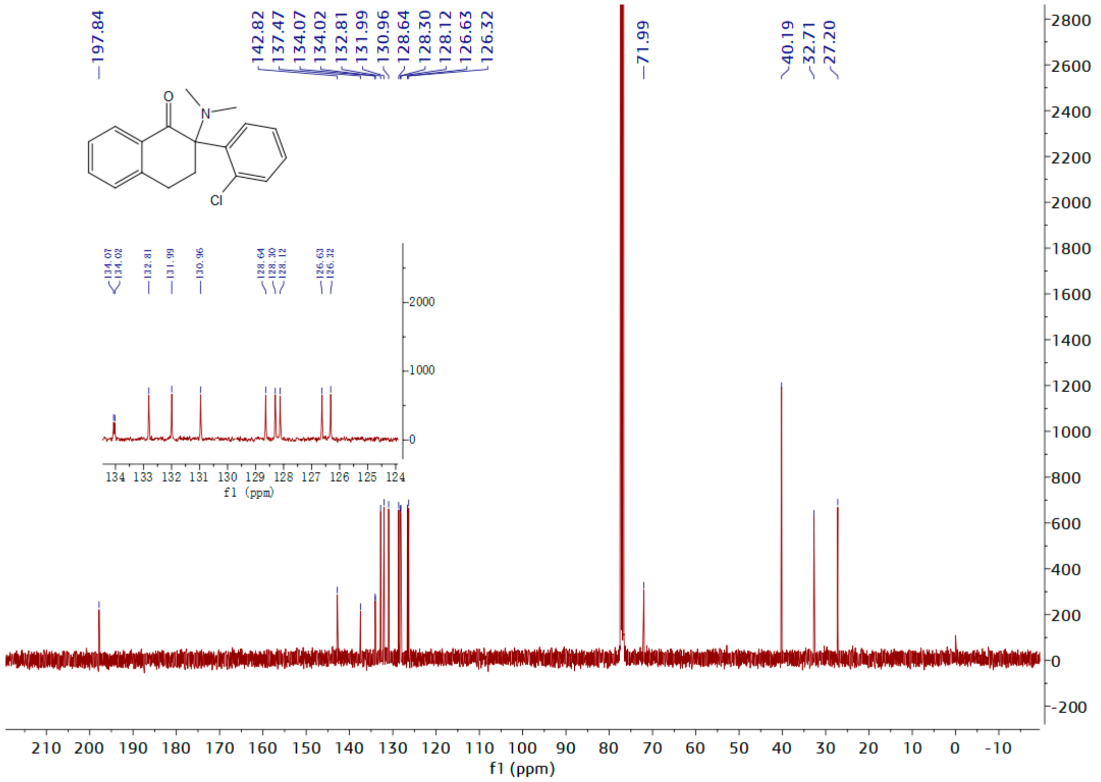

Figure S70 ^{13}C NMR spectrum of compound **36** ($CDCl_3$, 100 MHz)

Mass Spectrum SmartFormula Report

Analysis Info

Analysis Name	D:\Data\SHUJVFENXI\MADAWEIGROUP\2017195-LSY-6-59_RA4_01_31137.d	Acquisition Date	8/8/2022 3:28:35 AM
Method	20150915.m	Operator	BDAL@DE
Sample Name	2017195-LSY-6-59	Instrument / Ser#	maXis 4G 21240
Comment			

Acquisition Parameter

Source Type	ESI	Ion Polarity	Positive	Set Nebulizer	1.0 Bar
Focus	Not active	Set Capillary	4500 V	Set Dry Heater	220 °C
Scan Begin	50 m/z	Set End Plate Offset	-500 V	Set Dry Gas	6.0 l/min
Scan End	1500 m/z	Set Collision Cell RF	500.0 Vpp	Set Divert Valve	Waste

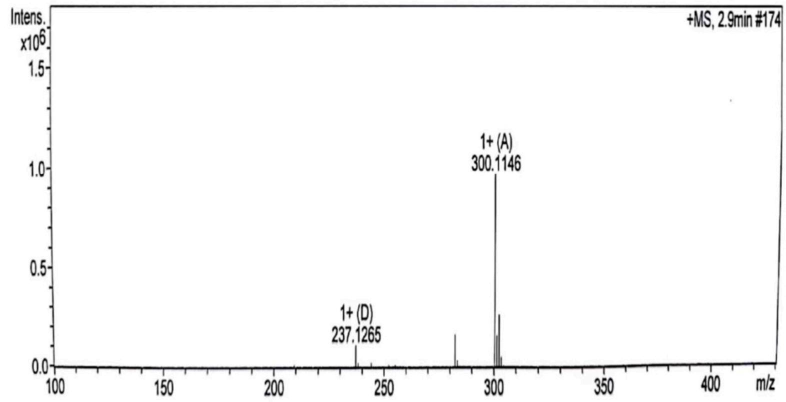

Meas. m/z	#	Formula	Score	m/z	err [ppm]	Mean err [ppm]	mSigma	rdb	e⁻ Conf	N-Rule
300.1146	1	C 18 H 19 Cl N O	100.00	300.1150	1.1	1.5	36.6	9.5	even	ok

Figure S71 HRMS spectrum of compound **36**

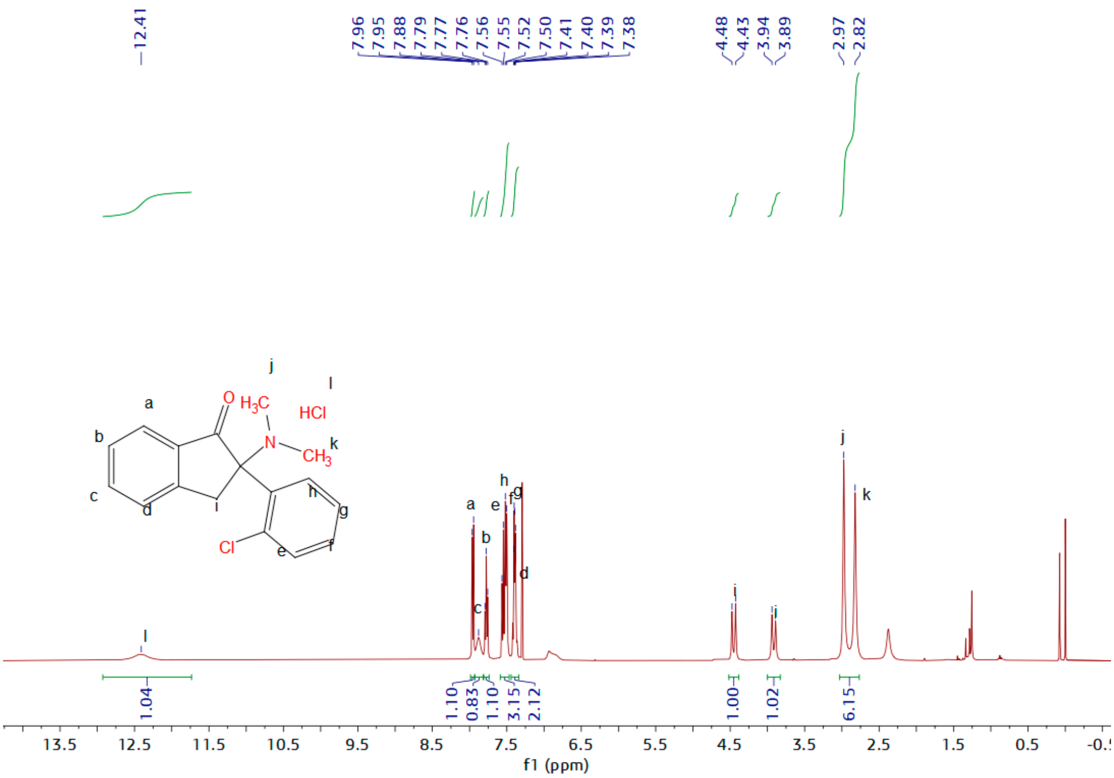

Figure S72 ^{1}H NMR spectsrum of compound **37** ($CDCl_3$, 400 MHz)

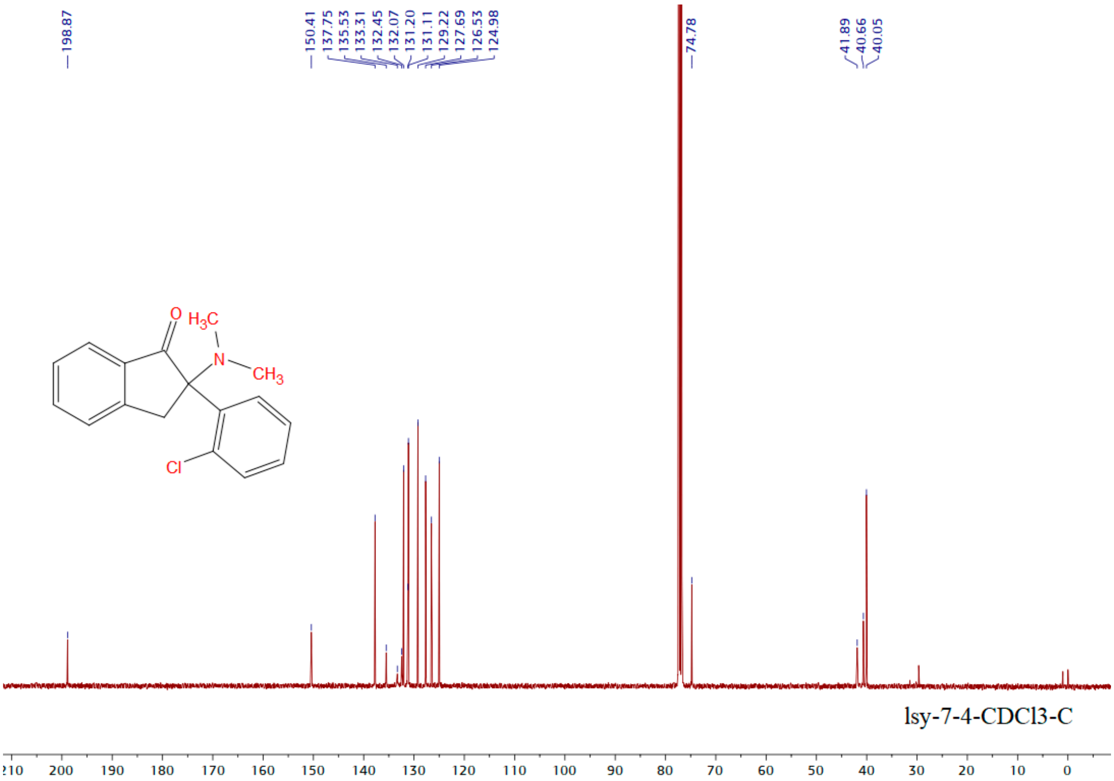

Figure S73 ^{13}C NMR spectrum of compound **37** ($CDCl_3$, 100 MHz)

Mass Spectrum SmartFormula Report

Analysis Info

Analysis Name	D:\Data\SHUJVFENXI\MADAWEIGROUP\2017195-LSY-7-4_RC6_01_31139.d	Acquisition Date	8/8/2022 3:40:40 AM
Method	20150915.m	Operator	BDAL@DE
Sample Name	2017195-LSY-7-4	Instrument / Ser#	maXis 4G 21240
Comment			

Acquisition Parameter

Source Type	ESI	Ion Polarity	Positive	Set Nebulizer	1.0 Bar
Focus	Not active	Set Capillary	4500 V	Set Dry Heater	220 °C
Scan Begin	50 m/z	Set End Plate Offset	-500 V	Set Dry Gas	6.0 l/min
Scan End	1500 m/z	Set Collision Cell RF	500.0 Vpp	Set Divert Valve	Waste

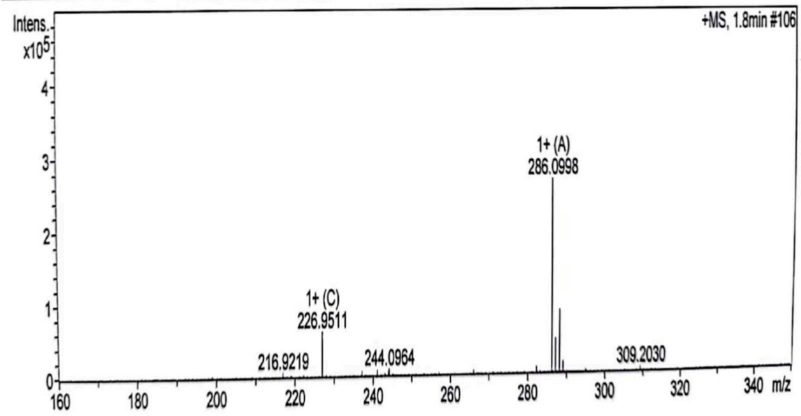

Meas. m/z	#	Formula	Score	m/z	err [ppm]	Mean err [ppm]	mSigma	rdb	e⁻ Conf	N-Rule
286.0998	1	C 17 H 17 Cl N O	100.00	286.0993	-1.8	-1.1	7.5	9.5	even	ok

Figure S74 HRMS spectrum of compound **37**

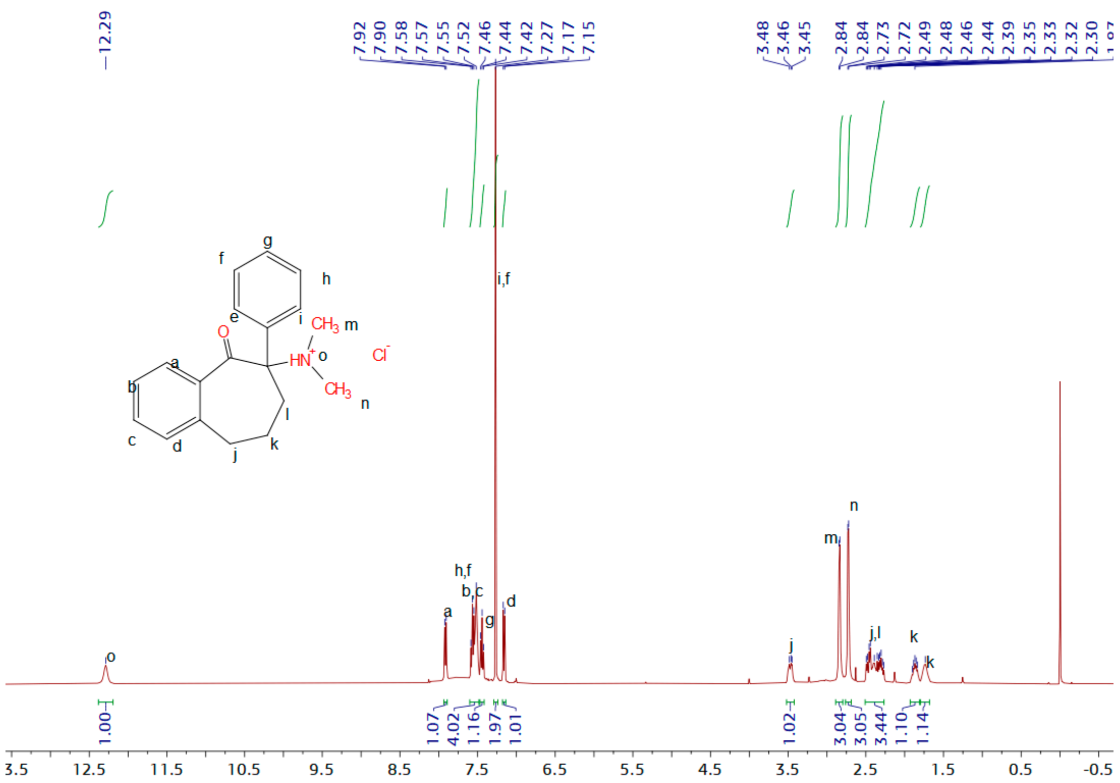

Figure S75 ^{1}H NMR spectrum of compound **38** ($CDCl_3$, 400 MHz)

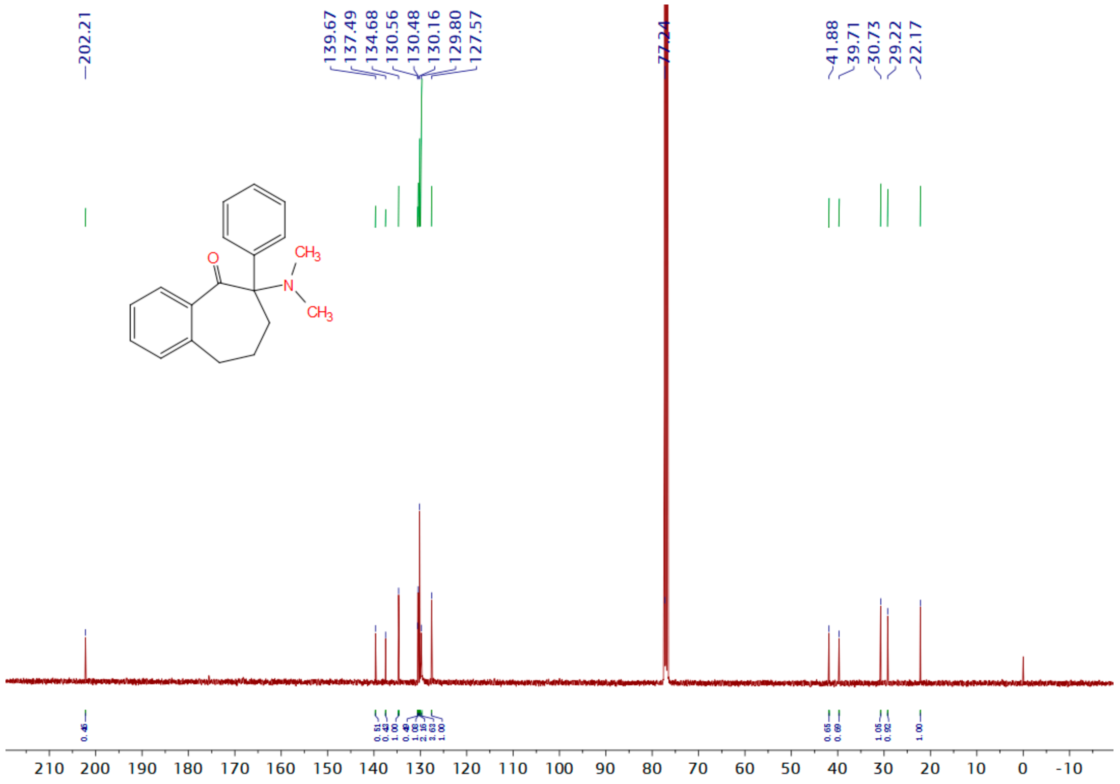

Figure S76 ^{13}C NMR spectrum of compound **38** ($CDCl_3$, 100 MHz)

Mass Spectrum SmartFormula Report

Analysis Info

Analysis Name	D:\Data\SHUJVFENXI\MADAWEIGROUP\2017195-LSY-7-43_RD2_01_31127.d	Acquisition Date	8/8/2022 2:24:50 AM
Method	20150915.m	Operator	BDAL@DE
Sample Name	2017195-LSY-7-43	Instrument / Ser#	maXis 4G 21240
Comment			

Acquisition Parameter

Source Type	ESI	Ion Polarity	Positive	Set Nebulizer	1.0 Bar
Focus	Not active	Set Capillary	4500 V	Set Dry Heater	220 °C
Scan Begin	50 m/z	Set End Plate Offset	-500 V	Set Dry Gas	6.0 l/min
Scan End	1500 m/z	Set Collision Cell RF	500.0 Vpp	Set Divert Valve	Waste

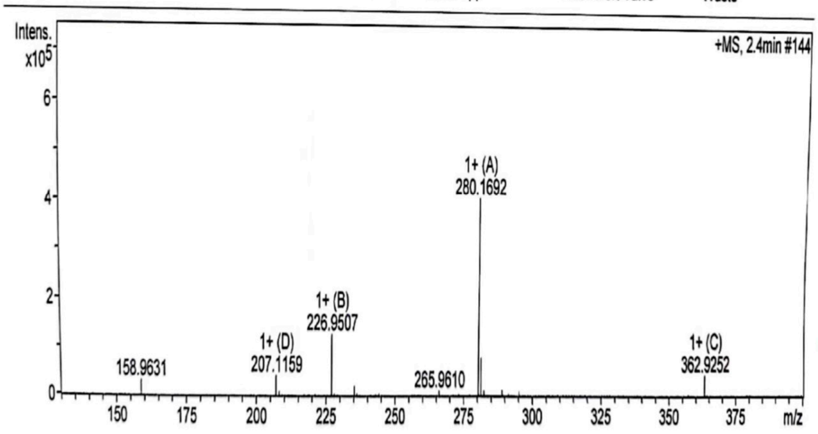

Meas. m/z	#	Formula	Score	m/z	err [ppm]	Mean err [ppm]	mSigma	rdb	e⁻ Conf	N-Rule
280.1692	1	C 19 H 22 N O	100.00	280.1696	1.3	1.5	10.0	9.5	even	ok

Figure S77 HRMS spectrum of compound **38**

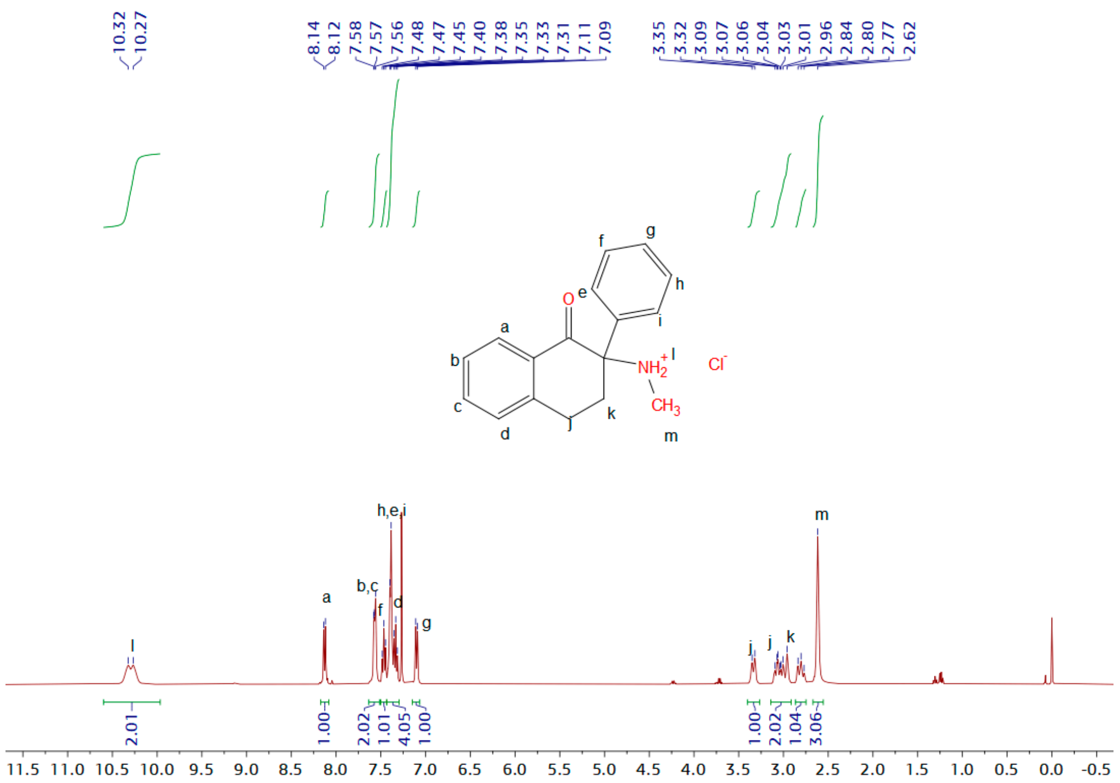

Figure S78 ^{1}H NMR spectrum of compound **41** ($CDCl_3$, 400 MHz)

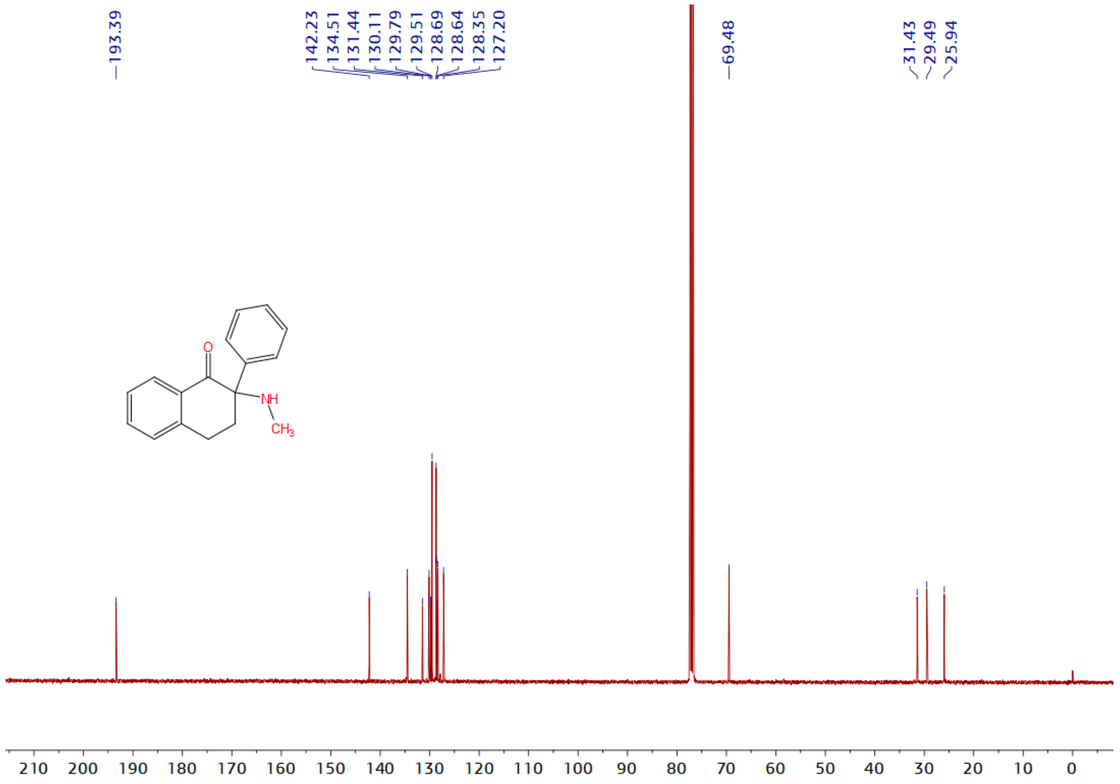

Figure S79 ^{13}C NMR spectrum of compound **41** ($CDCl_3$, 100 MHz)

Mass Spectrum SmartFormula Report

Analysis Info

Analysis Name	D:\Data\SHUJVFENXI\MADAWEIGROUP\2017195-LSY-7-47_RC8_01_31140.d	Acquisition Date	8/8/2022 3:46:57 AM
Method	20150915.m	Operator	BDAL@DE
Sample Name	2017195-LSY-7-47	Instrument / Ser#	maXis 4G 21240
Comment			

Acquisition Parameter

Source Type	ESI	Ion Polarity	Positive	Set Nebulizer	1.0 Bar
Focus	Not active	Set Capillary	4500 V	Set Dry Heater	220 °C
Scan Begin	50 m/z	Set End Plate Offset	-500 V	Set Dry Gas	6.0 l/min
Scan End	1500 m/z	Set Collision Cell RF	500.0 Vpp	Set Divert Valve	Waste

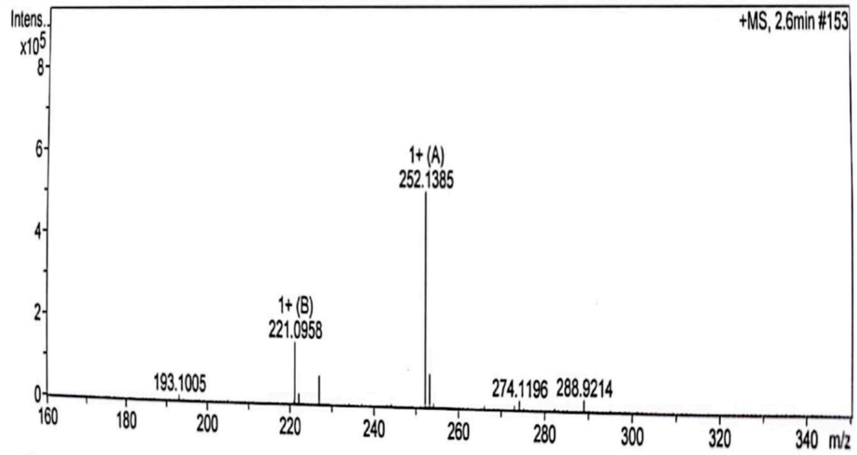

Meas. m/z	#	Formula	Score	m/z	err [ppm]	Mean err [ppm]	mSigma	rdb	e⁻ Conf	N-Rule
252.1385	1	C 17 H 18 N O	100.00	252.1383	-0.9	-0.5	19.5	9.5	even	ok

Figure S80 HRMS spectrum of compound **41**

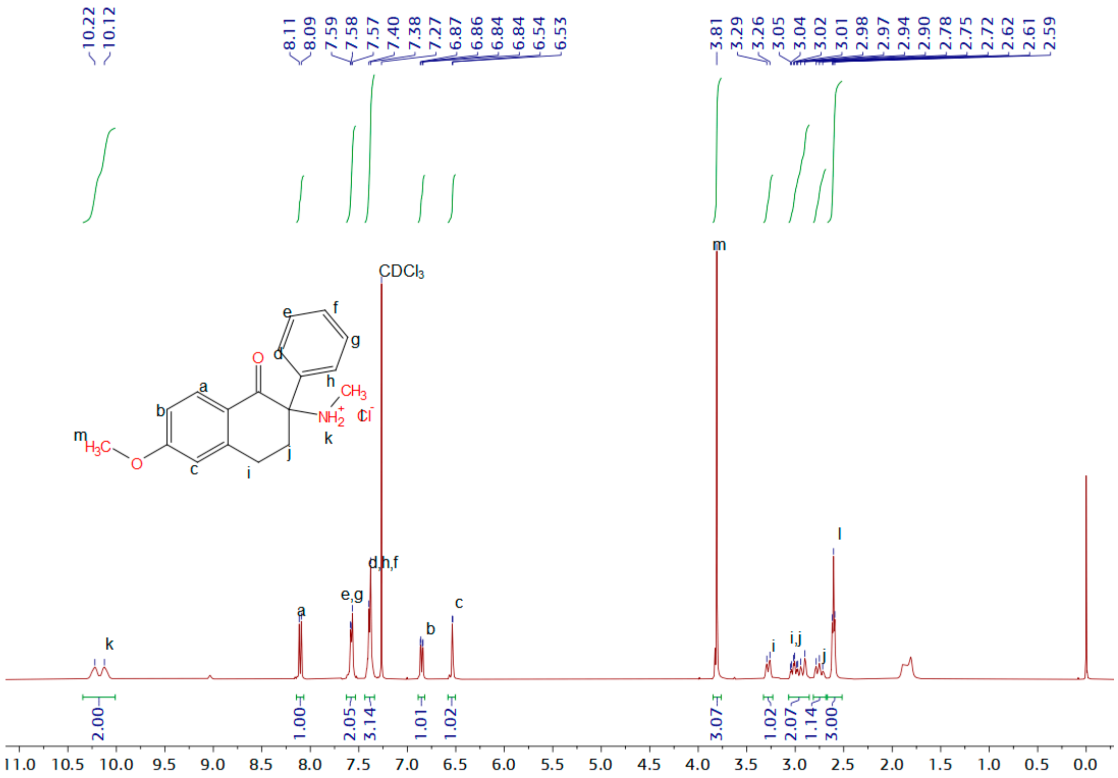

Figure S81 ^{1}H NMR spectrum of compound **42** ($CDCl_3$, 400 MHz)

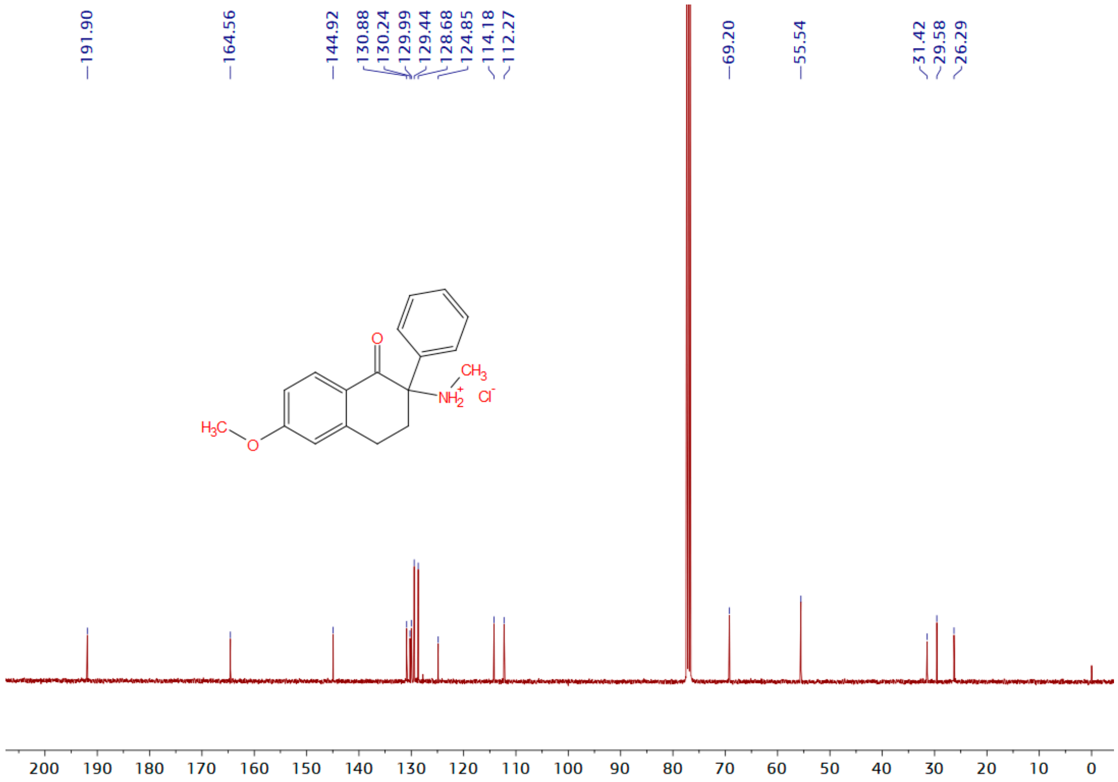

Figure S82 ^{13}C NMR spectrum of compound **42** ($CDCl_3$, 100 MHz)

Mass Spectrum SmartFormula Report

Analysis Info

Analysis Name	D:\Data\SHUJVFENXI\MADAWEIGROUP\2017195-LSY-6-100_RC3_01_31426.d	Acquisition Date	8/23/2022 7:19:13 AM
Method	20150915.m	Operator	BDAL@DE
Sample Name	2017195-LSY-6-100	Instrument / Ser#	maXis 4G 21240
Comment			

Acquisition Parameter

Source Type	ESI	Ion Polarity	Positive	Set Nebulizer	1.0 Bar		
Focus	Not active	Set Capillary	4500 V	Set Dry Heater	220 °C		
Scan Begin	50 m/z	Set End Plate Offset	-500 V	Set Dry Gas	6.0 l/min		
Scan End	1500 m/z	Set Collision Cell RF	500.0 Vpp	Set Divert Valve	Waste		

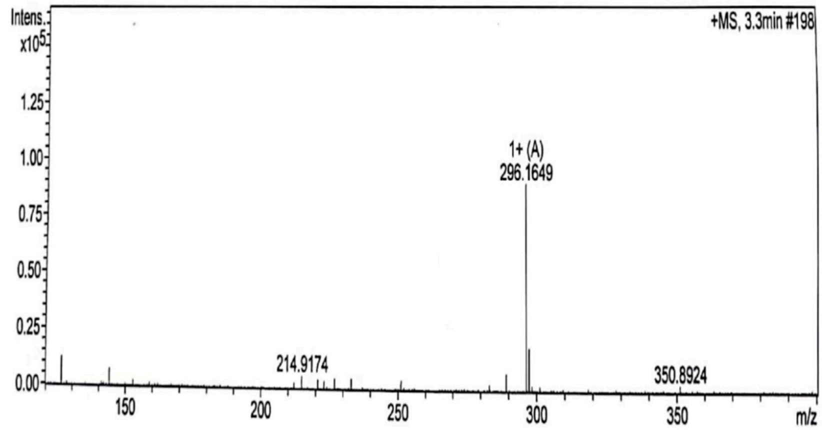

Meas. m/z	#	Formula	Score	m/z	err [ppm]	Mean err [ppm]	mSigma	rdb	e⁻ Conf	N-Rule
296.1649	1	C 19 H 22 N O 2	100.00	296.1645	-1.4	-1.2	3.5	9.5	even	ok

Figure S83 HRMS spectrum of compound **42**

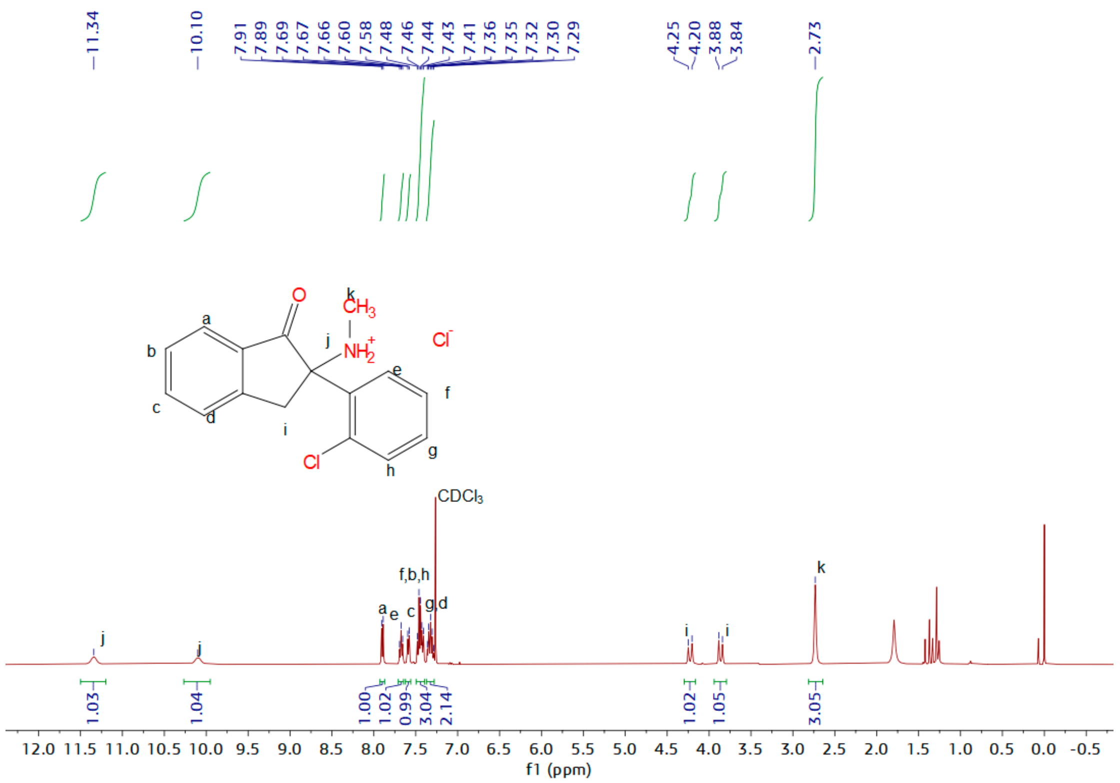

Figure S84 ^{1}H NMR spectrum of compound **43** ($CDCl_3$, 400 MHz)

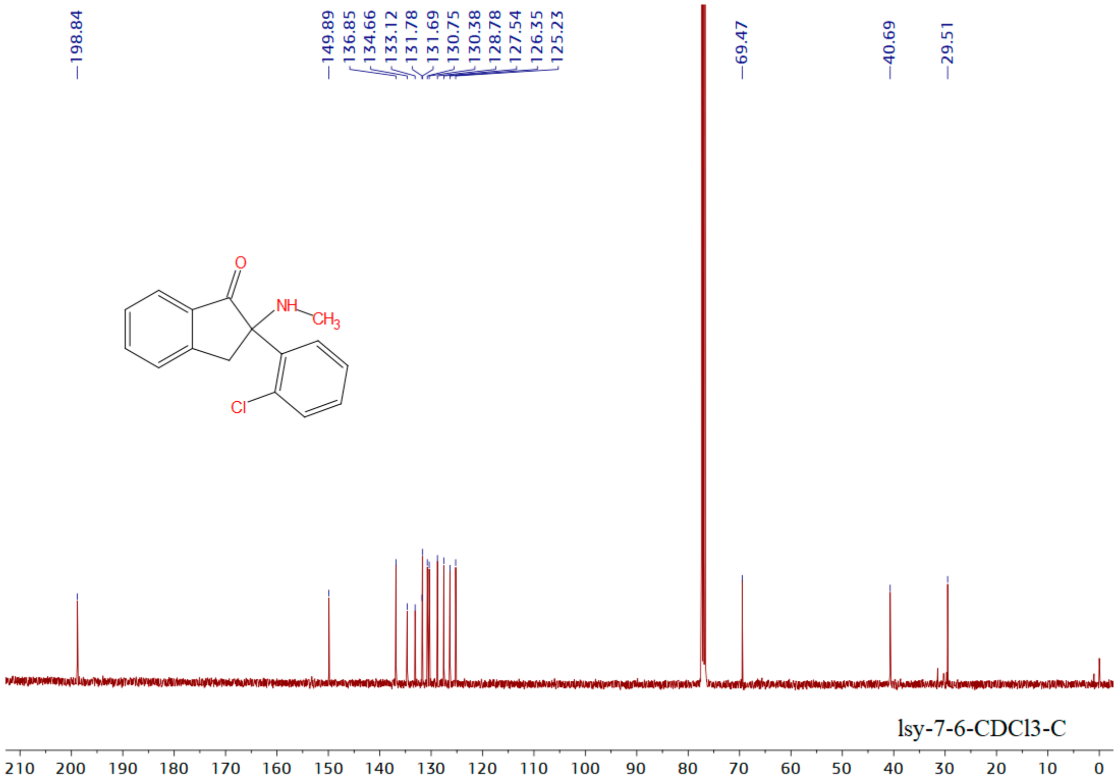

Figure S85 ^{13}C NMR spectrum of compound **43** ($CDCl_3$, 100 MHz)

Mass Spectrum SmartFormula Report

Analysis Info

Analysis Name	D:\Data\SHUJVFENXI\MADAWEIGROUP\2017195-LSY-7-6_RC5_01_31428.d	Acquisition Date	8/23/2022 7:32:04 AM
Method	20150915.m	Operator	BDAL@DE
Sample Name	2017195-LSY-7-6	Instrument / Ser#	maXis 4G 21240
Comment			

Acquisition Parameter

Source Type	ESI	Ion Polarity	Positive	Set Nebulizer	1.0 Bar
Focus	Not active	Set Capillary	4500 V	Set Dry Heater	220 °C
Scan Begin	50 m/z	Set End Plate Offset	-500 V	Set Dry Gas	6.0 l/min
Scan End	1500 m/z	Set Collision Cell RF	500.0 Vpp	Set Divert Valve	Waste

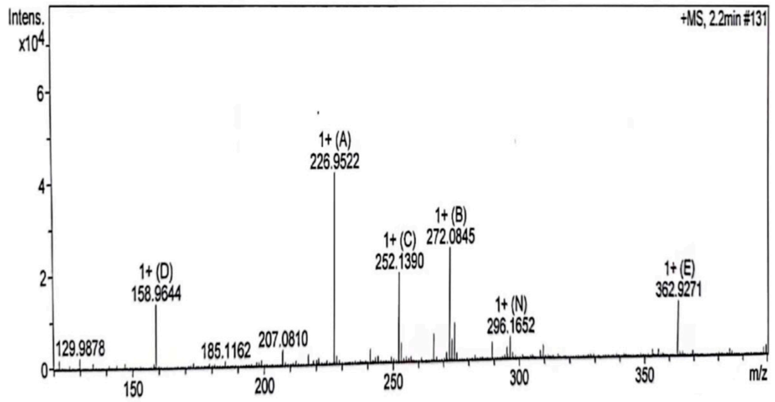

Meas. m/z	#	Formula	Score	m/z	err [ppm]	Mean err [ppm]	mSigma	rdb	e⁻ Conf	N-Rule
272.0845	1	C 16 H 15 Cl N O	100.00	272.0837	-3.2	-3.2	5.6	9.5	even	ok

Figure S86 HRMS spectrum of compound **43**
